# Amides of moronic acid and morolic acid with the tripeptides MAG and GAM targeting antimicrobial, antiviral and cytotoxic effects†

**DOI:** 10.1039/d4md00742e

**Published:** 2024-10-29

**Authors:** Uladzimir Bildziukevich, Lucie Černá, Jana Trylčová, Marie Kvasnicová, Lucie Rárová, David Šaman, Petra Lovecká, Jan Weber, Zdeněk Wimmer

**Affiliations:** a Institute of Experimental Botany of the Czech Academy of Sciences, Isotope Laboratory Vídeňská 1083 14220 Prague 4 Czech Republic wimmer@biomed.cas.cz; b Department of Biochemistry and Microbiology, University of Chemistry and Technology in Prague Technická 5 16628 Prague 6 Czech Republic; c Department of Chemistry of Natural Compounds, University of Chemistry and Technology in Prague Technická 5 16628 Prague 6 Czech Republic zdenek.wimmer@vscht.cz; d Institute of Organic Chemistry and Biochemistry of the Czech Academy of Sciences Flemingovo náměstí 2 16610 Prague 6 Czech Republic; e Laboratory of Growth Regulators, Faculty of Science, Palacký University, and Institute of Experimental Botany of the Czech Academy of Sciences Šlechtitelů 27 CZ-77900 Olomouc Czech Republic; f Department of Experimental Biology, Faculty of Science, Palacký University Šlechtitelů 27 CZ-77900 Olomouc Czech Republic

## Abstract

A series of amides of selected plant triterpenoids, moronic acid and morolic acid, with the tripeptides MAG and GAM, was designed and synthesized. Two required tripeptides 5 and 10 were synthesized by a step-wise chain elongation of the ethyl esters of either glycine or l-methionine at their N-terminus using Boc-protected amino acids in each step. The tripeptides 5 and 10 were used for the synthesis of 13–23, the derivatives of moronic acid (11) and morolic acid (12), to get a series of amide derivatives of the less frequently studied triterpenoids 11 and 12. The target compounds, and their intermediates, were subjected to an investigation of their antimicrobial, antiviral and cytotoxic activity. Selectivity of the pharmacological effects was found. Generally, the target compounds inhibited only the G^+^ microorganisms. Compound 16 inhibited *Staphylococcus aureus* (*I* = 99.6%; *c* = 62.5 μM) and *Enterococcus faecalis* (*I* = 85%; *c* = 250 μM). Several compounds showed moderate antiviral effects, both anti-HIV-1, 19 (EC_50_ = 57.0 ± 4.1 μM, CC_50_ > 100 μM), 20 (EC_50_ = 17.8 ± 2.1 μM, CC_50_ = 41.0 ± 5.2 μM) and 23 (EC_50_ = 12.6 ± 0.82 μM, CC_50_ = 38.0 ± 4.2 μM), and anti-HSV-1, 22 (EC_50_ = 27.7 ± 3.5 μM, CC_50_ > 100 μM) and 23 (EC_50_ = 30.9 ± 3.3 μM, CC_50_ > 100 μM). The target compounds showed no cytotoxicity in cancer cells, however, several of their intermediates were cytotoxic. Compound 21 showed cytotoxicity in HeLa (IC_50_ = 7.9 ± 2.1 μM), G-361 (IC_50_ = 8.0 ± 0.6 μM) and MCF7 (IC_50_ = 8.6 ± 0.2 μM) cancer cell lines, while being non-toxic in normal fibroblasts (BJ; IC_50_ > 50 μM).

## Abstract 2

The most successful structures showing their antimicrobial, antiviral or cytotoxic effects. Selectivity of their effects has been observed.

## Graphical abstract

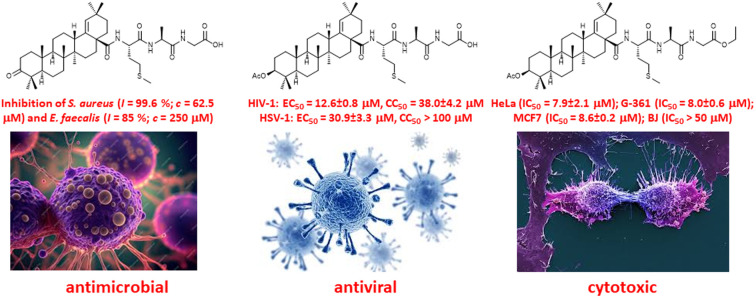

## 1. Introduction

Three main threats to human health exist at present: viral and bacterial infections, and cancer, and all of them can be potentially fatal.^1–3^ Viral infections represent the most important and the most frequent infectious diseases worldwide.^1^ Viruses invading the human body generally belong to two categories, either viruses being long-term parasites in the human body, *e.g.*, herpes virus, hepatitis B/C virus, human influenza virus *etc.*, or viruses being long-term parasites in animals living close to humans, *e.g.*, chickens, dogs, pigs *etc.* Cancer is another frequently occurring threat causing the death of a significant part of the human population.^2^ Finally, bacterial infections should be mentioned as an additional important affliction affecting human organisms, often accompanied by a wide resistance to the currently used antimicrobials.^3^

Within the field of viral infections, acquired immunodeficiency syndrome (AIDS) is a disease of the cell-mediated immune system or T-lymphocytes of the human body. In AIDS, the number of the T-cells is reduced, and therefore, the production of antibodies by the B-cells is directly stimulated. Consequently, the natural defence system of the body against AIDS is destroyed. In addition, other immune cells, such as monocyte–phagocytes, B-lymphocytes, and natural killer cells, are also damaged to varying degrees, which promotes the occurrence of various serious infections and tumours.^1^ Generally, the life cycle of HIV-1 includes adsorption, fusion, reverse transcription, integration, transcription, translation, and assembly.^1^ Studies have identified triterpenoids with anti-HIV-1 activity, and most compounds can act on multiple key HIV enzymes.^1^ Herpes viruses (HSV) causing human infections include herpes simplex virus HSV-1 and HSV-2.^1^ The herpes virus can infect any organ or tissue in the human body to cause a variety of diseases,^1^ and this infection has recently become one of the main causes of death in people with impaired immune function. Presently, there are still no anti-HSV drugs with high efficiency and specificity, and showing no side effects.^1^

Considering the above mentioned two types of viruses, cancer and microbial infections, focusing on plant triterpenoids as a natural source of potential active agents seems to be a logical target in a search for novel pharmacologically important agents. Morolic acid and moronic acid are rare in the nature, and have not yet been frequently studied. However, we have recently published a series of their cytotoxic derivatives showing nano-assembly characteristics.^4^ Many basic items of information about these two triterpenoids, including their biological effects, have been mentioned therein.^4^ Morolic acid and moronic acid belong among the oleanane triterpenoids. Like all similar plant secondary metabolites, they appear as conjugates, mostly with oligosaccharides, *i.e.*, saponins, in which form their solubility in water or in the physiological media is acceptably high in natural resources.^5^ Natural saponins, triterpenoid glycosides, have recently been investigated as potential SARS-CoV-2 inhibitors.^6^ Generally, saponins are relatively unstable compounds, easy to destroy by liberating the non-polar aglycone (sapogenin), mainly during the isolation processes. An efficient way to make investigation of the biological activity of these triterpenoids easier is to prepare their semisynthetic conjugates, both of polar or non-polar nature, for different types of practical pharmacological applications.

One of the ways to make structural modifications to triterpenoids consists in producing their conjugates with tripeptides capable of modifying the triterpenoid characteristics in other ways than are done using (oligo)saccharides.^7^ The tripeptide GAM has been known as a part of the hydrophobic hexapeptide GAMVVH displaying high angiotensin-converting enzyme inhibitory activity.^8^ It has also been known from human erythroleukemia cells (HEL), where it appears as an N-terminal tripeptide of des-acyl ghrelin.^9^ Ghrelin is a hormone produced by enteroendocrine cells of the gastrointestinal tract, especially the stomach, and it is often called a “hunger hormone” for its ability to increase food intake.^10^ It activates cells in the anterior pituitary gland and hypothalamic arcuate nucleus, including neuropeptide Y neurons that initiate appetite.^10^ Ghrelin stimulates brain structures having a specific receptor, the growth hormone secretagogue receptor 1A (GHSR-1A).^10^ It also participates in regulation of reward cognition and behaviour, learning and memory, the sleep–wake cycle, taste sensation, and glucose metabolism.^10^ Ghrelin has been described to recognize several receptor targets, and to display a multifaceted anti-oxidative, anti-inflammatory and immunomodulatory activity that could limit the severity of the SARS-CoV-2 infection: ghrelin can down-regulate the nuclear factor (NF-κB) and up-regulate the peroxisome proliferator-activated receptor gamma (PPAR-γ) and the gene Nrf2 expression, leading to a repression in cytokine storm and oxidative stress.^11^ The tripeptide MAG has been found in the macroalga *Palmaria palmate* as a part of a heptapeptide MAGVDHI showing inhibitory activity in dipeptidyl peptidase.^12^

## 2. Rational design

Short tripeptides used in the structural modification of triterpenoids will introduce lower polarity to the target molecules than oligosaccharides which are the natural conjugating units of triterpenoids in plants. Based on our previous investigation, we have found that amide derivatives of triterpenoids often display cytotoxicity and antimicrobial activity,^13–16^ and triterpenoid conjugates that are generally low polarity ones may display antiviral activity.^17,18^ The previously achieved results triggered our search for the types of pharmacological activity that could potentially be displayed by the suggested tripeptide derivatives of the selected triterpenoids. The synthetic strategy is described below.

Therefore, based on the literature data available so far, and on our recent results,^4,13–18^ the objectives of this investigation were set as follows: (a) designing and synthesizing tripeptides GAM and MAG; (b) designing and developing conjugates of moronic acid and morolic acid with the tripeptides GAM and MAG to synthesize novel agents potentially capable of displaying antimicrobial, antiviral and/or cytotoxic activity; (c) performing and evaluating antimicrobial, antiviral (anti-HIV-1 and anti-HSV-1) and cytotoxic effects of the target novel compounds and their synthetic intermediates, to suggest structure–activity relationships, and to evaluate potential selectivity in their pharmacological effects.

## 3. Results and discussion

### 3.1. Synthetic procedures and structure elucidation

The synthetic procedures described here were newly developed, although based on our previous experience with the formation of analogous new chemical bonds.^4,13–18^

**Scheme 1:**
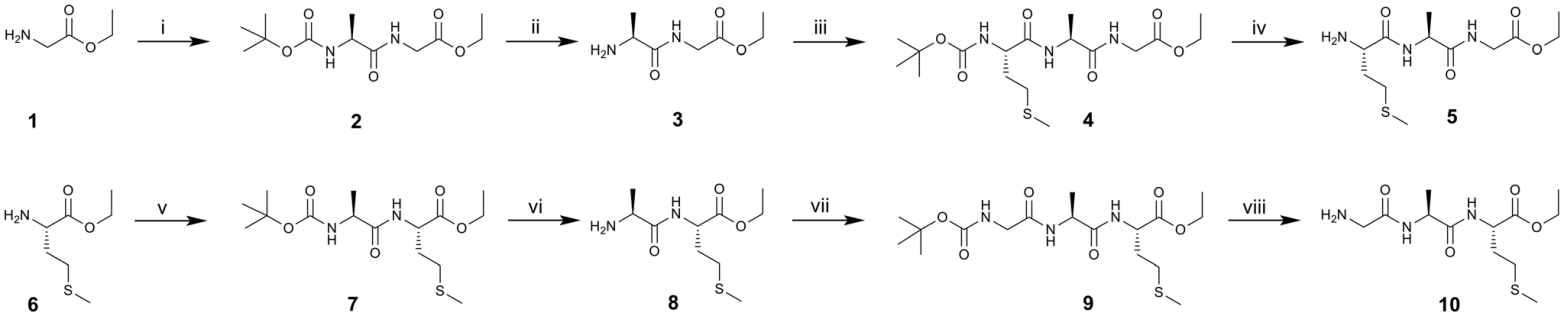
Synthetic procedure 1. Reagents and reaction conditions: i: *N*-Boc-l-Ala-OH, T3P, pyridine, 0 °C; ii: HCl (g) in 1,4-dioxane, 30–35 °C; iii: *N*-Boc-l-Met-OH, T3P, pyridine, 0 °C; iv: HCl (g) in 1,4-dioxane, 30–35 °C; v: *N*-Boc-l-Ala-OH, T3P, pyridine, r.t.; vi: HCl (g) in 1,4-dioxane, 30–35 °C; vii: *N*-Boc-Gly-OH, T3P, r.t.; viii: HCl (g) in 1,4-dioxane, 30–35 °C.

A convenient derivative of the former required tripeptide, ethyl l-methionyl-l-alanylglycinate (5, Scheme 1) was synthesized from glycine ethyl ester (1), the reaction of which with *N*-Boc-l-alanine in pyridine, using T3P as dehydration agent, 2 was prepared. The protecting Boc-group in 2 was removed by HCl (g) in 1,4-dioxane, yielding 3. A subsequent reaction of 3 with *N*-Boc-l-methionine in pyridine, using T3P as a dehydration agent, afforded 4. The protecting Boc-group in 4 was again removed by HCl (g) in 1,4-dioxane, yielding 5, the first of the required tripeptides. The synthetic procedure for the preparation of a convenient derivative of the second required tripeptide, ethyl glycyl-l-alanyl-l-methioninate (10, Scheme 1) is analogous, starting from l-methionine ethyl ester (6). The intermediate 7 was synthesized by the reaction of 6 with *N*-Boc-l-alanine in pyridine, using T3P as dehydration agent. The protecting Boc-group in 7 was removed by HCl (g) in 1,4-dioxane, yielding 8. A subsequent reaction of 8 with *N*-Boc-glycine in pyridine, using T3P as dehydration agent, afforded 9. The protecting Boc-group in 9 was removed by HCl (g) in 1,4-dioxane, yielding 10. All compounds showed an amorphous character.

**Scheme 2:**
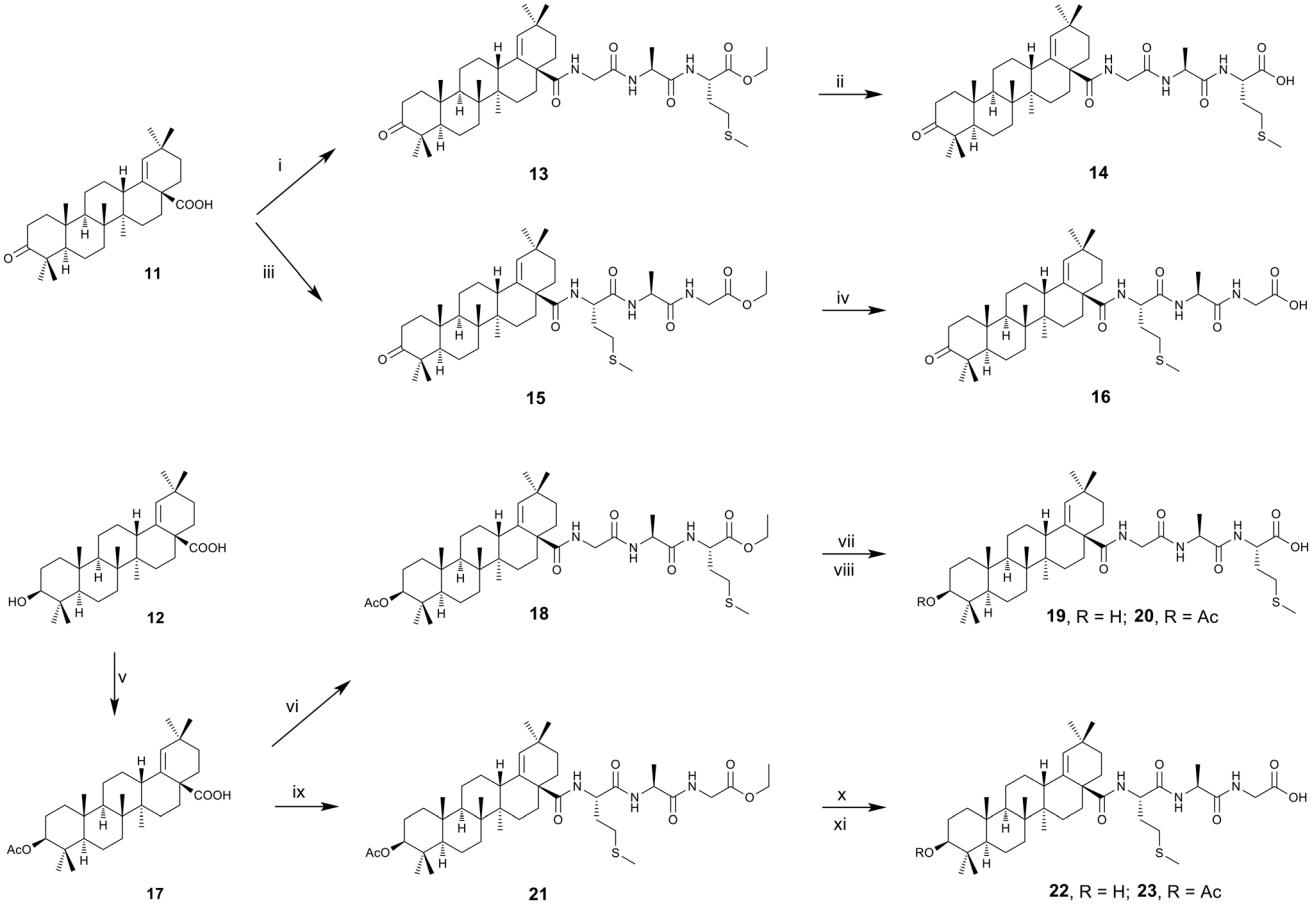
Synthetic procedure 2. Reagents and reaction conditions: i: a) oxalyl chloride, DCM, r.t., b) 10, DIPEA, DCM, r.t.; ii: LiOH·H_2_O, MeOH, r.t.; iii: a) oxalyl chloride, DCM, r.t., b) 5, DIPEA, DCM, r.t.; iv: LiOH·H_2_O, MeOH, r.t.; v: Ac_2_O, Et_3_N, DMAP, THF, reflux; vi: a) oxalyl chloride, DCM, r.t., b) 10, DIPEA, DCM, r.t.; vii (to get 19): a) LiOH·H_2_O (1.5 eq.), MeOH, r.t.; b) LiOH·H_2_O (3 eq.), MeOH, reflux; viii (to get 20): LiOH·H_2_O (1 eq.), MeOH, r.t.; ix: a) oxalyl chloride, DCM, r.t., b) 5, DIPEA, DCM, r.t.; x (to get 22): a) LiOH·H_2_O (1.5 eq.), MeOH, r.t.; b) LiOH·H_2_O (6.5 eq.), MeOH, reflux; xi (to get 23): LiOH·H_2_O (1 eq.), MeOH, r.t.

The next pair of target compounds (14 and 16; Scheme 2) was synthesized from 11 that was initially converted to its acyl chloride using oxalyl chloride in DCM. The prepared acyl chloride was used as a crude intermediate product in the reactions either with 10 or 5, using DIPEA as a base, affording the respective products 13 and 15. The terminal ester groups in 13 or 15 were removed by alkaline hydrolysis, leaving free terminal carboxyl groups in the required products 14 and 16, respectively. Another pair of target compounds (19 and 22; Scheme 2) was synthesized from 12 that was initially also converted to its C(3)-acetyloxy derivative to protect the C(3)–OH group, affording 17. Starting from 17, the synthetic procedure was analogous to that described above: 17 was converted to its acyl chloride using oxalyl chloride in DCM, the acyl chloride was used as a crude product in the reactions either with 10 or 5, using DIPEA as a base, affording the respective products 18 and 21. The terminal ester groups and the acetyl group from the C(3)-acetyloxy groups in 18 or 21 were removed by alkaline hydrolysis in a boiling solution of LiOH in methanol using a Dimroth condenser, leaving terminal carboxyl groups and the C(3)–OH groups both free in the required products 19 and 22, respectively. In addition, we found it useful to subject an additional pair of the target compounds (20 and 23) to the antimicrobial, antiviral and cytotoxicity screening tests as well. Therefore, the respective intermediates 18 and 21 were subjected to a mild alkaline hydrolysis with a methanol solution of LiOH, affording the required compounds 20 and 23, respectively (Scheme 2).

The structures of all the compounds were elucidated by combining the data from NMR spectroscopy, MS and IR data. The ^1^H and ^13^C NMR data were checked using both 1D and 2D NMR spectra (see Experimental section). For the 2D NMR data, the combination of gHSQC and gHMBC spectra confirmed the presented amino acid sequence, as well as the synthesized C–N bond with the triterpenoid skeleton. The ESI shows the scanned 1D NMR spectra only. The MS and IR spectra support the structure elucidation based mainly on the NMR spectra. Due to the relatively small quantities of the final compounds, specific optical rotation data are not available. Purity of all prepared compounds was checked by HPLC, and was found ≥99% for all compounds.

### 3.2. Antimicrobial activity

Antimicrobial activity was tested in the three concentrations of the studied compounds, *c* = 250 μM, 125 μM and 62.5 μM, using either vancomycin (10 mg mL^−1^; for the G^+^ microorganisms) or kanamycin (10 mg mL^−1^; for the G^−^ microorganisms) as the positive references. The compounds were tested in *Staphylococcus aureus* (G^+^), *Enterococcus faecalis* (G^+^), *Pseudomonas aeruginosa* (G^−^) and *Escherichia coli* (G^−^) using the dilution and resazurin tests. The results of the dilution test revealed that the tested compounds were active on the G^+^ microorganisms. Compound 16 displayed 99.6% inhibition effect in *S. aureus* (*c* = 62.5 μM) and 85% inhibition effect on *Ent. faecalis* (*c* = 250 μM). Compound 15 was the other active derivative of 11, however, it displayed a lower antimicrobial effect in both microorganisms than 11. Finally, compounds 18 and 20–22 derived from morolic acid (12) showed a higher inhibition effect in *S. aureus* than 12 in the dilution test (Table 1). A comparison of the antimicrobial effects of the promising compounds with their parent triterpenoids is important, because 15 and 16 are structurally derived from 11, while 18 and 20–22 are the derivatives of 12.

**Table 1:** Inhibition of *S. aureus* and *Ent. faecalis* in the dilution and resazurin tests [%]^a^

Compound	Inhibition of *S. aureus* in the dilution test [%]			Inhibition of *Ent. faecalis* in the dilution test [%]		
	Concentration of the compound [μM]			Concentration of the compound [μM]		
	250 μM	125 μM	62.5 μM	250 μM	125 μM	62.5 μM
11	95.11	98.43	98.73	96.90	99.06	98.22
12	10.49	25.20	21.43	65.71	71.72	72.31
13	12.90	Inactive	Inactive	Inactive	43.04	75.52
14	55.27	54.24	31.71	73.11	61.30	45.31
15	55.62	88.42	88.63	95.83	41.67	40.92
16	100.00	99.95	99.57	85.02	76.39	22.39
18	41.89	27.59	59.49	29.73	6.26	36.82
19	Inactive	Inactive	Inactive	19.08	23.67	21.32
20	34.32	33.38	31.22	8.25	26.80	41.11
21	35.13	52.47	23.08	100.00	23.45	52.93
22	55.40	62.76	33.08	39.69	10.18	Inactive
23	Inactive	Inactive	Inactive	Inactive	18.28	47.23
Vancomycin	100.00	100.00	100.00	—	—	—
Kanamycin	—	—	—	100.00	100.00	100.00
	Inhibition of *S. aureus* in the resazurin test [%]			Inhibition of *Ent. faecalis* in the resazurin test [%]		
11	100.00	100.00	100.00	100.00	100.00	100.00
12	57.71	63.33	45.57	Inactive	Inactive	Inactive
13	Inactive	Inactive	Inactive	60.92	68.46	74.68
14	Inactive	Inactive	21.25	Inactive	18.15	39.23
15	Inactive	100.00	100.00	2.18	7.22	11.83
16	89.99	98.55	91.76	Inactive	Inactive	1.16
18	Inactive	Inactive	Inactive	Inactive	1.97	16.83
19	Inactive	Inactive	11.11	Inactive	Inactive	Inactive
20	Inactive	Inactive	19.05	Inactive	Inactive	Inactive
21	Inactive	0.22	Inactive	Inactive	Inactive	0.82
22	34.99	54.52	7.99	9.82	Inactive	Inactive
23	Inactive	Inactive	0.59	Inactive	Inactive	Inactive
Vancomycin	100.00	100.00	100.00	—	—	—
Kanamycin	—	—	—	100.00	100.00	100.00

a Inhibition of the microorganism by the tested compounds is related to the inhibition values of DMSO.

The antimicrobial activity of the compounds of this series measured by the resazurin method should represent a comparison to the dilution method (Table 1).^19,20^ The compounds were dissolved in DMSO, which indicates that DMSO was present as a co-solvent in the resazurin method as well. Therefore, the results achieved by the resazurin method might be less accurate in comparison with those from the dilution method due to the potential synergic inhibition effect of DMSO when used in higher concentrations, *i.e.*, *c* > 2.5% of DMSO in the solution. The antimicrobial effects of 16 and 22 in *S. aureus* were comparable with those of their parent triterpenoids 11 and 12, as indicated by the resazurin method, while compounds 14 and 18 showed no effect using the resazurin method. In the tests with *Ent. faecalis*, only compounds 13 and 22 showed comparable antimicrobial effects to their parent triterpenoids 11 and 12 (Table 1).

One more interesting finding was observed from the data collected in Table 1: several compounds, including the parent triterpenoids 11 and 12, showed the highest antimicrobial activity at their lowest concentration tested (*c* = 62.5 μM). This phenomenon was observed with the compounds 11, 12, 15 and 18 in the dilution tests with *S. aureus*, and was not so well pronounced in the dilution tests with *Ent. faecalis*, nor in the resazurin tests for both microorganisms, with the exception of the compound 13 in the tests with *Ent. faecalis* (Table 1; ESI,† Fig. S1). The reason for this phenomenon may be based on either the limited solubility of the parent triterpenoids and their studied derivatives in aqueous media, or their limited bioavailability in the increasing concentrations tested.

The antimicrobial activity of the studied compounds was also tested in *P. aeruginosa* and *E. coli*, both G^−^ microorganisms, by the dilution method (ESI,† Table S1). Because the compounds showed very low or even no antimicrobial activity in *P. aeruginosa* and *E. coli*, the resazurin test was not made.

### 3.3. Antiviral activity

To determine the anti-HIV-1 and anti-HSV-1 activities of the prepared derivatives of 11 and 12, the ability of the compounds to inhibit the virus-induced cytopathic effect in the MT-4 and Vero cells, respectively, was measured. In the anti-HIV-1 tests, compound 19 showed a moderate antiviral effect (EC_50_ = 57.0 ± 4.1 μM) with no cytotoxicity (CC_50_ > 100 μM) in the MT-4 cells. Two additional compounds, 20 and 23 showed antiviral effects (EC_50_ = 17.8 ± 2.1 μM and EC_50_ = 12.6 ± 0.82 μM, respectively), while being cytotoxic in the MT-4 cells (CC_50_ = 41.0 ± 5.2 μM and CC_50_ = 38.0 ± 4.2 μM, respectively). Morolic acid (12) showed a better antiviral profile than moronic acid (11; Table 2). Acyclovir or saquinavir were used as positive reference compounds (Table 2).

**Table 2:** Anti-HIV-1 activity and cytotoxicity of compounds 11–16 and 18–23 in the MT-4 cells, and anti-HSV-1 activity and cytotoxicity of compounds 11–16 and 18–23 in the Vero cells

Compound	Anti-HIV-1 activity and cytotoxicity in the MT-4 cells			Anti-HSV-1 activity and cytotoxicity in the Vero cells		
	EC_50_ [μM]	CC_50_ [μM]	SI^a^	EC_50_ [μM]	CC_50_ [μM]	SI^a^
11	6.3 ± 0.28	53.0 ± 1.7	>8.4	11.0 ± 1.1	∼26^b^	∼2.4^b^
12	6.4 ± 0.82	>100	>16	12.0 ± 0.51	35.0 ± 5.5	>2.9
13	>12	12.0 ± 0.4	<1	>60	60.0 ± 2.1	<1
14	77.0 ± 7.5	>100	>1.3	40.0 ± 2.0	>100	>2.5
15	>14	14.0 ± 0.66	<1	> 32	32.0 ± 1.5	<1
16	>81	81.0 ± 5.1	<1	38.0 ± 2.5	58.0 ± 8.8	>1.5
18	>17	17.0 ± 1.4	<1	>30	30.0 ± 1.1	<1
19	57.0 ± 4.1	>100	>1.8	58.0 ± 5.6	>100	>1.7
20	17.8 ± 2.1	41.0 ± 5.2	>2.3	34.0 ± 3.7	>100	>2.9
21	>13	13.0 ± 0.66	<1	>100	>100	<1
22	42.0 ± 4.6	∼60^b^	∼1.4^b^	27.7 ± 3.5	>100	>3.6
23	12.6 ± 0.82	38.0 ± 4.2	>2.9	30.9 ± 3.3	>100	>3.2
Saquinavir^c^	0.0015 ± 0.0005	>1	>670	—	—	—
Acyclovir^c^	—	—	—	9.4 ± 0.9	>100	>11

a SI (selectivity index) = CC_50_/EC_50_.

b It denotes approximate values only, the standard error values could not be calculated because the 95% confidence interval was too wide.

c These values were already published in the ref. 18.

In the anti-HSV-1 tests, a majority of the target compounds showed no cytotoxicity in the Vero cells. Compounds 22 (EC_50_ = 27.7 ± 3.5 μM, CC_50_ > 100 μM) and 23 (EC_50_ = 30.9 ± 3.3 μM, CC_50_ > 100 μM) showed moderate antiviral effects (Table 2), followed by several other target compounds (20, 16 and 14; Table 2). Both parent triterpenoids, moronic acid (11) and morolic acid (12) showed cytotoxicity in the Vero cells, and their antiviral profile was worse than the profile of the above mentioned target compounds (Table 2).

**Fig. 1:**
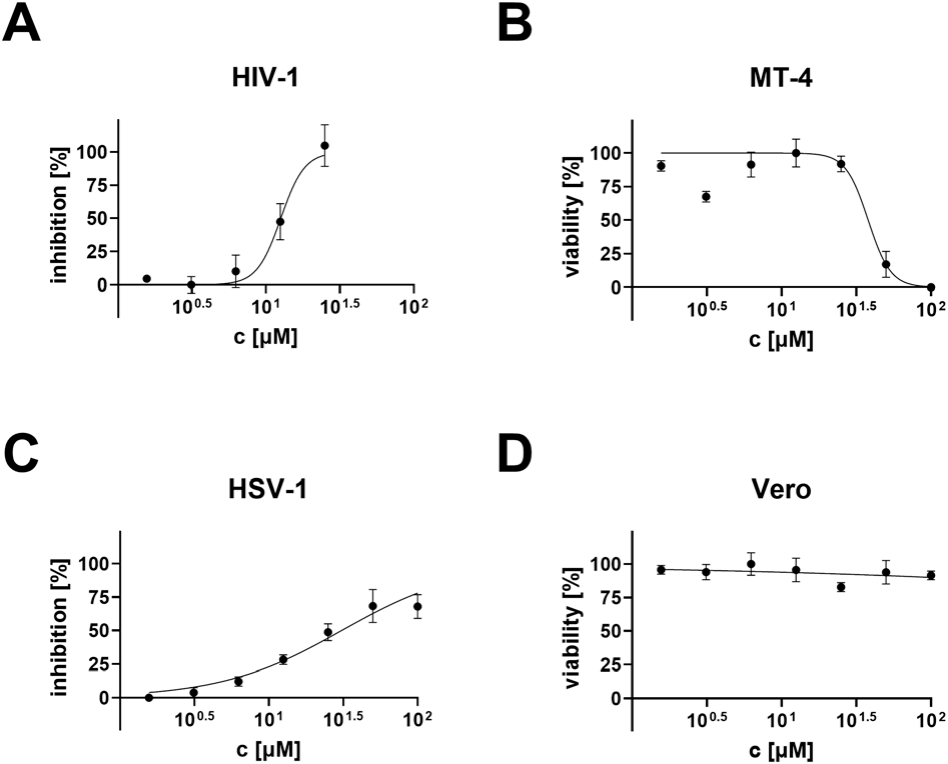
Anti-HIV-1 and anti-HSV-1 activity (A and B) and cytotoxicity (C and D) of the compound 23 in the MT-4 and Vero cells.

Compound 23 was one of a few compounds of the studied series capable of showing antiviral activity in the anti-HIV-1 and anti-HSV-1 tests (Fig. 1). While it showed cytotoxicity in the MT-4 cells, it was non-cytotoxic in the Vero cells, due to which effect its antiviral effect in the anti-HSV-1 tests showed a better profile than in the anti-HIV-1 tests (Table 2; Fig. 1). The results achieved with all other compounds of this series are shown in ESI,† Fig. S2 and S3.

### 3.4. Cytotoxicity

All target compounds showed no cytotoxicity in CCRF-CEM, MCF7, HeLa and G-361 cancer cell lines, and were not toxic in human fibroblasts. However, compound 21, one of the synthetic intermediates, showed cytotoxicity in HeLa (IC_50_ = 7.9 ± 2.1 μM; SI > 6.5), G-361 (IC_50_ = 8.0 ± 0.6 μM; SI > 6.3), MCF7 (IC_50_ = 8.6 ± 0.2 μM; SI > 6.0) and CCRF-CEM (IC_50_ = 12.0 ± 3.3 μM; SI > 4.0) cancer cell lines, whilst it was non-toxic in human fibroblasts (BJ; IC_50_ > 50 μM). Several additional synthetic intermediate compounds also showed cytotoxicity, however they were toxic in human fibroblasts as well (Table 3). CDDP, a commercially available and medicinally used agent, cisplatin, for suppressing tumours, was used as a reference. Table 3 shows that 21 displayed a better profile of cytotoxicity in MCF7 and HeLa cancer cell lines than CDDP, and no toxicity in normal cells.

**Table 3:** Cytotoxicity (IC_50_ [μM], 72 h)^a^

Compound	MW	CCRF-CEM^b^	MCF7^c^	HeLa^d^	G-361^e^	BJ^f^
11	454.70	38.3 ± 0.4	>50	35.1 ± 1.7	42.7 ± 3.3	>50
12	456.71	38.6 ± 0.3	49.4 ± 0.9	47.7 ± 1.8	>50	>50
13	742.06	13.3 ± 7.5	16.4 ± 0.3	32.3 ± 5.5	14.2 ± 1.4	11.7 ± 2.6
15	742.06	18.8 ± 5.2	14.3 ± 0.2	21.3 ± 5.4	18.4 ± 5.9	36.5 ± 2.4
18	786.12	11.7 ± 5.5	14.5 ± 2.0	16.2 ± 0.6	13.8 ± 4.3	45.1 ± 4.4
21	786.12	12.0 ± 3.3^g^	8.6 ± 0.2^h^	7.9 ± 2.1^i^	8.0 ± 0.6^j^	>50
CDDP^k^	300.05	0.8 ± 0.1	7.7 ± 1.7	11.4 ± 3.8	4.5 ± 0.6	6.9 ± 0.9

a Compounds 14, 16, 19, 20, 22 and 23 displayed no cytotoxicity in the cancer cells and no toxicity in the human fibroblasts (IC_50_ > 50 μM).

b CCRF-CEM, human T-lymphoblastic leukemia.

c MCF7, human breast adenocarcinoma.

d HeLa, human cervical cancer.

e G-361, malignant melanoma.

f BJ, normal human foreskin fibroblasts, reference cells.

g Selectivity index SI > 4.0.

h Selectivity index SI > 6.0.

i Selectivity index SI > 6.5.

j Selectivity index SI > 6.3.

k *cis*-Diaminedichloroplatinum(ii), cisplatin, a pharmacologically used agent for treating cancers, a positive reference compound.

## 4. Conclusions

A series of tripeptide derivatives of moronic acid (11) and morolic acid (12) were designed, synthesized and subjected to the screening tests for antimicrobial, antiviral and cytotoxic effects. Antimicrobial activity was tested using two G^+^ microorganisms (*S. aureus* and *Ent. faecalis*) and two G^−^ microorganisms (*P. aeruginosa* and *E. coli*). Only a few compounds of this series showed considerable antimicrobial activity that was selective towards G^+^ microorganisms. The highest inhibition of the microorganisms was achieved with 16 showing inhibition of *S. aureus* by 99.6% (*c* = 62.5 μM) and *Ent. faecalis* by 85% (*c* = 250 μM). Compound 15 displayed lower but still comparable antimicrobial effects to 16. Compound 19 showed a medium antiviral effect (EC_50_ = 57.0 ± 4.1 μM) at no cytotoxicity (CC_50_ > 100 μM) in the MT-4 cells (anti-HIV-1 tests). Compounds 20 and 23 showed anti-HIV-1 effects (EC_50_ = 17.8 ± 2.1 μM and EC_50_ = 12.6 ± 0.82 μM, respectively), while being cytotoxic in the MT-4 cells (CC_50_ = 41.0 ± 5.2 μM and CC_50_ = 38.0 ± 4.2 μM, respectively). Compounds 22 (EC_50_ = 27.7 ± 3.5 μM, CC_50_ > 100 μM) and 23 (EC_50_ = 30.9 ± 3.3 μM, CC_50_ > 100 μM) showed medium antiviral effects in the Vero cells (anti-HSV-1 tests). No target compound showed cytotoxicity in cancer cells and no toxicity in normal cells, however, the intermediate compound 21 showed cytotoxicity in MCF7 (IC_50_ = 8.6 ± 0.2 μM; SI > 6.0), G-361 (IC_50_ = 8.0 ± 0.6 μM; SI > 6.3), HeLa (IC_50_ = 7.9 ± 2.1 μM; SI > 6.5), and CCRF-CEM (IC_50_ = 12.0 ± 3.3 μM; SI > 4.0) cancer cell lines, while it was non-toxic in human fibroblasts (IC_50_ > 50 μM).

In summary, the target compound 16 (the derivative of 11) displayed antimicrobial activity against *S. aureus* and *Ent. faecalis*, and two other target compounds (19 and 22; the derivatives of 12) showed antiviral effects either in the anti-HIV-1 or in the anti-HSV-1 tests. The compounds 20, 21 and 23 (the derivatives of 12) displayed either antiviral activity or cytotoxicity. Selectivity of the effects was observed with all active compounds of this series, regardless of whether they were the target compounds or the intermediate compounds.

## 5. Experimental

### 5.1. General

The ^1^H NMR and the ^13^C NMR spectra were recorded on a Bruker AVANCE 600 MHz spectrometer at 600.13 MHz and 150.90 MHz in CDCl_3_, CD_3_OD or DMSO-*d*_6_ using tetramethylsilane (*δ* = 0.0 ppm) or the signal of the solvent as an internal reference. ^1^H NMR data are presented in the following order: chemical shift (*δ*) expressed in ppm, multiplicity (s, singlet; d, doublet; t, triplet; q, quartet; m, multiplet), coupling constants in Hertz, number of protons. For unambiguous assignment of both ^1^H and ^13^C signals 2D NMR ^1^H, ^13^C gHSQC and gHMBC spectra were measured using standard parameter sets and pulse programs delivered by the producer of the spectrometer. Infrared spectra (IR) were measured with a Nicolet iS5 FT-IR spectrometer. Mass spectra (MS) were measured with a Waters ZMD mass spectrometer in the ESI^+^ or ESI^−^ mode. A PE 2400 Series II CHNS/O Analyzer (PerkinElmer, USA) was used for simultaneous determination of C, H, and N (an accuracy of CHN determination better than 0.30% abs.). Analytical HPLC was carried out on a TSP (Thermoseparation Products, Boston, MA, USA) instrument equipped with a ConstaMetric 4100 Bio pump and a SpectroMonitor 5000 UV DAD. The analyses of the products were performed on a reverse phase Nucleosil 120-5 C18 column (250 × 4 mm; Watrex, Prague, Czech Republic) using a methanol/water mixture (9 : 1, v/v) as mobile phase at 0.5 to 1.0 mL min^−1^. The eluate was monitored at 220, 254, and 275 nm, and the UV spectra were run from 200 to 300 nm. Rapid analysis was performed on TLC silica gel plates (Merck 60F_254_) and visualization was performed by UV detection and by spraying with a methanolic solution of phosphomolybdic acid (5%) followed by heating. For column chromatography, silica gel 60 (0.063–0.200 mm) from Merck was used in combination with a mobile phase formed by chloroform/methanol (100/0 to 95/5) mixtures. Triterpenoids were purchased from Dr. Jan Šarek – Betulinines (https://www.betulinines.com), and all other chemicals and solvents were from regular commercial sources in analytical grade, and the solvents were purified by general methods before use. The analytical data of the prepared compounds are presented in the ESI.†

### 5.2. Ethyl l-methionyl-l-alanylglycinate (5)

#### Ethyl *N*-(*tert*-butoxycarbonyl)-l-alanylglycinate (2)

T3P (15.7 ml, 26.4 mmol, 5 eq.) and ethyl *N*-(*tert*-butoxycarbonyl)-l-alanine (1 g, 5.3 mmol, 1 eq.) were added to a solution of 1 (0.745 g, 5.3 mmol) in dry pyridine (25 mL), and the reaction mixture was stirred at 0 °C for 4 h. After stopping the reaction, the mixture was washed with a saturated solution of sodium bicarbonate, extracted with chloroform, and dried over Na_2_SO_4_. Evaporation of the solvent afforded a solid 2, which was used in next reaction without purification.

#### Ethyl l-alanylglycinate (3)

A solution of HCl (gas) in 1,4-dioxane (4 M, 13.2 mL, 52.85 mmol, 10 eq.) was added to the crude solid 2 (1.34 g, 5.3 mmol). The reaction mixture was stirred at 30–35 °C overnight. After stopping the reaction, the solvent was evaporated, affording a crude solid 3, which was again used in next reaction without additional purification.

#### Ethyl *N*-(*tert*-butoxycarbonyl)-l-methionyl-l-alanylglycinate (4)

T3P (15.7 mL, 26.4 mmol, 5 eq.) and *N*-(*tert*-butoxycarbonyl)-l-methionine (1.3 g, 5.3 mmol, 1 eq.) were added to a solution of the crude solid 3 (1 g, 5.3 mmol) in dry pyridine (25 mL), and the reaction mixture was stirred at r.t. for 5 h. After stopping the reaction, the mixture was washed with a saturated solution of sodium bicarbonate, extracted with chloroform, and dried over Na_2_SO_4_. Evaporation of the solvent afforded a crude solid 4, which was used in next reaction without additional purification.

#### Ethyl l-methionyl-l-alanylglycinate (5)

A solution of HCl (gas) in 1,4-dioxane (4 M, 13.2 mL, 52.85 mmol, 10 eq.) was added to the crude solid 4 (1.45 g, 5.3 mmol). The reaction mixture was stirred at 30–35 °C overnight. After stopping the reaction, the solvent was evaporated, affording a crude solid that was purified by column chromatography, yielding finally 0.9 g (56% total yield of the 4-step procedure) of the product 5.

### 5.3. Ethyl glycyl-l-alanyl-l-methioninate (10)

#### Ethyl *N*-(*tert*-butoxycarbonyl)-l-alanyl-l-methioninate (7)

T3P (15.7 mL, 26.4 mmol, 5 eq.) and *N*-(*tert*-butoxycarbonyl)-l-alanine (1 g, 5.3 mmol, 1 eq.) were added to a solution of 6 (1.3 g, 5.3 mmol) in dry pyridine (25 mL), and the reaction mixture was stirred at r.t. overnight. After stopping the reaction, the mixture was washed with a saturated solution of sodium bicarbonate, extracted with chloroform, and dried over Na_2_SO_4_. Evaporation of the solvent afforded a crude solid 7, which was used in the next reaction without additional purification.

#### Ethyl l-alanyl-l-methioninate (8)

A solution of HCl (gas) in 1,4-dioxane (4 M, 13.2 mL, 52.85 mmol, 10 eq.) was added to the crude solid 7 (5.3 mmol), and the reaction mixture was stirred at 30–35 °C overnight. After stopping the reaction, the solvent was evaporated, affording a crude solid 8, which was used in the next reaction without additional purification.

#### Ethyl *N*-(*tert*-butoxycarbonyl)glycyl-l-alanyl-l-methioninate (9)

T3P (15.7 mL, 26.4 mmol, 5 eq.) and *N*-(*tert*-butoxycarbonyl)glycine (1.3 g, 5.3 mmol, 1 eq.) were added to a solution of the crude solid 8 (5.3 mmol) in dry pyridine (25 mL), and the reaction mixture was stirred at r.t. overnight. After stopping the reaction, the mixture was washed with a saturated solution of sodium bicarbonate, extracted with chloroform, and dried over Na_2_SO_4_. Evaporation of the solvent afforded a crude solid 9, which was used in the next reaction without additional purification.

#### Ethyl glycyl-l-alanyl-l-methioninate (10)

A solution of HCl in 1,4-dioxane (4 M, 13.2 mL, 52.85 mmol, 10 eq.) was added to the crude solid 9 (5.3 mmol), and the reaction mixture was stirred at 30–35 °C overnight. After stopping the reaction, the mixture was filtered, affording crude crystals that were washed with ether, yielding finally 1.5 g (83% total yield of the 4-step procedure) of the product 10.

### 5.4. Ethyl *N*-(3,28-dioxoolean-18-*en*-28-yl)glycyl-l-alanyl-l-methioninate (13)

A solution of oxalyl chloride in DCM (2 M, 1.6 mL, 3.2 mmol, 7 eq.) was added to a solution of 11 (208 mg, 0.46 mmol) in dry DCM (8 mL), and the reaction mixture was stirred at r.t. for 3.5 h. Then the reaction mixture was evaporated, and the residue was re-dissolved in dry DCM (8 mL), and 10 (168 mg, 0.55 mmol, 1.2 eq.) and DIPEA (207 μL, 1.2 mmol, 2.6 eq.) were added. The reaction mixture was stirred at r.t. overnight. After stopping the reaction, the resulting mixture was evaporated, affording a crude solid 13 that was used in the next reaction without additional purification. However, a sample of 13 was purified for the spectral analysis and biological screening tests.

### 5.5. *N*-(3,28-Dioxoolean-18-en-28-yl)glycyl-l-alanyl-l-methionine (14)

LiOH·H_2_O (66 mg, 1.57 mmol, 3.4 eq.) was added to a solution of the crude solid 13 (0.46 mmol) in MeOH (20 mL), and the reaction mixture was stirred at r.t. for 1 h. After stopping the reaction, the solvent was evaporated, the residue was dissolved in chloroform, washed with water, and dried over Na_2_SO_4_. Evaporation of the solvent afforded a solid, which was purified by column chromatography, yielding finally 160 mg (49%) of the product 14.

### 5.6. Ethyl *N*-(3,28-dioxoolean-18-*en*-28-yl)-l-methionyl-l-alanylglycinate (15)

A solution of oxalyl chloride in DCM (2 M, 0.9 mL, 1.8 mmol, 8 eq.) was added to a solution of 11 (104 mg, 0.229 mmol) in dry DCM (4 mL), and the reaction mixture was stirred at r.t. for 3 h. The reaction mixture was evaporated, and the residue was re-dissolved in dry DCM (4 mL). Then DIPEA (103 μL, 0.6 mmol, 2.6 eq.) and 5 (102 mg, 0.33 mmol, 1 eq.) were added, and the reaction mixture was stirred at r.t. overnight. After stopping the reaction, the resulting mixture was evaporated, affording a crude solid 15 that was used in the next reaction without additional purification. However, a sample of 15 was purified for the spectral analysis and biological screening tests.

### 5.7. *N*-(3,28-Dioxoolean-18-*en*-28-yl)-l-methionyl-l-alanylglycine (16)

LiOH·H_2_O (52 mg, 1.24 mmol, 5.4 eq.) was added to a solution of the crude solid 15 (0.23 mmol) in MeOH (10 mL), and the reaction mixture was stirred at r.t. for 2 h. After stopping the reaction, the mixture was evaporated, affording a solid that was purified by column chromatography, affording 100 mg (61%) of the product 16.

### 5.8. (3β)-3-(Acetyloxy)olean-18-*en*-28-oic acid (17)

Acetic anhydride (179 μL, 1.9 mmol, 1.44 eq.), DMAP (21 mg, 0.17 mmol, 0.13 eq.) and triethylamine (0.6 mL, 4.32 mmol, 3.3 eq.) were added to a solution of 12 (0.6 g, 1.3 mmol) in dry THF (10 mL). The reaction mixture was heated to boiling for 3 h. After stopping the reaction, water was added, and the mixture was stirred at r.t. overnight. The resulting mixture was extracted with chloroform, and the extract was dried over Na_2_SO_4_. Evaporation of the solvent afforded a crude solid 17, which was used in the next reaction without additional purification. However, a sample of 17 was purified for the spectral analysis.

### 5.9. Ethyl (3β)-*N*-[3-(acetyloxy)-28-oxoolean-18-*en*-28-yl]glycyl-l-alanyl-l-methioninate (18)

A solution of oxalyl chloride in DCM (2 M, 2 mL, 4 mmol, 7 eq.) was added to a solution of the crude solid 17 (284 mg, 0.57 mmol) in dry DCM (10 mL), and the reaction mixture was stirred at r.t. for 2.5 h. Then the solvent was evaporated, the residue was re-dissolved in dry DCM (10 mL), and 10 (192 mg, 0.63 mmol, 1.1 eq.) and DIPEA (192 μL, 1.5 mmol, 2.6 eq.) were added. The reaction mixture was stirred at r.t. overnight. After stopping the reaction, the solvent was evaporated, affording a solid that was purified by column chromatography, yielding finally 303 mg (68%) of the product 18.

### 5.10. (3β)-*N*-(3-Hydroxy-28-oxoolean-18-*en*-28-yl)glycyl-l-alanyl-l-methionine (19)

LiOH·H_2_O (12 mg, 0.28 mmol, 1.5 eq.) was added to a solution of 18 (148 mg, 0.19 mmol) in MeOH (10 mL), and the reaction mixture was stirred at r.t. for 1 h. The solvent was then evaporated, the residue was re-dissolved in MeOH (10 mL), and an additional quantity of LiOH·H_2_O (24 mg, 0.57 mmol, 3 eq.) was added. The reaction mixture was heated to boiling for 3 h. After stopping the reaction, the solvent was evaporated, affording a solid that was purified by column chromatography, yielding finally 123 mg (92%) of the product 19.

### 5.11. (3β)-*N*-[3-(Acetyloxy)-28-oxoolean-18-*en*-28-yl]glycyl-l-alanyl-l-methionine (20)

LiOH·H_2_O (8 mg, 0.19 mmol, 1 eq.) was added to a solution of 18 (149 mg, 0.19 mmol) in MeOH (10 mL), and the reaction mixture was heated to boiling for 2 h. After stopping the reaction, the mixture was evaporated, affording a solid that was purified by column chromatography, yielding 90 mg (63%) of the product 20.

### 5.12. Ethyl (3β)-*N*-[3-(acetyloxy)-28-oxoolean-18-en-28-yl]-l-methionyl-l-alanylglycinate (21)

A solution of oxalyl chloride in DCM (2 M, 2 mL, 4 mmol, 7 eq.) was added to a solution of 17 (282 mg, 0.566 mmol) in dry DCM (10 mL), and the reaction mixture was stirred at r.t. for 4 h. The solvent was evaporated, the residue was re-dissolved in dry DCM (10 mL), and then DIPEA (256 μL, 1.5 mmol, 2.6 eq.) and 5 (195 mg, 0.64 mmol, 1.1 eq.) were added. The reaction mixture was stirred at r.t. overnight. After stopping the reaction, the solvent was evaporated, affording a solid that was purified by column chromatography, yielding finally 288 mg (76%) of the product 21.

### 5.13. (3β)-*N*-(3-Hydroxy-28-oxoolean-18-*en*-28-yl)-l-methionyl-l-alanylglycine (22)

LiOH·H_2_O (11 mg, 0.27 mmol, 1.5 eq.) was added to a solution of 21 (0.18 mmol) in MeOH (10 mL), and the reaction mixture was stirred at r.t. for 1 h. The solvent was evaporated, the residue was re-dissolved in MeOH (10 mL), and then an additional quantity of LiOH·H_2_O (49 mg, 1.17 mmol, 6.5 eq.) was added. The reaction mixture was heated to boiling for 2 h. After stopping the reaction, the solvent was evaporated, affording a solid that was purified by column chromatography, yielding finally 120 mg (95%) of the product 22.

### 5.14. (3β)-*N*-[3-(Acetyloxy)-28-oxoolean-18-*en*-28-yl]-l-methionyl-l-alanylglycine (23)

LiOH·H_2_O (8 mg, 0.19 mmol, 1.05 eq.) was added to a solution of 21 (142 mg, 0.18 mmol) in MeOH (10 mL), and the reaction mixture was stirred at r.t. for 1.5 h. After stopping the reaction, the solvent was evaporated, affording a solid that was purified by column chromatography, yielding finally 99 mg (72%) of the product 23.

### 5.15. Antimicrobial activity

#### Dilution method

A 96-well microtitration plate was used for performing antimicrobial testing, because it enabled the measuring of several samples with different concentrations simultaneously.^20^ In the given experiments, concentrations of the studied compounds, *c* = 250 μM, 125 μM and 62.5 μM, were made by a sequential dilution. The total volume of each well was set to 200 μL. The determination of the antimicrobial activity of each sample was performed in a triplicate. The microtitration plate also involved the wells containing the solutions of DMSO (*c* = 2.5%, 1.25% and 0.625%) in MHB, used as the negative controls, and wells containing MHB (100 μL) and a solution of the antibiotics (5 μL; *c* = 10 mg mL^−1^) to get the final concentration in the well *c* = 0.25 mg mL^−1^. Finally, the diluted solution of the microbial culture (0.5 McF; corresponding to 5 × 10^5^ CFU mL^−1^) was added to all wells except for those used for measuring the background. Subsequently, the microtitration plate was analysed using a Synergy spectrophotometer for 24 h. The values of changing absorbance were recorded at 625 nm wavelength in hourly intervals. The temperature applied in the analysis was species-dependent, set either to 35 °C (for *S. aureus*, *E. coli* and *P. aeruginosa*) or to 28 °C (for *Ent. faecalis*). After the analysis was completed, the data were used for drawing the growth curves, giving information on bacteriostatic effects of the studied compounds. The growth inhibition (*I* [%]) was calculated on the basis of the growth curves taking the absorbance values obtained in hour 1 and in hour 18 of the growth curves (eqn (1)):1
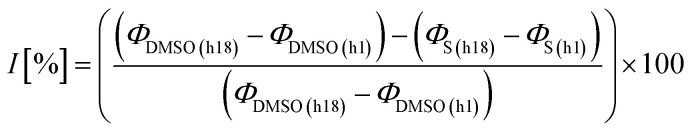
where *Φ*_DMSO(h1)_ and *Φ*_DMSO(h18)_ are the average values of the measured absorbance at a 625 nm wavelength in the presence of DMSO (*c* = 2.5%, 1.25% and 0.625%) in hour 1 and hour 18 of the growth curves, respectively; *Φ*_S(h1)_ and *Φ*_S(h18)_ are the average values of the measured absorbance at a 625 nm wavelength of the sample without DMSO in hour 1 and hour 18 of the growth curves, respectively.

#### Cell viability

Cell viability was measured by the resazurin test, using the colour effect of the changing resazurin to resorufin.^19^ After 24 hours of cultivation of the solutions in a 96-well microtitration plate used in the dilution test, a part of the suspension (100 μL) was transferred into a new microtitration plate. A blue solution of resazurin (100 μL; final concentration *c* = 0.025 mg mL^−1^; 200-times dilution of the store solution of resazurin, *c* = 5 mg mL^−1^) was added to each well. Incubation in the wells of the microtitration plate was made with a continuous shaking of the plate. The colour change (the blue resazurin to the pink resorufin) was visible after 30 min. The accompanying fluorescence effect was measured using a Synergy spectrophotometer at *λ*_excitation_ = 560 nm and at *λ*_emission_ = 590 nm wavelengths. Evaluating the data of the described method, bactericide effects of the studied compounds were determined, *i.e.*, a percentage of inhibition of the given microorganism by the studied compound was calculated based on the fluorescence data (eqn (2)):2
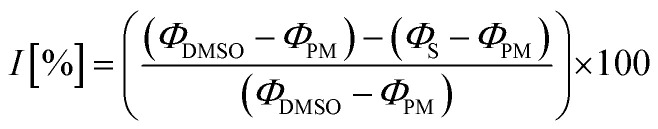
where *Φ*_DMSO_ is the average value of the measured fluorescence in the presence of DMSO (*c* = 1%); *Φ*_PM_ is the average value of the measured fluorescence of the pure medium (MHB); *Φ*_S_ is the average value of the measured fluorescence of the sample with DMSO.

### 5.16. Antiviral activity

The anti-HIV-1 and anti-HSV-1 activity and cytotoxicity in the MT-4 cells or Vero cells, respectively, were determined as recently described.^18^ The Tempest liquid dispenser system (Formulatrix) was used for all dispensing steps.

### 5.17. Cytotoxicity screening tests

The cytotoxicity screening tests were performed according to the standard experimental procedure published earlier.^15^ Cell viability was measured in CCRF-CEM, human T-lymphoblastic leukemia; MCF7, human breast adenocarcinoma; HeLa, human cervical cancer; G-361, malignant melanoma or BJ, normal human foreskin fibroblasts after 72 h using resazurin (Sigma-Aldrich).

## Abbreviations

BJ: Normal human foreskin fibroblast
CCRF-CEM: Human T-lymphoblastic leukemia
DCM: Dichloromethane
DIPEA: Diisopropylethylamine
G-361: Malignant melanoma
HeLa: Human cervical cancer
GAM: Tripeptide Gly–Ala–Met
GAMVVH: Hexapeptide Gly–Ala–Met–Val–Val–His
MAG: Tripeptide Met–Ala–Gly
MAGVDHI: Heptapeptide Met–Ala–Gly–Val–Asp–His–Ile
MCF7: Human breast adenocarcinoma
r.t.: Room temperature
T3P: 1-Propanephosphonic acid anhydride

## Data availability

Data for this article are available in the main text of the manuscript and in the ESI† file submitted together with the main text of the manuscript. Data is also available from the corresponding author of the manuscript, Prof. Zdenek Wimmer, zdenek.wimmer@vscht.cz; wimmer@biomed.cas.cz.

## Author contributions

Conceptualization: Z. W., J. W. and P. L.; methodology: L. C., U. B., M. K., L. R., J. T. and D. Š.; investigation: L. C., U. B., M. K., L. R., J. T. and D. Š.; resources: Z. W.; data curation: L. C., U. B., L. R., J. T. and D. Š.; writing – original draft preparation: Z. W., J. W., L. R. and P. L.; writing – review and editing: Z. W., J. W., L. R. and P. L.; project administration: Z. W.; funding acquisition: Z. W. and J. W. All authors have read and agreed to the published version of the manuscript.

## Conflicts of interest

There are no conflicts to declare.

## Supplementary Material

MD-OLF-D4MD00742E-s001
