# Supplementary material for: Amides of moronic acid and morolic acid with the tripeptides MAG and GAM targeting antimicrobial, antiviral and cytotoxic effects
Source: RSC Med Chem. 2024 Oct 29;16(2):801–11. doi: 10.1039/d4md00742e (PMC11575580; doi:10.1039/d4md00742e)

Supplementary Information (SI) for RSC Medicinal Chemistry.
This journal is © The Royal Society of Chemistry 2024

Electronic Supplementary Information (ESI)

Amides of moronic acid and morolic acid with the tripeptides MAG and GAM targeting antimicrobial, antiviral and cytotoxic effects

Uladzimir Bildziukevich,[a] Lucie Černá,[b] Jana Trylčová,[d] Marie Kvasnicová,[e,f] Lucie Rárová,[e,f] David Šaman,[d] Petra Lovecká,[b] Jan Weber [d] and Zdeněk Wimmer *,[a,c]

[a] Institute of Experimental Botany of the Czech Academy of Sciences, Isotope Laboratory, Vídeňská 1083, 14220 Prague 4, Czech Republic;

[b] Department of Biochemistry and Microbiology, University of Chemistry and Technology in Prague, Technická 5, 16628 Prague 6, Czech Republic;

[c] Department of Chemistry of Natural Compounds, University of Chemistry and Technology in Prague, Technická 5, 16628 Prague 6, Czech Republic;

[d] Institute of Organic Chemistry and Biochemistry of the Czech Academy of Sciences, Flemingovo náměstí 2, 16610 Prague 6, Czech Republic;

[e] Laboratory of Growth Regulators, Faculty of Science, Palacký University, and Institute of Experimental Botany of the Czech Academy of Sciences, Šlechtitelů 27, CZ-77900 Olomouc, Czech Republic;

[f] Department of Experimental Biology, Faculty of Science, Palacký University, Šlechtitelů 27, CZ-77900 Olomouc, Czech Republic.

Content

1. **NMR spectra** of the prepared compounds. **p. 3**
2. **Figure S1.** Graphical image of the inhibition effect of **16** on *Staphylococcus aureus* and *Enterococcus faecalis* in the dilution test. **p. 30**
3. **Table S1.** Inhibition effect of the tested compounds on *Pseudomonas aeruginosa* and *Escherichia coli* in the dilution test. **p. 31**
4. **Figures S2 and S3.** Antiviral activity and cytotoxicity of the studied compounds. **p. 32**

1. NMR spectra of the prepared compounds

Ethyl L-methionyl-L-alanylglycinate (**5**)

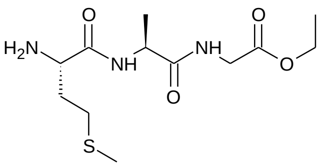

^{1}H NMR (CD_3OD): δ [ppm] 1.26 (3H, t, *J*=7.1 Hz, H12), 1.39 (3H, d, *J*=7.1 Hz, H5), 1.78-1.85 (1H, m, H8), 1.97-2.03 (1H, m, H8), 2.09 (3H, s, H10), 2.55-2.60 (2H, m, H9), 3.49 (1H, dd, J_1=5.8 Hz, J_2=7.4 Hz, H7), 3.88 (1H, d, *J*=17.6 Hz, H2), 3.98 (1H, d, *J*=17.6 Hz, H2), 4.18 (2H, q, *J*=7.1 Hz, H11), 4.43 (1H, q, *J*=7.1 Hz, H4). ^{13}C NMR (CD_3OD): δ [ppm] 14.5 (q, C12), 15.1 (q, C10), 18.2 (q, C5), 31.0 (t, C9), 35.4 (t, C8), 42.0 (t, C2), 50.1 (d, C4), 55.1 (d, C7), 62.3 (t, C11), 171.1 (s, C1), 175.3 (s, C3), 176.5 (s, C6). IR [cm^{-1}]: 3383, 2943, 2868, 1522, 1419, 1366, 1249, 1168, 882, 755. MS: *m/z* = 306.2 $[M+H]^+$, 328.1 $[M+Na]^+$, 344 $[M+K]^+$ (ESI^+, coin voltage 20 V). For $C_{12}H_{23}N_3O_4S$ (305.39) calcd. C 47.19, H 7.59, N 13.76, S 10.50, found C 47.22, H 7.61, N 13.74, S 10.47.

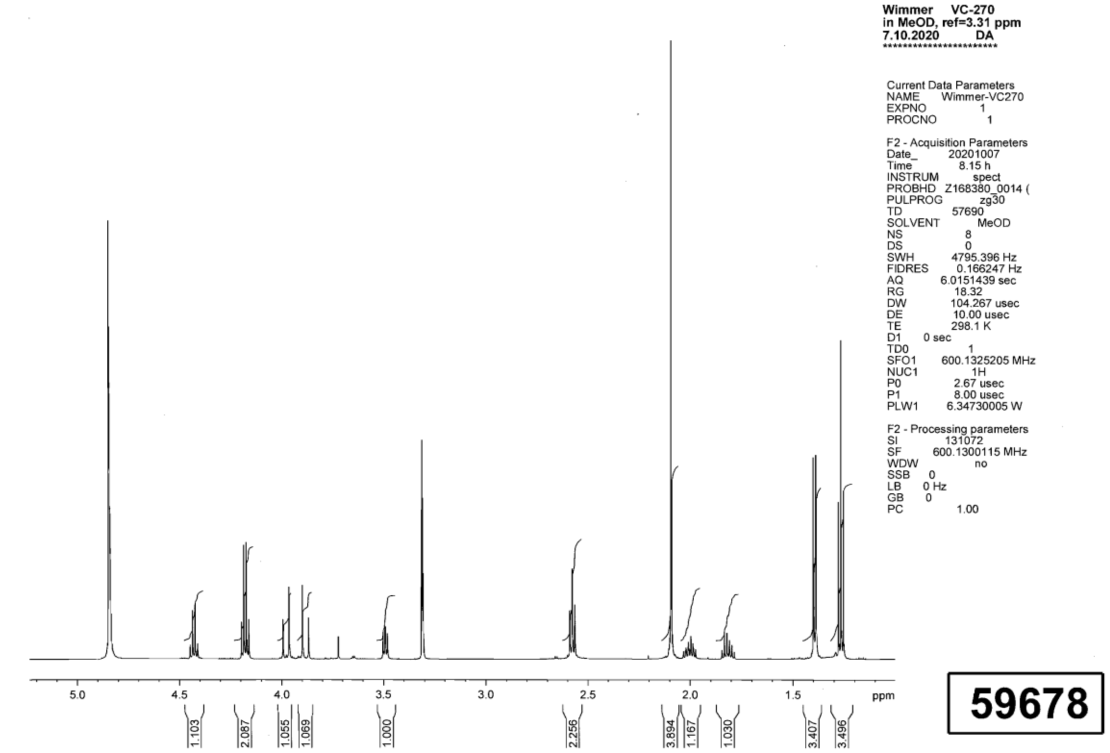
Wimmer VC-270
in MeOD, ref=3.31 ppm
7.10.2020 DA
Current Data Parameters
NAME Wimmer-VC270
EXPNO 1
PROCNO 1
F2 - Acquisition Parameters
Date_ 20201007
Time 8.15 h
INSTRUM spect
PROBHD Z168380_0014 (
PULPROG zg30
TD 57690
SOLVENT MeOD
NS 8
DS 0
SWH 4795.396 Hz
FIDRES 0.166247 Hz
AQ 6.0151439 sec
RG 18.32
DW 104.267 usec
DE 10.00 usec
TE 298.1 K
D1 0 sec
TD0 1
SFO1 600.1325205 MHz
NUC1 1H
P0 2.67 usec
P1 8.00 usec
PLW1 6.34730005 W
F2 - Processing parameters
SI 131072
SF 600.1300115 MHz
WDW no
SSB 0
LB 0 Hz
GB 0
PC 1.00
5.0
4.5
4.0
3.5
3.0
2.5
2.0
1.5
ppm
1.103
2.087
1.055
1.069
1.000
2.256
3.894
1.167
1.030
3.407
3.496
59678

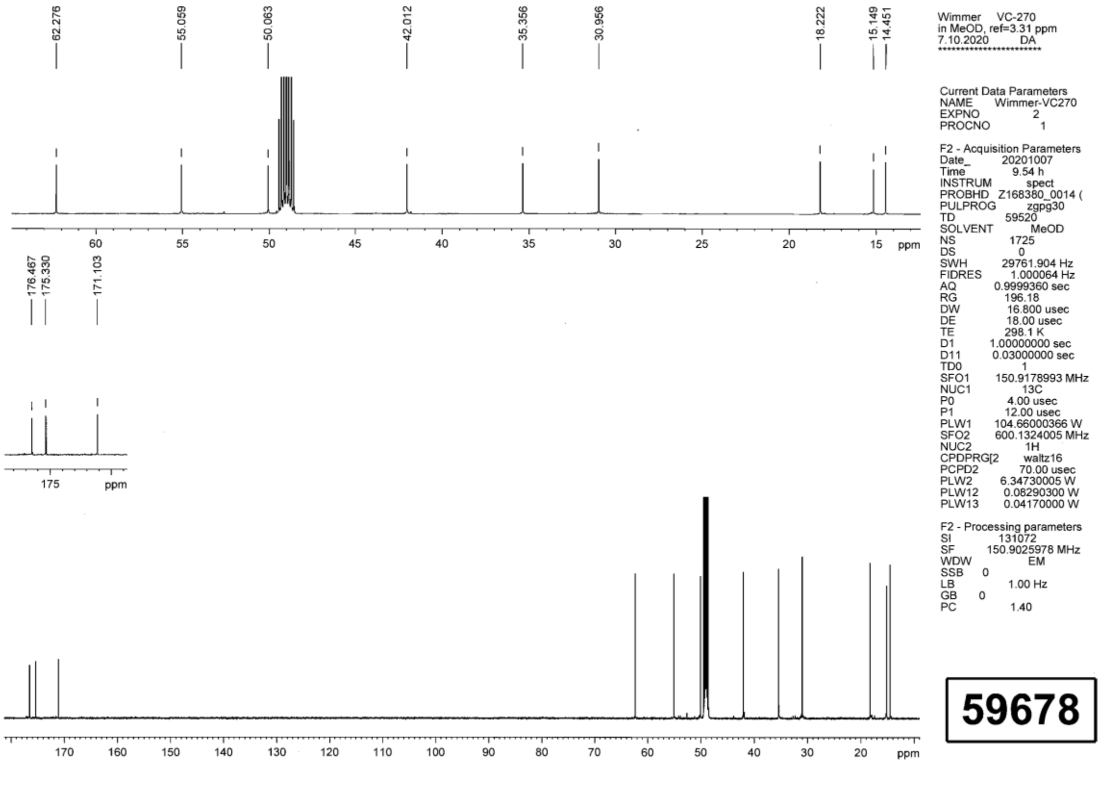
62.276
55.059
50.063
42.012
35.356
30.956
18.222
15.149
14.451
60
55
50
45
40
35
30
25
20
15
ppm
176.467
175.330
171.103
175
ppm
Wimmer VC-270
in MeOD, ref=3.31 ppm
7.10.2020 DA
Current Data Parameters
NAME Wimmer-VC270
EXPNO 2
PROCNO 1
F2 - Acquisition Parameters
Date_ 20201007
Time 9.54 h
INSTRUM spect
PROBHD Z168380_0014 (
PULPROG zgpg30
TD 59520
SOLVENT MeOD
NS 1725
DS 0
SWH 29761.904 Hz
FIDRES 1.000064 Hz
AQ 0.9999360 sec
RG 196.18
DW 16.800 usec
DE 18.00 usec
TE 298.1 K
D1 1.00000000 sec
D11 0.03000000 sec
TD0 1
SFO1 150.9178993 MHz
NUC1 13C
P0 4.00 usec
P1 12.00 usec
PLW1 104.66000366 W
SFO2 600.1324005 MHz
NUC2 1H
CPDPRG[2 waltz16
PCPD2 70.00 usec
PLW2 6.34730005 W
PLW12 0.08290300 W
PLW13 0.04170000 W
F2 - Processing parameters
SI 131072
SF 150.9025978 MHz
WDW EM
SSB 0
LB 1.00 Hz
GB 0
PC 1.40
59678
170
160
150
140
130
120
110
100
90
80
70
60
50
40
30
20
ppm

Ethyl glycyl-L-alanyl-L-methioninate (**10**)

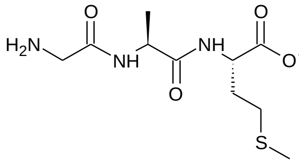

^{1}H NMR (DMSO-d_6): δ [ppm] 0.80 (3H, t, *J*=7.1 Hz, H12), 0.94 (1.5H, d, *J*=7.1 Hz, H8), 0.95 (1.5H, d, *J*=7.2 Hz, H8), 1.60-1.75 (2H, m, H3), 1.67 (3H, s, H5), 2.11-2.25 (2H, m, H4), 3.46 (1H, d, *J*=15.8 Hz, H10), 3.50 (1H, d, *J*=15.8 Hz, H10), 3.67-3.76 (2H, m, H11), 4.04 (0.5H, m, H7), 4.08 (0.5H, m, H7), 4.06-4.11 (1H, m, H2), 8.15 (1H, d, *J*=7.6 Hz, NH-7), 8.45 (2H, bs, NH_2-10), 8.55 (0.5H, d, *J*=7.4 Hz, NH-2), 8.62 (0.5H, d, *J*=7.4 Hz, NH-2). ^{13}C NMR (DMSO-d_6): δ [ppm] 18.61 (q, C12), 19.22 (q, C5), 19.26 (q, C5), 22.81 (q, C8), 22.95 (q, C8), 34.88 (t, C4), 34.95 (t, C4), 35.63 (t, C3), 45.83 (t, C10), 45.93 (t, C10), 53.89 (d, C2), 54.28 (d, C2), 56.38 (d, C7), 56.48 (d, C7), 65.56 (t, C11), 170.86 (s, C9), 170.99 (s, C9), 176.72 (s, C1), 176.77 (s, C1), 177.33 (s, C6), 177.37 (s, C6). IR [cm^{-1}]: 3384, 2944, 2869, 1420, 1365, 1250, 1168, 754. MS: *m/z* = 306.0 [M+H]$^{+}$ (ESI^{+}, coin voltage 10 V), *m/z* = 339.9 [M+Cl]$^{-}$ (ESI^{-}, coin voltage 10 V). For $C_{12}H_{23}N_3O_4S$ (305.39) calcd. C 47.19, H 7.59, N 13.76, S 10.50, found C 47.17, H 7.62, N 13.79, S 10.52.

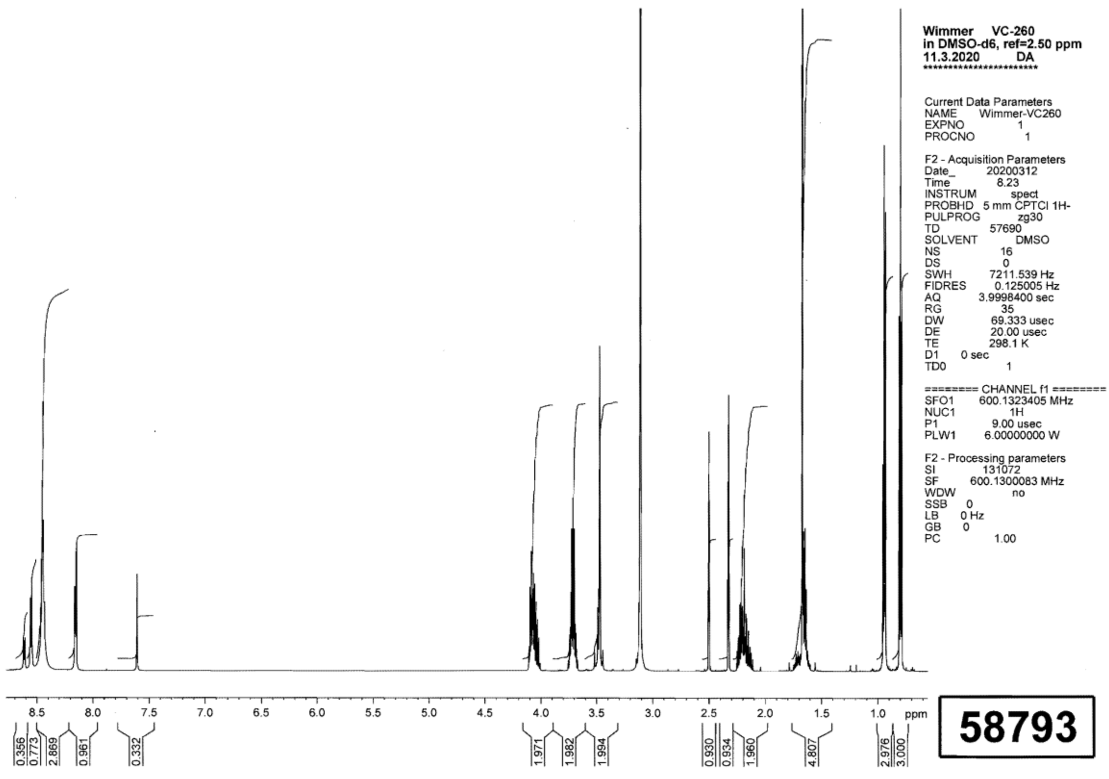

Wimmer VC-260
in DMSO-d6, ref=2.50 ppm
11.3.2020 DA
****

Current Data Parameters
NAME Wimmer-VC260
EXPNO 1
PROCNO 1

F2 - Acquisition Parameters
Date_ 20200312
Time 8.23
INSTRUM spect
PROBHD 5 mm CPTCI 1H-
PULPROG zg30
TD 57690
SOLVENT DMSO
NS 16
DS 0
SWH 7211.539 Hz
FIDRES 0.125005 Hz
AQ 3.9998400 sec
RG 35
DW 69.333 usec
DE 20.00 usec
TE 298.1 K
D1 0 sec
TD0 1

======== CHANNEL f1 ========
SFO1 600.1323405 MHz
NUC1 1H
P1 9.00 usec
PLW1 6.00000000 W

F2 - Processing parameters
SI 131072
SF 600.1300083 MHz
WDW no
SSB 0
LB 0 Hz
GB 0
PC 1.00

58793

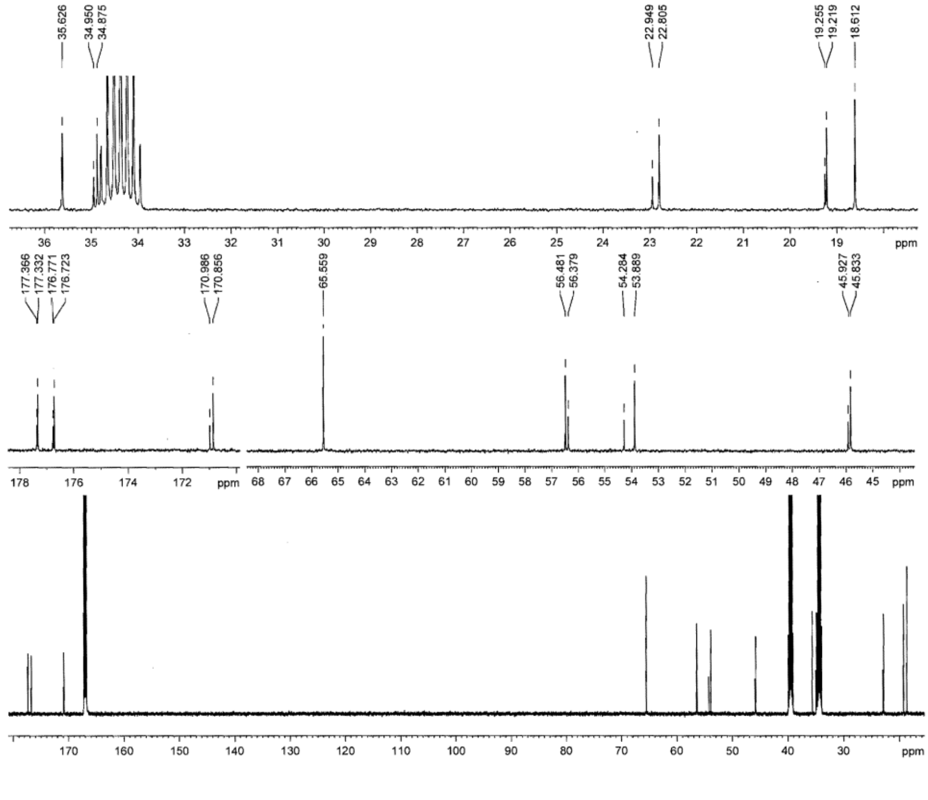

Wimmer VC-260
in DMSO-d6, ref=2.50 ppm
11.3.2020 DA
****

Current Data Parameters
NAME Wimmer-VC260
EXPNO 2
PROCNO 1

F2 - Acquisition Parameters
Date_ 20200312
Time 8.28
INSTRUM spect
PROBHD 5 mm CPTCI 1H-
PULPROG zgpg30
TD 59520
SOLVENT DMSO
NS 256
DS 0
SWH 29761.904 Hz
FIDRES 0.500032 Hz
AQ 0.9999360 sec
RG 196.18
DW 16.800 usec
DE 18.00 usec
TE 298.1 K
D1 1.00000000 sec
D11 0.03000000 sec
TD0 1

======== CHANNEL f1 ========
SFO1 150.9178993 MHz
NUC1 13C
P1 12.00 usec
PLW1 80.00000000 W

======== CHANNEL f2 ========
SFO2 600.1324005 MHz
NUC2 1H
CPDPRG[2 waltz16
PCPD2 70.00 usec
PLW2 6.00000000 W
PLW12 0.09918400 W
PLW13 0.04860000 W

F2 - Processing parameters
SI 131072
SF 150.9020136 MHz
WDW EM
SSB 0
LB 1.00 Hz
GB 0

58793

Ethyl *N*-(3,28-dioxoolean-18-en-28-yl)glycyl-L-alanyl-L-methioninate (**13**)

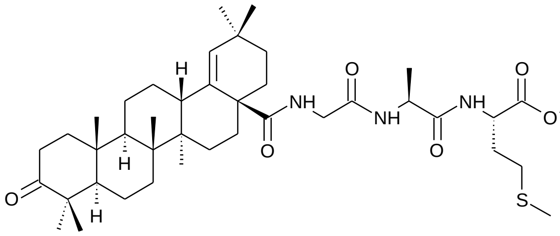

^{1}H NMR (CDCl$_3$): δ [ppm] 0.75 (s, H27), 0.92 (d, *J*=0.5 Hz, H25), 0.95 (s, H26), 0.96 (s, H30), 0.99 (s, H24), 1.02 (s, H29), 1.05 (s, H23), 1.18 (2H, dt, J_1=3.2 Hz, J_2=3.2 Hz, J_3=13.1 Hz, H15), 1.26 (3H, t, *J*=7.1 Hz, H12'), 1.37 (3H, d, *J*=7.2 Hz, H5'), 1.82 (2H, ddd, J_1=3.5 Hz, J_2=4.9 Hz, J_3=13.9 Hz, H2), 1.91-2.00 (1H, m, H8'), 2.06 (3H, s, H10'), 2.10-2.17 (1H, m, H8'), 2.15 (1H, ddd, J_1=1.7 Hz, J_2=3.4 Hz, J_3=11.8 Hz, H13), 2.38 (2H, dt, J_1=3.4 Hz, J_2=3.4 Hz, J_3=13.3 Hz, H21), 2.42 (1H, ddd, J_1=5.0 Hz, J_2=8.3 Hz, J_3=15.8 Hz, H7), 2.45-2.50 (2H, m, H9'), 2.47 (1H, ddd, J_1=7.8 Hz, J_2=9.0 Hz, J_3=15.8 Hz, H7), 3.89 (1H, dd, J_1=5.4 Hz, J_2=16.4 Hz, H1'), 3.94 (1H, dd, J_1=5.2 Hz, J_2=16.4 Hz, H1'), 4.18 (2H, dq, J_1=0.8 Hz, J_2=7.1 Hz, J_3=7.1 Hz, J_4=7.1 Hz, H11'), 4.45 (1H, m, H3'), 4.62 (1H, dt, J_1=5.1 Hz, J_2=7.6 Hz, J_3=7.6 Hz, H6'), 5.35 (2H, dd, J_1=0.5 Hz, J_2=1.7 Hz, H19). ^{13}C NMR (CDCl$_3$): δ [ppm] 14.1 (q, C12'), 14.8 (q, C27), 15.5 (q, C10'), 15.9 (q, C26), 16.6 (q, C25), 18.2 (q, C5'), 19.6 (t, C6), 20.9 (q, C24), 21.5 (t, C11), 26.2 (t, C12), 26.9 (q, C23), 28.9 (q, C30), 29.3 (t, C15), 29.9 (t, C9'), 30.8 (q, C29), 31.4 (t, C8'), 32.3 (s, C20), 32.7 (t, C21), 33.2 (d, C16), 33.7 (t, C22), 33.9 (t, C2), 34.0 (t, C7), 36.9 (s, C10), 39.8 (t, C1), 40.6 (s, C8), 41.7 (d, C13), 42.6 (s, C14), 43.5 (t, C1'), 47.2 (s, C4), 48.2 (s, C17), 50.4 (d, C9), 51.7 (d, C6'), 54.8 (d, C5), 61.7 (t, C11'), 99.0 (d, C3'), 136.1 (d, C19), 137.8 (s, C18), 168.7 (s, C2'), 176.6 (s, C28), 171.2 (s, C4'), 171.5 (s, C7'), 218.2 (s, C3). IR [cm^{-1}]: 3387, 2936, 2869, 1698, 1520, 1420, 1367, 1249, 1168. MS: *m/z* = 742.5 [M+H]$^+$ (ESI$^+$, coin voltage 10 V), *m/z* = 740.4 [M–H]$^-$ (ESI$^-$, coin voltage 10 V). For $C_{42}H_{67}N_3O_6S$ (742.06) calcd. C 67.98, H 9.10, N 5.66, S 4.32, found C 68.01, H 9.08, N 5.69, S 4.35.

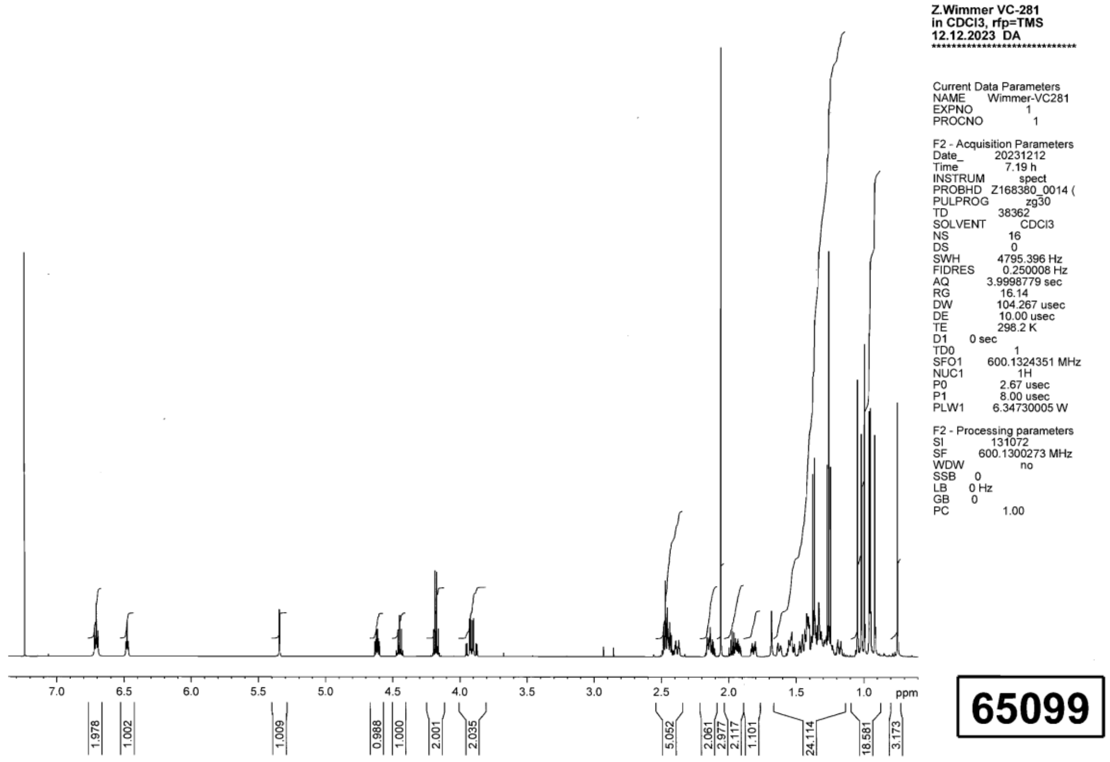
Z.Wimmer VC-281
in CDCl3, rfp=TMS
12.12.2023 DA
Current Data Parameters
NAME Wimmer-VC281
EXPNO 1
PROCNO 1
F2 - Acquisition Parameters
Date_ 20231212
Time 7.19 h
INSTRUM spect
PROBHD Z168380_0014 (
PULPROG zg30
TD 38362
SOLVENT CDCl3
NS 16
DS 0
SWH 4795.396 Hz
FIDRES 0.250008 Hz
AQ 3.9998779 sec
RG 16.14
DW 104.267 usec
DE 10.00 usec
TE 298.2 K
D1 0 sec
TD0 1
SFO1 600.1324351 MHz
NUC1 1H
P0 2.67 usec
P1 8.00 usec
PLW1 6.34730005 W
F2 - Processing parameters
SI 131072
SF 600.1300273 MHz
WDW no
SSB 0
LB 0 Hz
GB 0
PC 1.00
7.0 6.5 6.0 5.5 5.0 4.5 4.0 3.5 3.0 2.5 2.0 1.5 1.0 ppm
1.978 1.002 1.009 0.988 1.000 2.001 2.035 5.052 2.061 2.977 2.117 1.101 24.114 18.581 3.173
65099

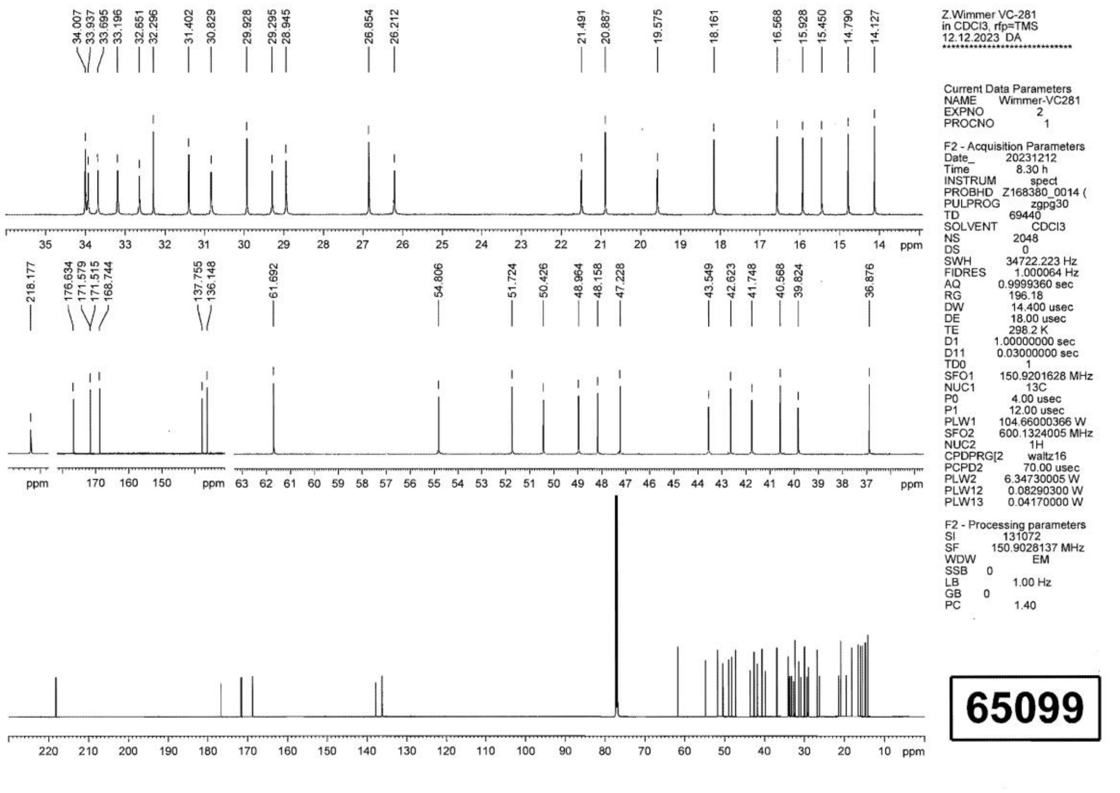
34.007 33.937 33.695 33.196 32.651 32.296 31.402 30.829 29.928 29.295 28.945 26.854 26.212 21.491 20.887 19.575 18.161 16.568 15.928 15.450 14.790 14.127
35 34 33 32 31 30 29 28 27 26 25 24 23 22 21 20 19 18 17 16 15 14 ppm
218.177 176.634 171.579 171.515 168.744 137.755 136.148 61.692 54.806 51.724 50.426 48.964 48.158 47.228 43.549 42.623 41.748 40.568 39.824 36.876
ppm 170 160 150 ppm 63 62 61 60 59 58 57 56 55 54 53 52 51 50 49 48 47 46 45 44 43 42 41 40 39 38 37 ppm
220 210 200 190 180 170 160 150 140 130 120 110 100 90 80 70 60 50 40 30 20 10 ppm
Z.Wimmer VC-281
in CDCl3, rfp=TMS
12.12.2023 DA
Current Data Parameters
NAME Wimmer-VC281
EXPNO 2
PROCNO 1
F2 - Acquisition Parameters
Date_ 20231212
Time 8.30 h
INSTRUM spect
PROBHD Z168380_0014 (
PULPROG zgpg30
TD 69440
SOLVENT CDCl3
NS 2048
DS 0
SWH 34722.223 Hz
FIDRES 1.000064 Hz
AQ 0.9999360 sec
RG 196.18
DW 14.400 usec
DE 18.00 usec
TE 298.2 K
D1 1.00000000 sec
D11 0.03000000 sec
TD0 1
SFO1 150.9201628 MHz
NUC1 13C
P0 4.00 usec
P1 12.00 usec
PLW1 104.66000366 W
SFO2 600.1324005 MHz
NUC2 1H
CPDPRG[2 waltz16
PCPD2 70.00 usec
PLW2 6.34730005 W
PLW12 0.08290300 W
PLW13 0.04170000 W
F2 - Processing parameters
SI 131072
SF 150.9028137 MHz
WDW EM
SSB 0
LB 1.00 Hz
GB 0
PC 1.40
65099

N-(3,28-Dioxoolean-18-en-28-yl)glycyl-L-alanyl-L-methionine (**14**)

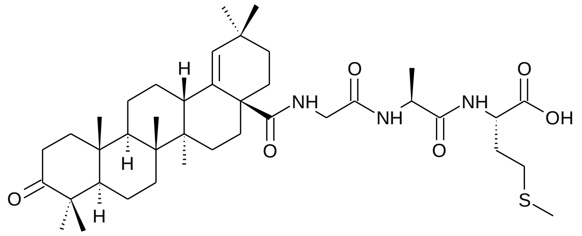

^{1}H NMR (CD_3OD): δ [ppm] 0.84 (3H, s, H27), 0.97 (3H, d, *J*=0.5 Hz, H25), 0.99 (3H, s, H30), 1.03 (3H, s, H24), 1.05 (3H, s, H26), 1.06 (3H, s, H29), 1.07 (3H, s, H23), 1.20 (1H, dt, J_1=3.4 Hz, J_2=3.4 Hz, J_3=13.3 Hz, H15), 1.26-1.32 (1H, H11), 1.28-1.35 (1H, H21), 1.37 (3H, d, *J*=7.2 Hz, H4'), 1.93-1.97 (1H, m, H8'), 1.50-1.59 (1H, m, H15), 1.58 (1H, dt, J_1=2.8 Hz, J_2=13.5 Hz, J_3=13.5 Hz, H2), 1.72 (1H, dq, J_1=3.2 Hz, J_2=3.2 Hz, J_3=3.2 Hz, J_4=12.7 Hz, H12), 1.84 (1H, ddd, J_1=3.0 Hz, J_2=5.3 Hz, J_3=13.5 Hz, H2), 2.07 (3H, s, H10'), 2.11-2.16 (2H, m, H8'), 2.33 (1H, dt, J_1=3.5 Hz, J_2=3.5 Hz, J_3=13.6 Hz, H21), 2.34 (2H, ddd, J_1=1.8 Hz, J_2=3.5 Hz, J_3=12.2 Hz, H13), 2.44-2.58 (2H, m, H7), 2.44-2.58 (2H, m, H9'), 3.88-3.94 (2H, m, H1'), 4.33 (1H, dd, J_1=4.7 Hz, J_2=8.4 Hz, H6'), 4.39 (1H, q, *J*=7.2 Hz, H3'), 5.38 (1H, bs, H19). ^{13}C NMR (CD_3OD): δ [ppm] 15.41 (q, C27), 15.41 (s, C10'), 16.57 (q, C26), 17.21 (q, C25), 18.10 (t, C4'), 20.73 (t, C6), 21.34 (q, C24), 22.76 (t, C11), 27.25 (t, C12), 27.40 (q, C23), 29.36 (q, C30), 30.49 (t, C15), 31.04 (q, C29), 31.04 (q, C8'), 31.11 (t, C21), 31.33 (t, C9'), 31.44 (t, C7), 33.29 (s, C20), 34.25 (t, C16), 34.96 (t, C2), 35.18 (t, C22), 38.08 (s, C10), 40.95 (t, C1), 41.83 (s, C8), 42.88 (d, C13), 43.70 (s, C14), 43.75 (d, C1'), 48.32 (s, C4), 49.45 (s, C17), 50.47 (t, C3'), 51.77 (d, C9), 55.06 (d, C6'), 55.95 (d, C5), 137.07 (d, C19), 138.68 (s, C18), 170.91 (s, C2'), 174.63 (s, C7'), 178.87 (s, C28), 221.00 (s, C3). IR [cm^{-1}]: 3386, 2938, 2869, 1699, 1520, 1419, 1249, 1168. MS: *m/z* = 714.4 $[M+H]^+$ (ESI^+, coin voltage 10 V), *m/z* = 712.4 $[M–H]^-$ (ESI^-, coin voltage 10 V). For $C_{40}H_{63}N_3O_6S$ (714.01) calcd. C 67.29, H 8.89, N 5.89, S 4.49, found C 67.26, H 8.90, N 5.91, S 4.51.

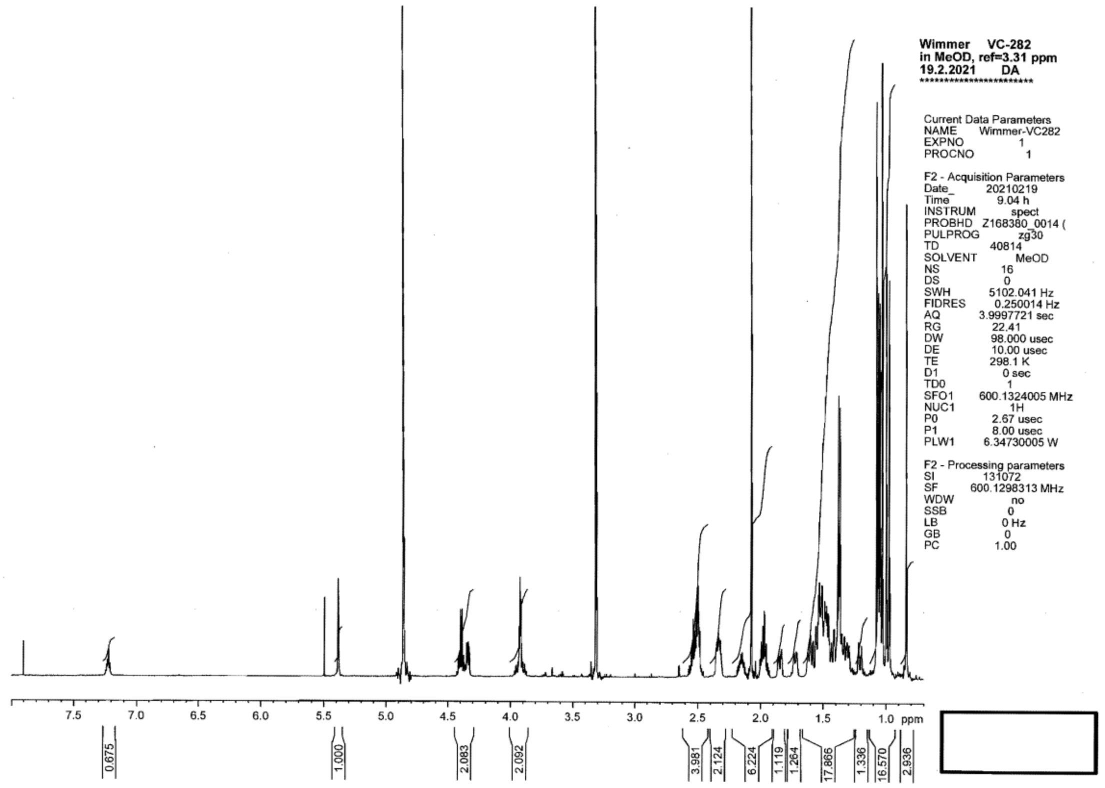

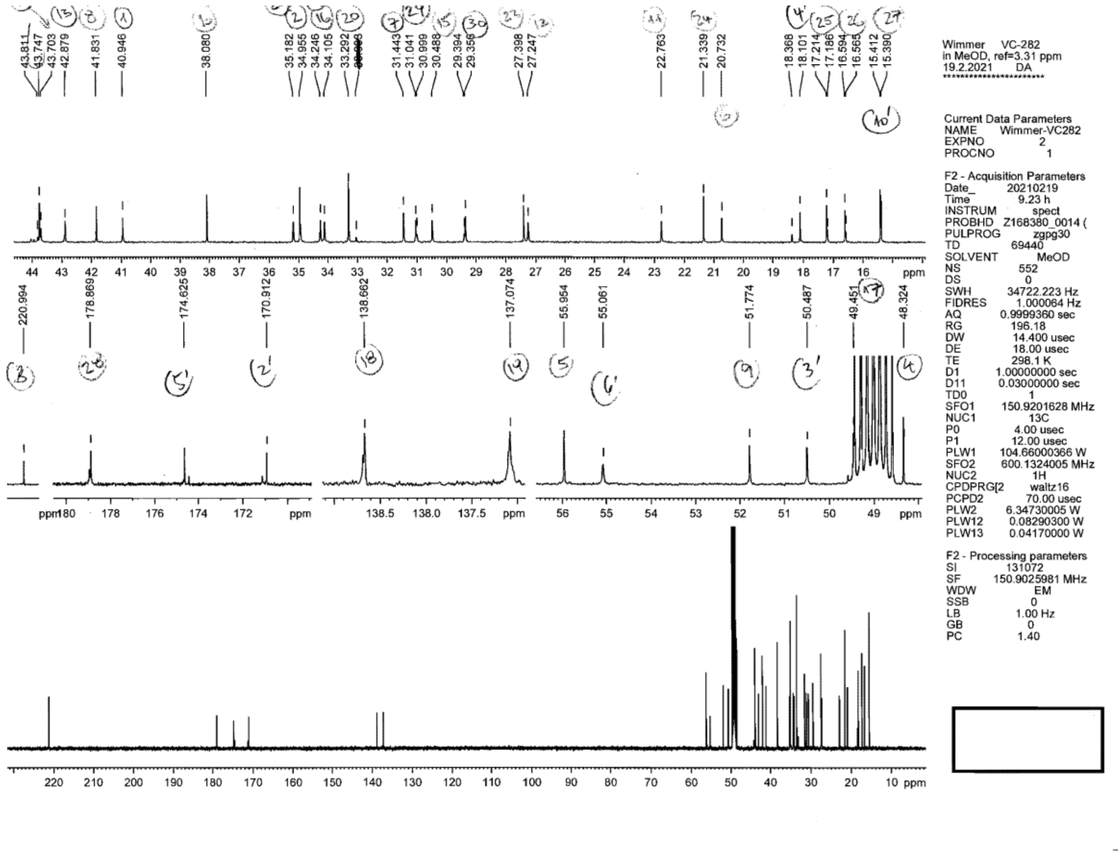

Ethyl *N*-(3,28-dioxoolean-18-en-28-yl)-L-methionyl-L-alanylglycinate (**15**)

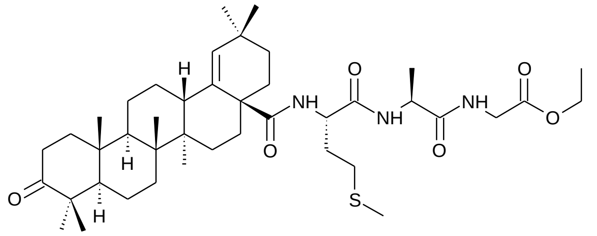

^{1}H NMR (CD_3OD): δ [ppm] 0.76 (3H, s, H27), 0.94 (3H, d, *J*=0.7 Hz, H25), 0.95 (3H, s, H26), 0.98 (3H, s, H30), 1.02 (3H, s, H24), 1.02 (3H, s, H29), 1.07 (3H, s, H23), 1.19 (1H, dt, J_1=3.5 Hz, J_2=3.5 Hz, J_3=13.0 Hz, H15), 1.27 (3H, *J*=7.2 Hz, H12'), 1.41 (3H, d, *J*=7.1 Hz, H8'), 1.84 (2H, ddd, J_1=3.5 Hz, J_2=4.9 Hz, J_3=13.9 Hz, H2), 1.90-1.95 (1H, m, H3'), 2.03-2.09 (1H, m, H3'), 2.10 (3H, s, H5'), 2.16 (1H, ddd, J_1=1.7 Hz, J_2=3.6 Hz, J_3=11.8 Hz, H13), 2.37 (2H, dt, J_1=3.5 Hz, J_2=3.5 Hz, J_3=13.2 Hz, H21), 2.44 (1H, ddd, J_1=4.7 Hz, J_2=8.1 Hz, J_3=15.7 Hz, H7), 2.49 (1H, ddd, J_1=7.7 Hz, J_2=9.1 Hz, J_3=15.7 Hz, H7), 2.51-2.60 (2H, m, H4'), 3.99 (1H, dd, J_1=5.3 Hz, J_2=8.2 Hz, H9'), 4.02 (1H, dd, J_1=5.3 Hz, J_2=18.2 Hz, H9'), 4.20 (2H, q, J_1=7.2 Hz, J_2=7.2 Hz, J_3=7.2 Hz, J_4=7.2 Hz, H11'), 4.47 (1H, m, J_1=7.2 Hz, J_2=7.2 Hz, J_3=7.2 Hz, J_4=7.2 Hz, H6'), 4.55 (1H, dt, J_1=6.9 Hz, J_2=6.9 Hz, J_3=7.8 Hz, H1'), 5.36 (1H, dd, J_1=0.5 Hz, J_2=1.7 Hz, H19), 6.46 (1H, d, *J*=7.8 Hz, 1'-NH), 6.66 (1H, t, *J*=5.3 Hz, 9'-NH), 6.79 (1H, d, *J*=7.4 Hz, 6'-NH). ^{13}C NMR (CD_3OD): δ [ppm] 14.2 (q, C12'), 14.8 (q, C27), 15.3 (q, C5'), 15.8 (q, C26), 16.6 (q, C25), 17.8 (q, C8'), 19.6 (t, C6), 20.9 (q, C24), 21.5 (t, C11), 26.2 (t, C12), 26.8 (q, C23), 28.9 (q, C30), 29.3 (t, C15), 30.2 (t, C4'), 30.9 (q, C29), 31.1 (t, C3'), 32.3 (s, C20), 32.8 (t, C21), 33.4 (t, C16), 33.7 (t, C2), 33.9 (t, C22), 34.0 (t, C7), 36.9 (s, C10), 39.8 (t, C1), 40.6 (s, C8), 41.3 (d, C9'), 41.7 (d, C13), 42.6 (s, C14), 47.2 (s, C4), 48.9 (d, C6'), 50.4 (d, C9), 48.2 (s, C17), 52.4 (d, C1'), 54.8 (d, C5), 61.5 (t, C11'), 136.2 (d, C19), 137.7 (s, C18), 169.5 (s, C10'), 171.0 (s, C2'), 171.8 (s, C7'), 176.4 (s, C28), 218.1 (s, C3). IR [cm^{-1}]: 2936, 2869, 1698, 1520, 1420, 1367, 1250, 1169. MS: *m/z* = 742.5 $[M+H]^+$ (ESI^+, coin voltage 10 V), *m/z* = 740.4 $[M–H]^-$ (ESI^-, coin voltage 10 V). For $C_{42}H_{67}N_3O_6S$ (742.06) calcd. C 67.98, H 9.10, N 5.66, S 4.32, found C 67.95, H 9.11, N 5.67, S 4.30.

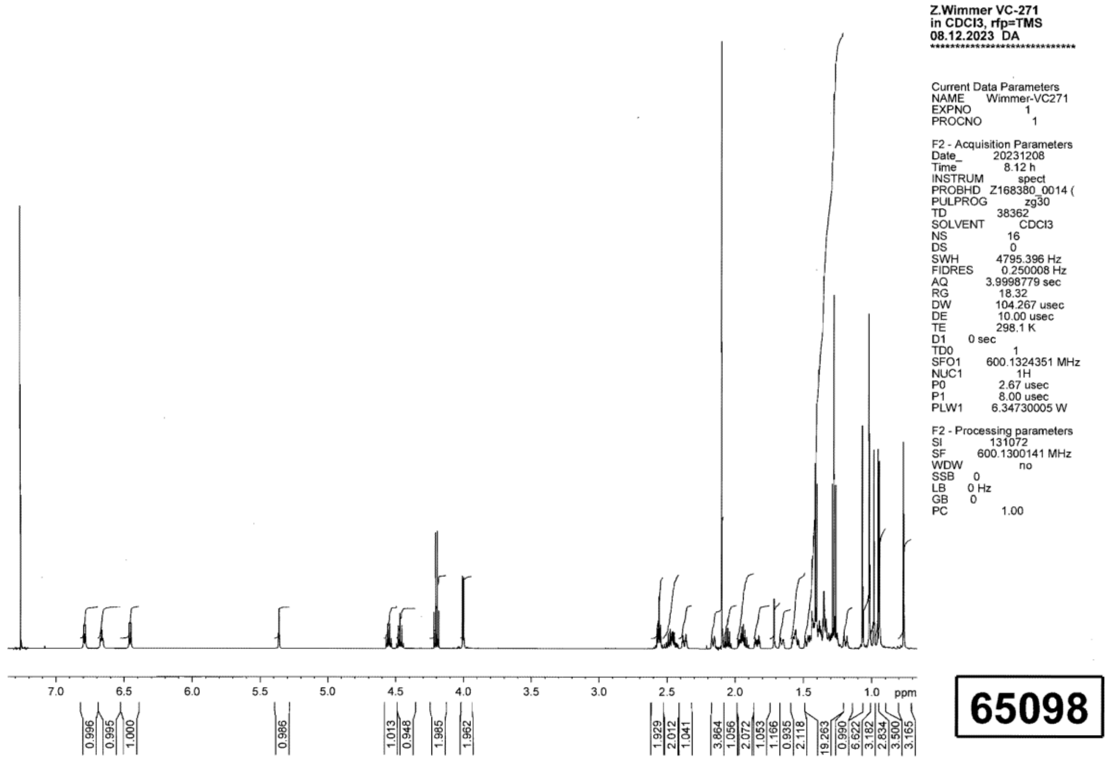
Z.Wimmer VC-271
in CDCl3, rfp=TMS
08.12.2023 DA
Current Data Parameters
NAME Wimmer-VC271
EXPNO 1
PROCNO 1
F2 - Acquisition Parameters
Date_ 20231208
Time 8.12 h
INSTRUM spect
PROBHD Z168380_0014 (
PULPROG zg30
TD 38362
SOLVENT CDCl3
NS 16
DS 0
SWH 4795.396 Hz
FIDRES 0.250008 Hz
AQ 3.9998779 sec
RG 18.32
DW 104.267 usec
DE 10.00 usec
TE 298.1 K
D1 0 sec
TD0 1
SFO1 600.1324351 MHz
NUC1 1H
P0 2.67 usec
P1 8.00 usec
PLW1 6.34730005 W
F2 - Processing parameters
SI 131072
SF 600.1300141 MHz
WDW no
SSB 0
LB 0 Hz
GB 0
PC 1.00
7.0 6.5 6.0 5.5 5.0 4.5 4.0 3.5 3.0 2.5 2.0 1.5 1.0 ppm
0.996 0.995 1.000 0.986 1.013 0.948 1.985 1.962 1.929 2.012 1.041 3.864 1.056 2.072 1.053 1.166 0.935 2.118 19.263 0.990 6.622 3.182 2.834 3.500 3.165
65098

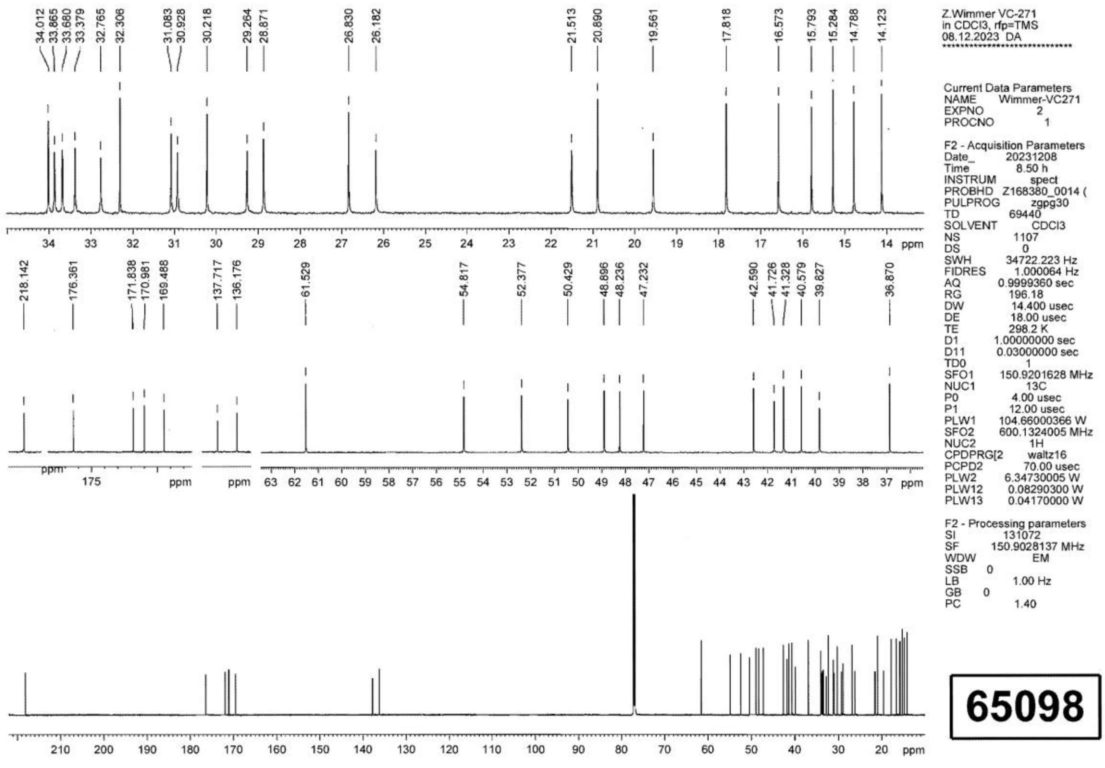
34.012 33.865 33.680 33.379 32.765 32.306 31.083 30.928 30.218 29.264 28.871 26.830 26.182 21.513 20.890 19.561 17.818 16.573 15.793 15.284 14.788 14.123
34 33 32 31 30 29 28 27 26 25 24 23 22 21 20 19 18 17 16 15 14 ppm
218.142 176.361 171.838 170.981 169.488 137.717 136.176 61.529 54.817 52.377 50.429 48.896 48.236 47.232 42.590 41.726 41.328 40.579 39.827 36.870
ppm 175 ppm ppm 63 62 61 60 59 58 57 56 55 54 53 52 51 50 49 48 47 46 45 44 43 42 41 40 39 38 37 ppm
210 200 190 180 170 160 150 140 130 120 110 100 90 80 70 60 50 40 30 20 ppm
Z.Wimmer VC-271
in CDCl3, rfp=TMS
08.12.2023 DA
Current Data Parameters
NAME Wimmer-VC271
EXPNO 2
PROCNO 1
F2 - Acquisition Parameters
Date_ 20231208
Time 8.50 h
INSTRUM spect
PROBHD Z168380_0014 (
PULPROG zgpg30
TD 69440
SOLVENT CDCl3
NS 1107
DS 0
SWH 34722.223 Hz
FIDRES 1.000064 Hz
AQ 0.9999360 sec
RG 196.18
DW 14.400 usec
DE 18.00 usec
TE 298.2 K
D1 1.00000000 sec
D11 0.03000000 sec
TD0 1
SFO1 150.9201628 MHz
NUC1 13C
P0 4.00 usec
P1 12.00 usec
PLW1 104.66000366 W
SFO2 600.1324005 MHz
NUC2 1H
CPDPRG[2 waltz16
PCPD2 70.00 usec
PLW2 6.34730005 W
PLW12 0.08290300 W
PLW13 0.04170000 W
F2 - Processing parameters
SI 131072
SF 150.9028137 MHz
WDW EM
SSB 0
LB 1.00 Hz
GB 0
PC 1.40
65098

N-(3,28-Dioxoolean-18-en-28-yl)-L-methionyl-L-alanylglycine (**16**)

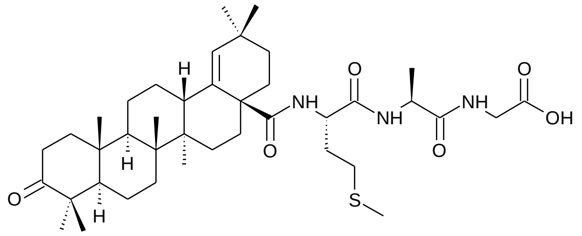

^{1}H NMR (CD_3OD): δ [ppm] 0.83 (3H, s, H27), 0.97 (3H, d, *J*=0.6 Hz, H25), 1.00 (3H, s, H26), 1.01 (3H, s, H30), 1.02 (3H, s, H24), 1.05 (3H, s, H29), 1.07 (3H, s, H23), 1.20 (1H, dt, J_1=3.4 Hz, J_2=3.4 Hz, J_3=13.5 Hz, H15), 1.39 (3H, d, *J*=7.1 Hz, H8'), 1.83 (2H, dt, J_1=4.5 Hz, J_2=4.5 Hz, J_3=13.8 Hz, H2), 1.90-1.96 (1H, m, H3'), 2.03-2.08 (1H, m, H3'), 2.08 (3H, s, H5'), 2.28 (1H, ddd, J_1=1.8 Hz, J_2=3.2 Hz, J_3=12.4 Hz, H13), 2.30 (2H, dt, J_1=3.6 Hz, J_2=3.6 Hz, J_3=13.3 Hz, H21), 2.46-2.55 (2H, m, H7), 2.46-2.55 (2H, m, H4'), 3.77 (1H, d, *J*=17.5 Hz, H9'), 3.86 (1H, d, *J*=17.5 Hz, H9'), 4.42 (1H, q, *J*=7.1 Hz, H6'), 4.58 (1H, ddd, J_1=5.0 Hz, J_2=5.9 Hz, J_3=8.1 Hz, H1'), 5.65 (1H, bd, *J*=1.8 Hz, H19), 6.91 (1H, bd, *J*=8.1 Hz, 1'-NH). ^{13}C NMR (CD_3OD): δ [ppm] 15.32 (q, C27), 15.42 (q, C5'), 16.53 (q, C26), 17.17 (q, C25), 18.23 (q, C8'), 20.70 (t, C6), 21.32 (q, C24), 22.75 (t, C11), 27.24 (t, C12), 27.38 (q, C23), 29.20 (q, C30), 30.40 (t, C15), 31.04 (t, C7), 31.18 (q, C29), 33.26 (s, C20), 33.53 (t, C4'), 34.05 (t, C21), 34.49 (t, C16), 34.91 (t, C22), 34.96 (t, C2), 34.99 (t, C3'), 38.07 (s, C10), 40.93 (t, C1), 41.85 (s, C8), 42.89 (d, C13), 43.46 (d, C9'), 43.70 (s, C14), 48.33 (s, C4), 49.59 (s, C17), 50.33 (d, C6'), 51.73 (d, C9), 53.61 (d, C1'), 55.94 (d, C5), 137.05 (d, C19), 139.03 (s, C18), 172.99 (s, C10'), 174.55 (s, C7'), 175.60 (s, C2'), 178.04 (s, C28), 221.50 (s, C3). IR [cm^{-1}]: 2936, 2868, 1697, 1519, 1420, 1368, 1250, 1168. MS: *m/z* = 714.5 $[M+H]^+$ (ESI^+, coin voltage 20 V), *m/z* = 712.4 $[M–H]^+$ ($ESI^–$, coin voltage 20 V). For $C_{40}H_{63}N_3O_6S$ (714.01) calcd. C 67.29, H 8.89, N 5.89, S 4.49, found C 67.32, H 8.86, N 5.86, S 4.47.

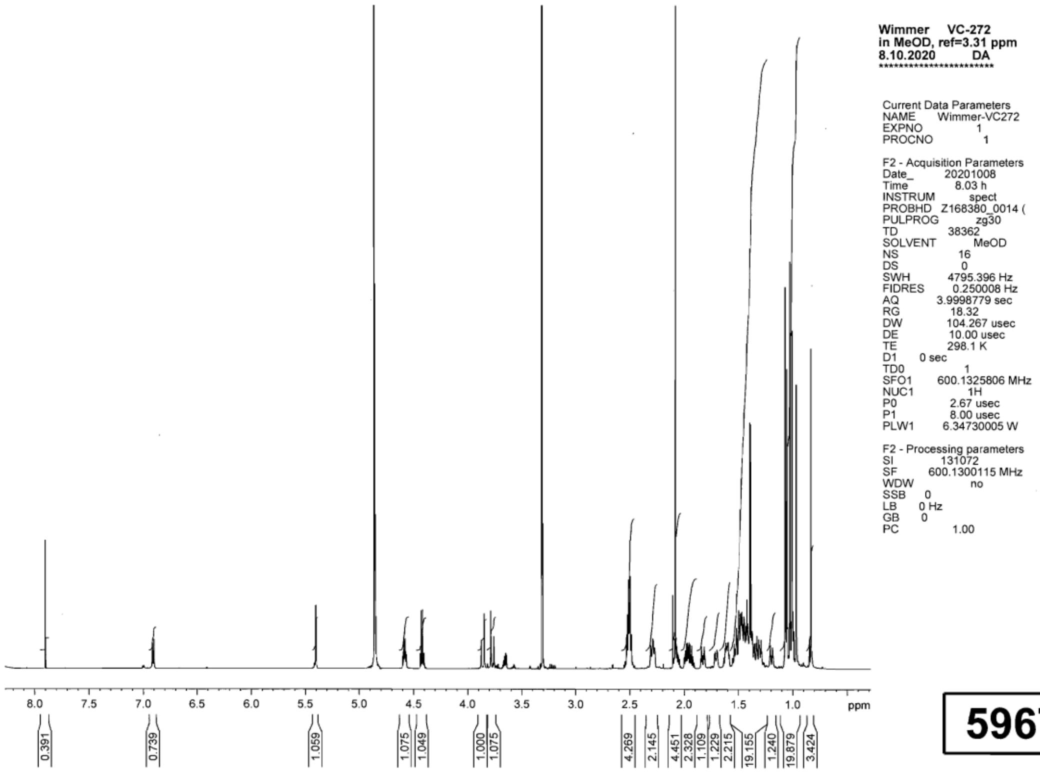
Wimmer VC-272
in MeOD, ref=3.31 ppm
8.10.2020 DA
Current Data Parameters
NAME Wimmer-VC272
EXPNO 1
PROCNO 1
F2 - Acquisition Parameters
Date_ 20201008
Time 8.03 h
INSTRUM spect
PROBHD Z168380_0014 (
PULPROG zg30
TD 38362
SOLVENT MeOD
NS 16
DS 0
SWH 4795.396 Hz
FIDRES 0.250008 Hz
AQ 3.9998779 sec
RG 18.32
DW 104.267 usec
DE 10.00 usec
TE 298.1 K
D1 0 sec
TD0 1
SFO1 600.1325806 MHz
NUC1 1H
P0 2.67 usec
P1 8.00 usec
PLW1 6.34730005 W
F2 - Processing parameters
SI 131072
SF 600.1300115 MHz
WDW no
SSB 0
LB 0 Hz
GB 0
PC 1.00
8.0 7.5 7.0 6.5 6.0 5.5 5.0 4.5 4.0 3.5 3.0 2.5 2.0 1.5 1.0 ppm
0.391
0.739
1.059
1.075
1.049
1.000
1.075
4.269
2.145
4.451
2.328
1.109
1.229
2.215
19.155
1.240
19.879
3.424

59679

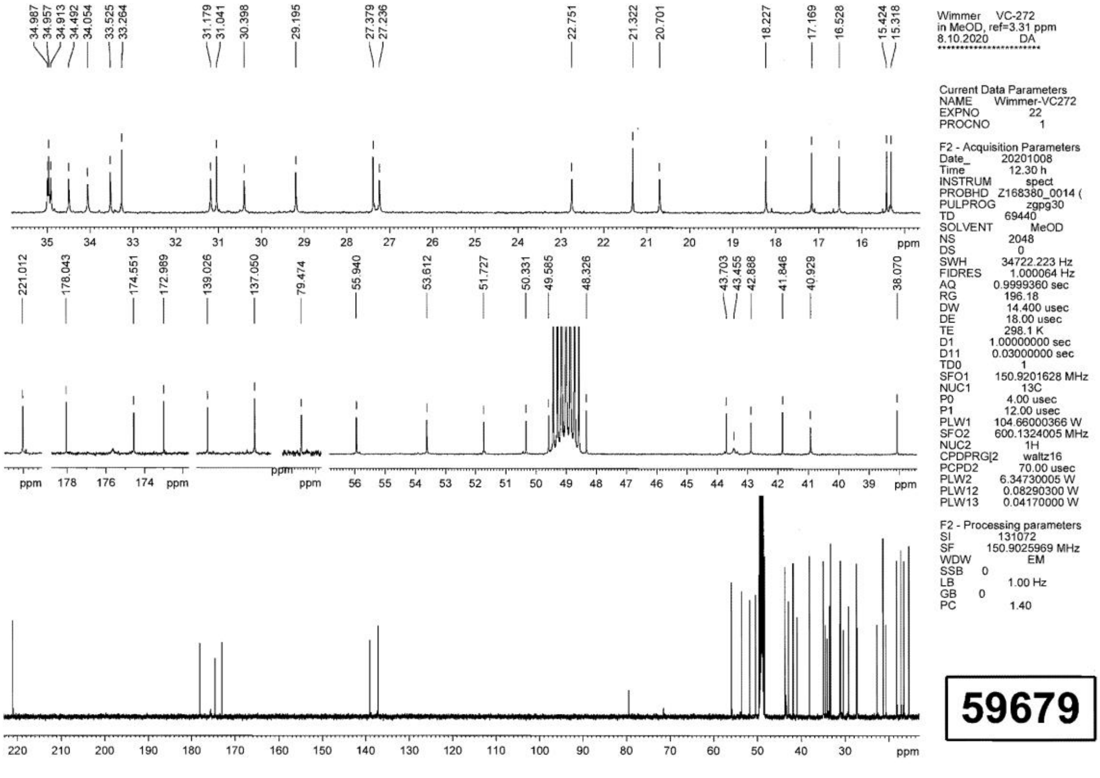
34.987
34.957
34.913
34.492
34.054
33.525
33.264
31.179
31.041
30.398
29.195
27.379
27.236
22.751
21.322
20.701
18.227
17.169
16.528
15.424
15.318
35 34 33 32 31 30 29 28 27 26 25 24 23 22 21 20 19 18 17 16 ppm
221.012
178.043
174.551
172.989
139.026
137.050
79.474
55.940
53.612
51.727
50.331
49.585
48.326
43.703
43.455
42.888
41.846
40.929
38.070
ppm 178 176 174 ppm
ppm
ppm
56 55 54 53 52 51 50 49 48 47 46 45 44 43 42 41 40 39 ppm
220 210 200 190 180 170 160 150 140 130 120 110 100 90 80 70 60 50 40 30 ppm
Wimmer VC-272
in MeOD, ref=3.31 ppm
8.10.2020 DA
Current Data Parameters
NAME Wimmer-VC272
EXPNO 22
PROCNO 1
F2 - Acquisition Parameters
Date_ 20201008
Time 12.30 h
INSTRUM spect
PROBHD Z168380_0014 (
PULPROG zgpg30
TD 69440
SOLVENT MeOD
NS 2048
DS 0
SWH 34722.223 Hz
FIDRES 1.000064 Hz
AQ 0.9999360 sec
RG 196.18
DW 14.400 usec
DE 18.00 usec
TE 298.1 K
D1 1.00000000 sec
D11 0.03000000 sec
TD0 1
SFO1 150.9201628 MHz
NUC1 13C
P0 4.00 usec
P1 12.00 usec
PLW1 104.66000366 W
SFO2 600.1324005 MHz
NUC2 1H
CPDPRG[2 waltz16
PCPD2 70.00 usec
PLW2 6.34730005 W
PLW12 0.08290300 W
PLW13 0.04170000 W
F2 - Processing parameters
SI 131072
SF 150.9025969 MHz
WDW EM
SSB 0
LB 1.00 Hz
GB 0
PC 1.40

59679

(3β)-3-(Acetyloxy)olean-18-en-28-oic acid (**17**)

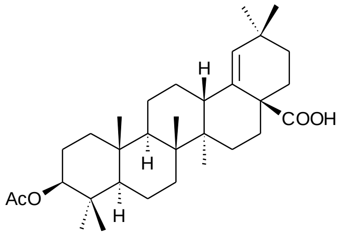

^{1}H NMR ($CDCl_3$): δ [ppm] 0.78 (3H, d, *J*=0.5 Hz, H27), 0.81 (1H, dd, J_1=2.1 Hz, J_2=11.4 Hz, H5), 0.84 (3H, s, H25), 0.85 (3H, s, H23), 0.90 (3H, d, *J*=0.8 Hz, H24), 0.98 (3H, s, H30), 1.00 (3H, d, *J*=0.5 Hz, H26), 1.00 (3H, s, H29), 1.03 (1H, bdt, J_1=4.8 Hz, J_2=13.0 Hz, J_3=13.0 Hz, H1), 1.21 (2H, ddd, J_1=3.0 Hz, J_2=3.9 Hz, J_3=13.4 Hz, H15), 1.75 (1H, dt, J_1=3.6 Hz, J_2=3.6 Hz, J_3=13.2 Hz, H1), 2.00 (2H, ddd, J_1=3.4 Hz, J_2=6.2 Hz, J_3=13.9 Hz, H7), 2.04 (3H, s, H2'), 2.17 (2H, ddd, J_1=3.0 Hz, J_2=4.0 Hz, J_3=13.5 Hz, H16), 2.23 (1H, ddd, J_1=1.9 Hz, J_2=3.4 Hz, J_3=11.4 Hz, H13), 4.49 (1H, dd, J_1=5.3 Hz, J_2=11.3 Hz, H3), 5.18 (1H, dd, J_1=0.1 Hz, J_2=1.9 Hz, H19). ^{13}C NMR ($CDCl_3$): δ [ppm] 14.85 (q, C27), 16.05 (q, C26), 16.51 (q, C25), 16.69 (q, C24), 18.19 (t, C6), 21.00 (t, C11), 21.23 (q, C2'), 23.75 (t, C2), 26.02 (t, C12), 27.96 (q, C23), 29.12 (q, C30), 29.41 (t, C15), 30.32 (q, C29), 32.08 (s, C20), 33.42 (t, C16), 33.55 (t, C7), 33.56 (t, C21), 34.58 (t, C22), 37.24 (s, C10), 37.89 (s, C8), 38.71 (t, C1), 40.79 (s, C14), 41.42 (d, C13), 42.67 (s, C4), 48.00 (s, C17), 51.17 (d, C9), 55.70 (d, C5), 81.00 (d, C3), 133.36 (d, C19), 136.77 (s, C18), 170.94 (s, C1'), 180.54 (s, C28). IR [cm^{-1}]: 3219, 2926, 2855, 1728, 1369, 1252, 1180. MS: *m/z* = 439 $[M–AcOH+H]^+$, 516 $[M+NH_4]^+$ (ESI^+), 498 $[M–H]^+$ ($ESI^–$). For $C_{32}H_{50}O_4$ (498.74) calcd. C 77.06, H 10.10, found C 77.09, H 10.08.

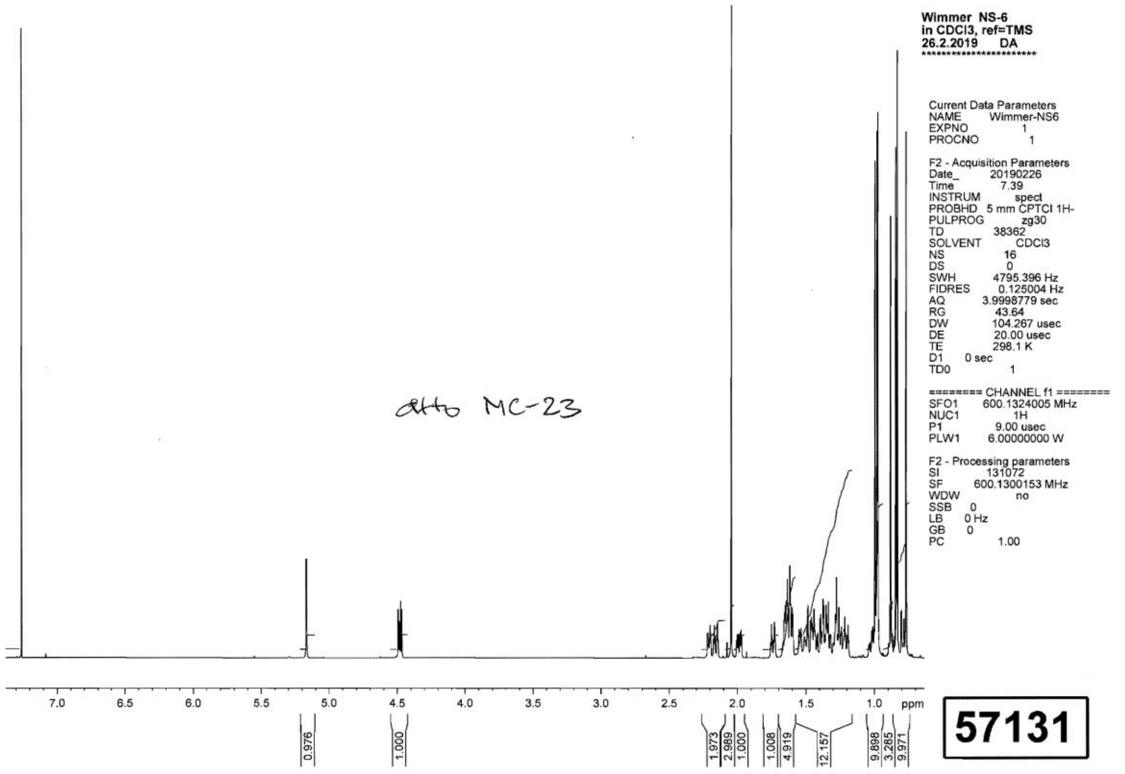
Wimmer NS-6
in CDCl3, ref=TMS
26.2.2019 DA
Current Data Parameters
NAME Wimmer-NS6
EXPNO 1
PROCNO 1
F2 - Acquisition Parameters
Date_ 20190226
Time 7.39
INSTRUM spect
PROBHD 5 mm CPTCI 1H-
PULPROG zg30
TD 38362
SOLVENT CDCl3
NS 16
DS 0
SWH 4795.396 Hz
FIDRES 0.125004 Hz
AQ 3.9998779 sec
RG 43.64
DW 104.267 usec
DE 20.00 usec
TE 298.1 K
D1 0 sec
TD0 1
======== CHANNEL f1 ========
SFO1 600.1324005 MHz
NUC1 1H
P1 9.00 usec
PLW1 6.00000000 W
F2 - Processing parameters
SI 131072
SF 600.1300153 MHz
WDW no
SSB 0
LB 0 Hz
GB 0
PC 1.00
57131
0.976
1.000
1.973
2.989
1.000
1.008
4.919
12.157
9.898
3.285
9.971
ppm

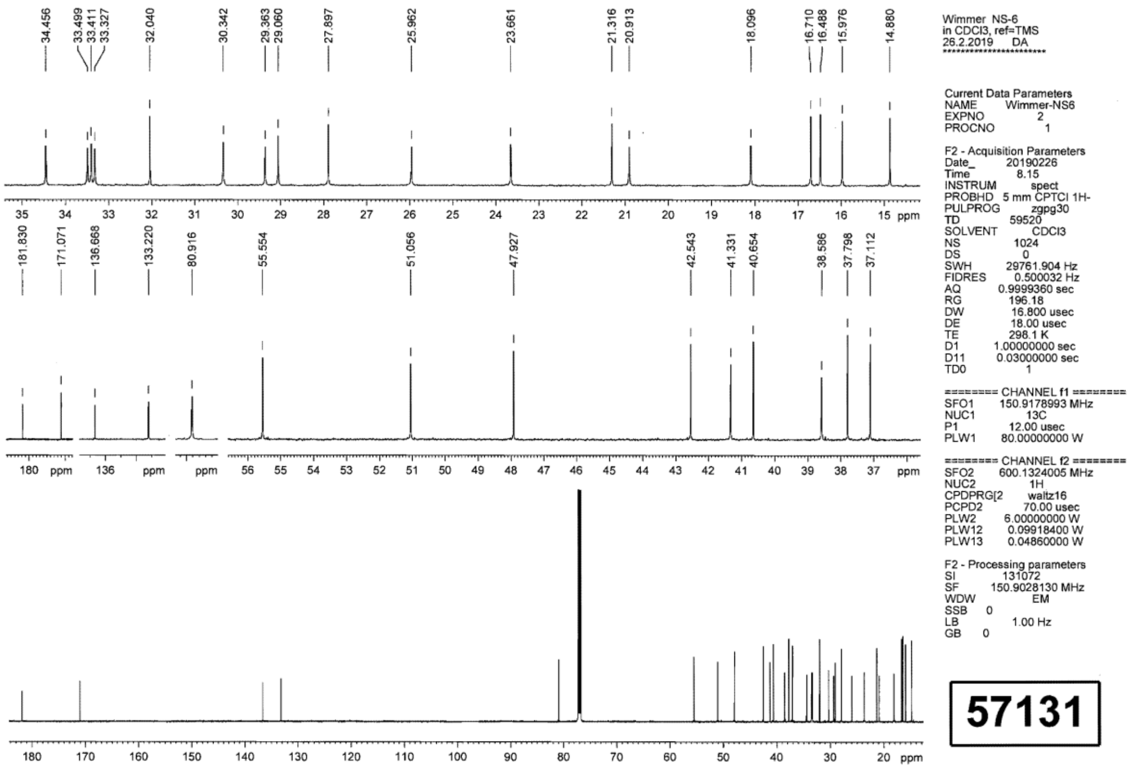
34.456
33.499
33.411
33.327
32.040
30.342
29.363
29.060
27.897
25.962
23.661
21.316
20.913
18.096
16.710
16.488
15.976
14.880
181.830
171.071
136.668
133.220
80.916
55.554
51.056
47.927
42.543
41.331
40.654
38.586
37.798
37.112
Wimmer NS-6
in CDCl3, ref=TMS
26.2.2019 DA
Current Data Parameters
NAME Wimmer-NS6
EXPNO 2
PROCNO 1
F2 - Acquisition Parameters
Date_ 20190226
Time 8.15
INSTRUM spect
PROBHD 5 mm CPTCI 1H-
PULPROG zgpg30
TD 59520
SOLVENT CDCl3
NS 1024
DS 0
SWH 29761.904 Hz
FIDRES 0.500032 Hz
AQ 0.9999360 sec
RG 196.18
DW 16.800 usec
DE 18.00 usec
TE 298.1 K
D1 1.00000000 sec
D11 0.03000000 sec
TD0 1
======== CHANNEL f1 ========
SFO1 150.9178993 MHz
NUC1 13C
P1 12.00 usec
PLW1 80.00000000 W
======== CHANNEL f2 ========
SFO2 600.1324005 MHz
NUC2 1H
CPDPRG[2 waltz16
PCPD2 70.00 usec
PLW2 6.00000000 W
PLW12 0.09918400 W
PLW13 0.04860000 W
F2 - Processing parameters
SI 131072
SF 150.9028130 MHz
WDW EM
SSB 0
LB 1.00 Hz
GB 0
57131
ppm

Ethyl (3β)-*N*-[3-(acetyloxy)-28-oxoolean-18-en-28-yl]glycyl-L-alanyl-L-methioninate (**18**)

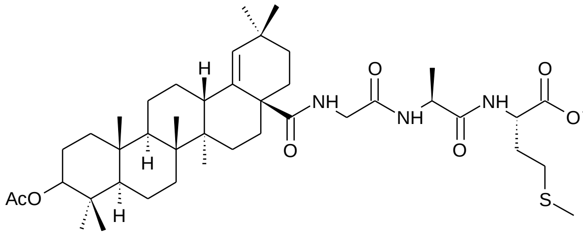

^{1}H NMR (CD_3OD): δ [ppm] 0.75 (3H, s, H27), 0.78 (1H, dd, J_1=2.1 Hz, J_2=11.2 Hz, H5), 0.82 (3H, s, H25), 0.83 (3H, s, H23), 0.88 (3H, s, H24), 0.94 (3H, s, H26), 0.98 (3H, s, H30), 1.03 (3H, s, H29), 1.18 (1H, dt, J_1=3.3 Hz, J_2=3.3 Hz, J_3=13.4 Hz, H15), 1.28 (3H, t, *J*=7.2 Hz), 1.39 (3H, d, J=7.1 Hz, H5'), 1.74 (2H, dt, J_1=3.6 Hz, J_2=3.6 Hz, J_3=13.4 Hz, H1), 1.83 (2H, ddd, J_1=3.4 Hz, J_2=4.9 Hz, J_3=13.8 Hz, H16), 1.96-2.03 (1H, d, H8'), 2.04 (3H, s, **CH_3**CO), 2.08 (3H, s, H10'), 2.12-2.19 (1H, d, H8'), 2.39 (2H, dt, J_1=3.5 Hz, J_2=3.5 Hz, J_3=13.4 Hz, H7), 2.45-2.53 (2H, d, H9'), 3.90 (1H, dd, J_1=5.5 Hz, J_2=16.4 Hz, H1'), 3.93 (1H, dd, J_1=5.1 Hz, J_2=16.4 Hz, H1'), 4.20 (2H, dq, J_1=0.8 Hz, J_2=7.2 Hz, J_3=7.2 Hz, J_4=7.2 Hz, H11'), 4.47 (1H, dd, J_1=5.3 Hz, J_2=11.2 Hz, H3), 4.47 (1H, m, J_1=7.2 Hz, J_2=7.2 Hz, J_3=7.2 Hz, J_4=7.2 Hz, H3'), 4.64 (1H, dt, J_1=5.1 Hz, J_2=7.6 Hz, J_3=7.6 Hz, H6'), 5.37 (1H, dd, J_1=0.3 Hz, J_2=1.7 Hz, H19), 6.50 (1H, t, *J*=5.3 Hz, 1'-NH), 6.74 (1H, d, *J*=7.4 Hz, 3'-NH), 6.76 (1H, d, *J*=7.9 Hz, 6'-NH). ^{13}C NMR (CD_3OD): δ [ppm] 14.1 (q, C12'), 14.9 (q, C27), 15.4 (q, C10'), 16.1 (q, C26), 16.5 (q, C25), 16.8 (q, C24), 18.1 (t, C6), 18.1 (q, C5'), 20.9 (t, C11), 21.3 (q, **CH_3**CO), 23.6 (t, C2), 26.2 (t, C12), 27.9 (q, C23), 28.9 (q, C30), 29.3 (t, C15), 29.9 (d, C9'), 30.8 (q, C29), 31.4 (t, C8'), 32.3 (s, C20), 32.7 (t, C7), 33.2 (t, C21), 34.0 (t, C16), 34.4 (t, C22), 37.1 (s, C10), 37.8 (s, C4), 38.6 (t, C1), 40.7 (s, C8), 41.7 (d, C13), 42.6 (s, C14), 43.6 (d, C1'), 48.2 (s, C17), 49.0 (d, C3'), 51.1 (d, C9), 51.7 (d, C6'), 51.7 (t,C11'), 55.5 (d, C5), 80.9 (d, C3), 136.1 (d, C19), 137.8 (s, C18), 168.8 (s, C2'), 171.0 (s, CH_3**CO**), 171.5 (s, C7'), 171.6 (s, C4'), 176.8 (s, C28). IR [cm^{-1}]: 3377, 2945, 2876, 1678, 1525, 1476, 1419, 1366, 1247, 1168, 1027, 755. MS: *m/z* = 786.5 $[M+H]^+$ (ESI^+, coin voltage 20 V), *m/z* = 784.4 $[M–H]^+$ (ESI^-, coin voltage 20 V).

For $C_{44}H_{71}N_3O_7S$ (786.12) calcd. C 67.23, H 9.10, N 5.35, S 4.08, found C 67.26, H 9.08, N 5.37, S 4.11.

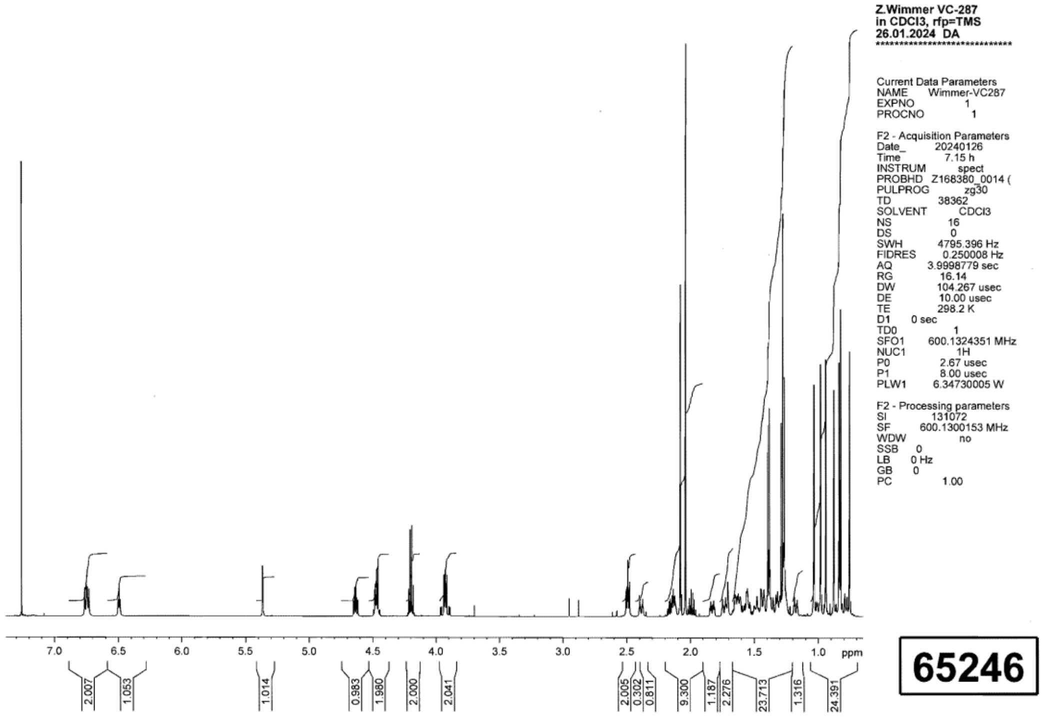

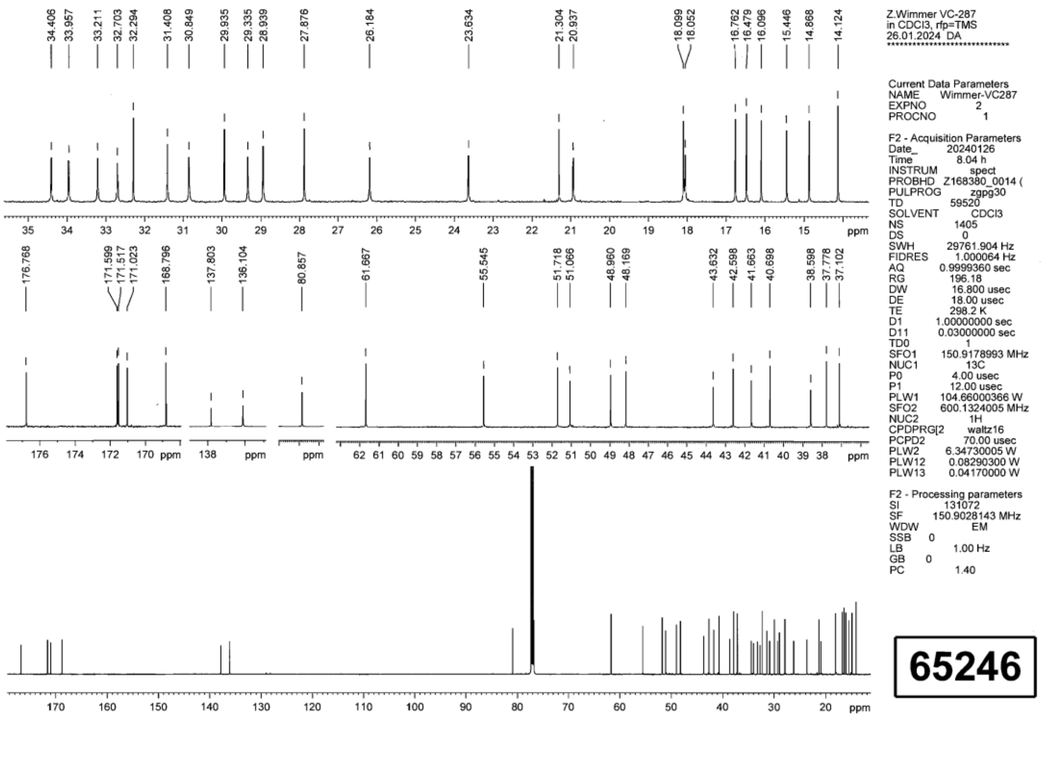

(3β)-*N*-(3-Hydroxy-28-oxoolean-18-en-28-yl)glycyl-L-alanyl-L-methionine (**19**)

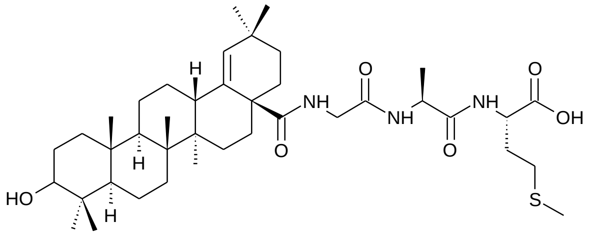

^{1}H NMR (CD_3OD): δ [ppm] 0.72 (1H, dd, J_1=2.1 Hz, J_2=11.2 Hz, H5), 0.76 (3H, s, H25), 0.82 (3H, s, H27), 0.90 (3H, s, H24), 0.95 (3H, s, H23), 0.99 (3H, s, H26), 1.05 (3H, s, H29), 1.37 (3H, *J*=7.2 Hz, H4'), 1.76 (2H, dt, J_1=3.5 Hz, J_1=3.5 Hz, J_2=13.1 Hz, H1), 1.84 (2H, ddd, J_1=2.5 Hz, J_2=4.7 Hz, J_3=13.0 Hz, H2), 1.91 (3H, s, H30), 1.93-2.00 (1H, m, H8'), 2.07 (3H, s, H10'), 2.12-2.17 (1H, m, H8'), 2.28-2.33 (3H, m, H13 + H21), 2.47-2.58 (2H, m, H9'), 3.15 (1H, dd, J_1=4.8 Hz, J_2=11.6 Hz, H3), 3.90-3.93 (3H, m, H1'), 4.36 (1H, dd, J_1=4.7 Hz, J_2=8.6 Hz, H6'), 4.38 (1H, *J*=7.2 Hz, H3'), 5.37 (1H, bs, H19). ^{13}C NMR (CD_3OD): δ [ppm] 15.4 (q, C10'), 15.5 (q, C27), 16.1 (q, C25), 16.8 (q, C26), 17.4 (q, C24), 18.1 (q, C4'), 19.4 (t, C6), 22.2 (t, C11), 27.3 (t, C2), 28.1 (t, C12), 28.6 (q, C23), 29.4 (q, C30), 30.5 (t, C15), 31.0 (q, C29), 31.4 (t, C9'), 33.1 (t, C7), 33.3 (s, C20), 34.2 (t, C16), 34.2 (t, C21), 35.2 (t, C8'), 35.8 (t, C22), 38.4 (s, C10), 40.0 (s, C4), 40.3 (t, C1), 42.0 (s, C8), 42.8 (d, C13), 43.7 (s, C14), 43.7 (t, C1'), 49.6 (s, C17), 50.5 (d, C3'), 52.7 (d, C9), 54.7 (d, C6'), 57.0 (d, C5), 79.6 (d, C3), 137.0 (d, C19), 138.8 (s, C18), 170.9 (s, C2'), 174.7 (s, C7'), 178.9 (s, C28). IR [cm^{-1}]: 3388, 2942, 2870, 1678, 1528, 1477, 1419, 1366, 1249, 1168. MS: *m/z* = 716.4 $[M+H]^+$ (ESI^+, coin voltage 20 V), *m/z* = 714.4 $[M–H]^+$ ($ESI^–$, coin voltage 20 V). For $C_{40}H_{65}N_3O_6S$ (716.03) calcd. C 67.10, H 9.15, N 5.87, S 4.48, found C 67.13, H 9.13, N 5.85, S 4.50.

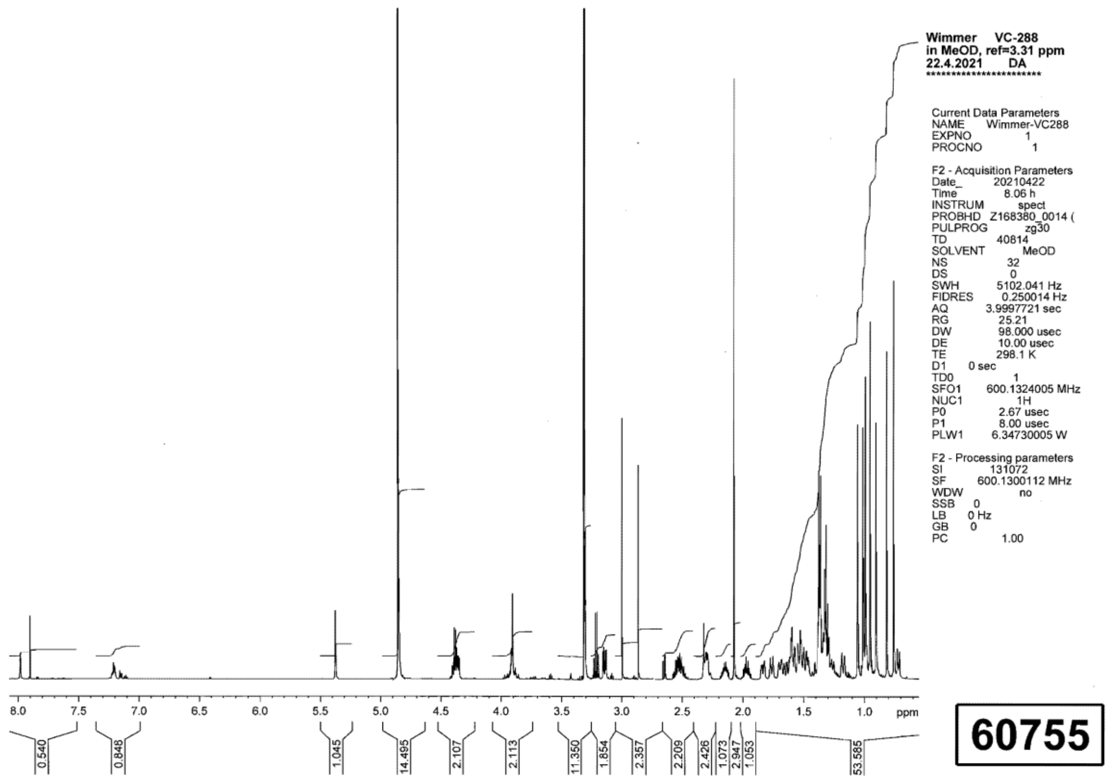
Wimmer VC-288
in MeOD, ref=3.31 ppm
22.4.2021 DA
Current Data Parameters
NAME Wimmer-VC288
EXPNO 1
PROCNO 1
F2 - Acquisition Parameters
Date_ 20210422
Time 8.06 h
INSTRUM spect
PROBHD Z168380_0014 (
PULPROG zg30
TD 40814
SOLVENT MeOD
NS 32
DS 0
SWH 5102.041 Hz
FIDRES 0.250014 Hz
AQ 3.9997721 sec
RG 25.21
DW 98.000 usec
DE 10.00 usec
TE 298.1 K
D1 0 sec
TD0 1
SFO1 600.1324005 MHz
NUC1 1H
P0 2.67 usec
P1 8.00 usec
PLW1 6.34730005 W
F2 - Processing parameters
SI 131072
SF 600.1300112 MHz
WDW no
SSB 0
LB 0 Hz
GB 0
PC 1.00
ppm
0.540
0.848
1.045
14.495
2.107
2.113
11.350
1.854
2.357
2.209
2.426
1.073
2.947
1.053
53.585
60755

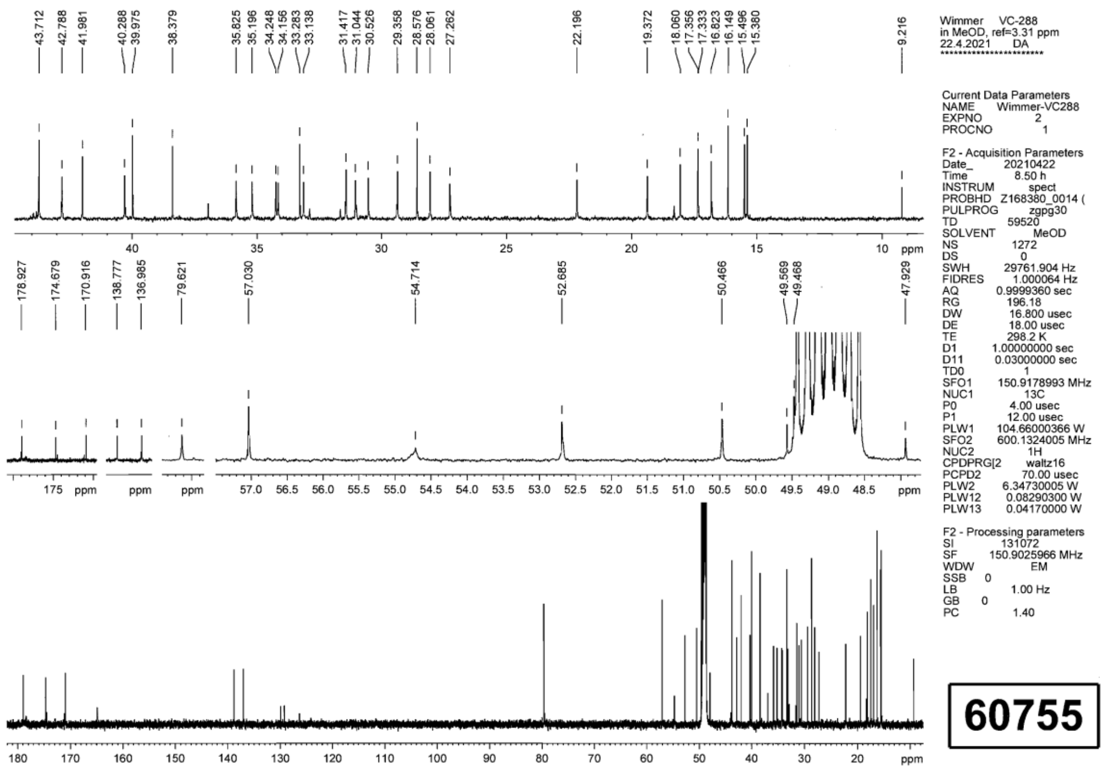
43.712
42.788
41.981
40.288
39.975
38.379
35.825
35.196
34.248
34.156
33.283
33.138
31.417
31.044
30.526
29.358
28.576
28.061
27.262
22.196
19.372
18.060
17.356
17.333
16.823
16.149
15.496
15.380
9.216
178.927
174.679
170.916
138.777
136.985
79.621
57.030
54.714
52.685
50.466
49.569
49.468
47.929
Wimmer VC-288
in MeOD, ref=3.31 ppm
22.4.2021 DA
Current Data Parameters
NAME Wimmer-VC288
EXPNO 2
PROCNO 1
F2 - Acquisition Parameters
Date_ 20210422
Time 8.50 h
INSTRUM spect
PROBHD Z168380_0014 (
PULPROG zgpg30
TD 59520
SOLVENT MeOD
NS 1272
DS 0
SWH 29761.904 Hz
FIDRES 1.000064 Hz
AQ 0.9999360 sec
RG 196.18
DW 16.800 usec
DE 18.00 usec
TE 298.2 K
D1 1.00000000 sec
D11 0.03000000 sec
TD0 1
SFO1 150.9178993 MHz
NUC1 13C
P0 4.00 usec
P1 12.00 usec
PLW1 104.66000366 W
SFO2 600.1324005 MHz
NUC2 1H
CPDPRG[2 waltz16
PCPD2 70.00 usec
PLW2 6.34730005 W
PLW12 0.08290300 W
PLW13 0.04170000 W
F2 - Processing parameters
SI 131072
SF 150.9025966 MHz
WDW EM
SSB 0
LB 1.00 Hz
GB 0
PC 1.40
ppm
60755

(3β)-*N*-[3-(Acetyloxy)-28-oxoolean-18-en-28-yl]glycyl-L-alanyl-L-methionine (**20**)

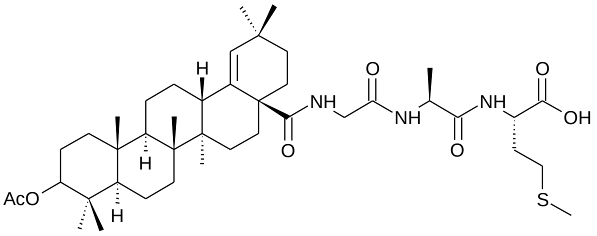

^{1}H NMR (CD_3OD): δ [ppm] 0.83 (3H, s, H27), 0.85 (1H, dd, J_1=1.8 Hz, J_2=11.3 Hz, H5), 0.87 (3H, s, H25), 0.86 (3H, s, H23), 0.94 (3H, s, H24), 0.99 (3H, s, H30), 1.02 (3H, s, H26), 1.05 (3H, s, H29), 1.36 (3H, *J*=7.2 Hz, H4'), 1.79 (2H, dt, J_1=3.7 Hz, J_1=3.7 Hz, J_2=13.1 Hz, H1), 1.83 (2H, ddd, J_1=2.6 Hz, J_2=4.8 Hz, J_3=13.0 Hz, H2), 1.96-2.02 (1H, m, H8'), 2.03 (3H, s, H12'), 2.08 (3H, s, H10'), 2.13-2.18 (1H, m, H8'), 2.27-2.33 (3H, m, H13 + H21), 2.46-2.60 (2H, m, H9'), 3.86 (1H, dd, J_1=5.2 Hz, J_2=16.4 Hz, H1'), 3.92 (1H, dd, J_1=6.7 Hz, J_2=16.4 Hz, H1'), 4.39 (1H, *J*=7.2 Hz, H3'), 4.45 (1H, dd, J_1=5.0 Hz, J_2=11.5 Hz, H3), 4.47-4.49 (1H, m, H6'), 5.48 (1H, bs, H19). ^{13}C NMR (CD_3OD): δ [ppm] 15.3 (q, C10'), 15.5 (q, C27), 16.8 (q, C25), 17.0 (q, C26), 17.4 (q, C24), 18.0 (q, C4'), 19.2 (t, C6), 21.1 (q, C12'), 22.2 (t, C11), 24.7 (t, C2), 27.2 (t, C12), 28.4 (q, C23), 29.3 (q, C30), 30.5 (t, C15), 31.0 (q, C29), 31.3 (t, C9'), 32.5 (t, C8'), 33.3 (t, C7), 34.2 (s, C20), 34.3 (t, C21), 35.2 (t, C16), 35.7 (t, C22), 38.3 (s, C10), 38.9 (s, C4), 39.8 (t, C1), 42.0 (s, C8), 42.8 (d, C13), 43.7 (s, C14), 43.8 (t, C1'), 49.6 (s, C17), 50.3 (d, C3'), 52.6 (d, C9), 53.2 (d, C6'), 57.0 (d, C5), 82.5 (d, C3), 137.0 (d, C19), 138.8 (s, C18), 170.9 (s, C2'), 172.9 (s, C11'), 175.0 (s, C7'), 178.9 (s, C28). IR [cm^{-1}]: 3365, 2937, 1689, 1417, 1364, 1244, 1025, 979, 771. MS: *m/z* = 758.7 $[M+H]^+$ (ESI^+, coin voltage 20 V), *m/z* = 756.6 $[M–H]^+$ ($ESI^–$, coin voltage 20 V). For $C_{42}H_{67}N_3O_7S$ (758.06) calcd. C 66.54, H 8.91, N 5.54, S 4.23, found C 66.51, H 8.93, N 5.51, S 4.25.

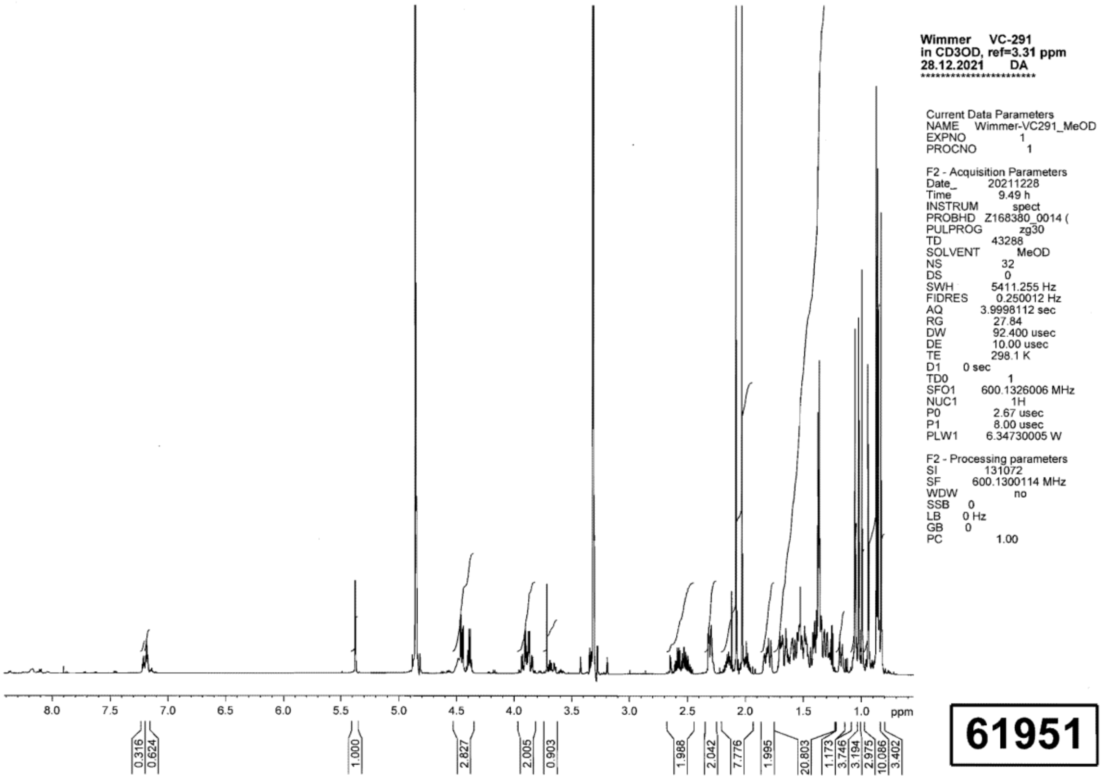
Wimmer VC-291
in CD3OD, ref=3.31 ppm
28.12.2021 DA
Current Data Parameters
NAME Wimmer-VC291_MeOD
EXPNO 1
PROCNO 1
F2 - Acquisition Parameters
Date_ 20211228
Time 9.49 h
INSTRUM spect
PROBHD Z168380_0014 (
PULPROG zg30
TD 43288
SOLVENT MeOD
NS 32
DS 0
SWH 5411.255 Hz
FIDRES 0.250012 Hz
AQ 3.9998112 sec
RG 27.84
DW 92.400 usec
DE 10.00 usec
TE 298.1 K
D1 0 sec
TD0 1
SFO1 600.1326006 MHz
NUC1 1H
P0 2.67 usec
P1 8.00 usec
PLW1 6.34730005 W
F2 - Processing parameters
SI 131072
SF 600.1300114 MHz
WDW no
SSB 0
LB 0 Hz
GB 0
PC 1.00
8.0 7.5 7.0 6.5 6.0 5.5 5.0 4.5 4.0 3.5 3.0 2.5 2.0 1.5 1.0 ppm
0.316 0.624 1.000 2.827 2.005 0.903 1.988 2.042 7.776 1.995 20.803 1.173 3.746 3.194 2.975 10.086 3.402
61951

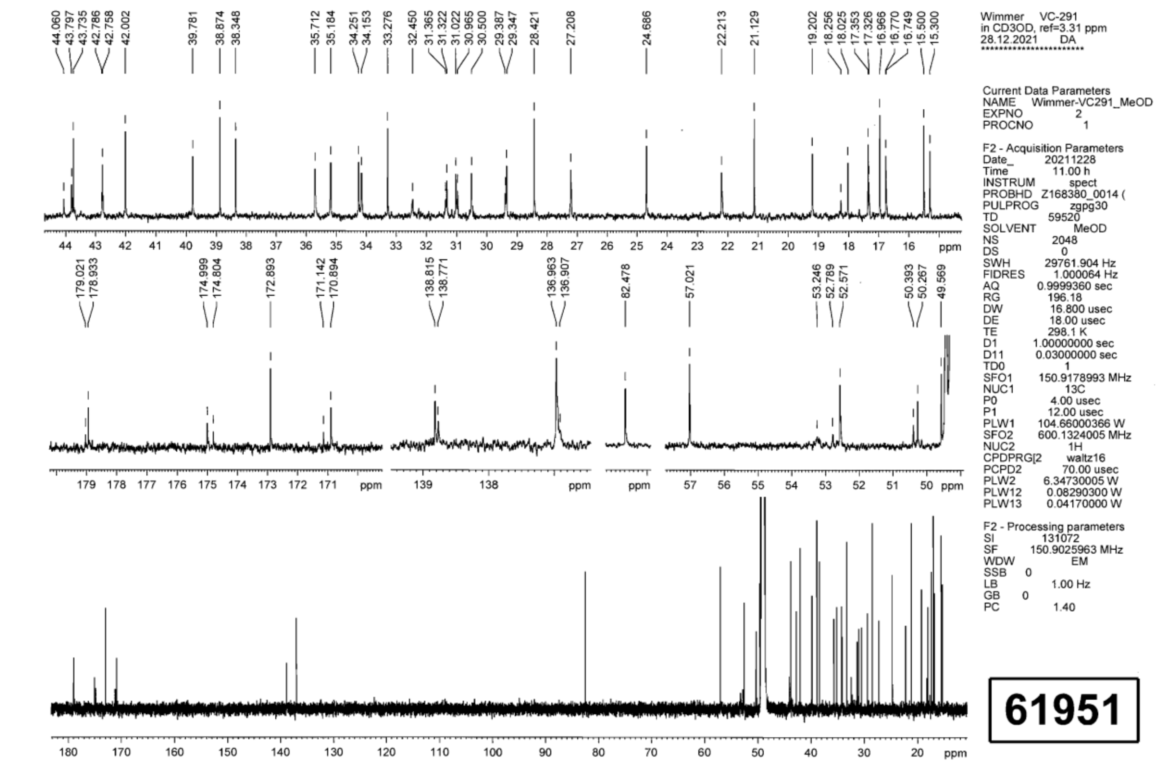
44.060 43.797 43.735 42.786 42.758 42.002 39.781 38.874 38.348 35.712 35.184 34.251 34.153 33.276 32.450 31.365 31.322 31.022 30.965 30.500 29.387 29.347 28.421 27.208 24.686 22.213 21.129 19.202 18.256 18.025 17.353 17.326 16.966 16.770 16.749 15.500 15.300
44 43 42 41 40 39 38 37 36 35 34 33 32 31 30 29 28 27 26 25 24 23 22 21 20 19 18 17 16 ppm
179.021 178.933 174.999 174.804 172.893 171.142 170.894 138.815 138.771 136.963 136.907 82.478 57.021 53.246 52.789 52.571 50.393 50.267 49.569
179 178 177 176 175 174 173 172 171 ppm 139 138 ppm ppm 57 56 55 54 53 52 51 50 ppm
180 170 160 150 140 130 120 110 100 90 80 70 60 50 40 30 20 ppm
Wimmer VC-291
in CD3OD, ref=3.31 ppm
28.12.2021 DA
Current Data Parameters
NAME Wimmer-VC291_MeOD
EXPNO 2
PROCNO 1
F2 - Acquisition Parameters
Date_ 20211228
Time 11.00 h
INSTRUM spect
PROBHD Z168380_0014 (
PULPROG zgpg30
TD 59520
SOLVENT MeOD
NS 2048
DS 0
SWH 29761.904 Hz
FIDRES 1.000064 Hz
AQ 0.9999360 sec
RG 196.18
DW 16.800 usec
DE 18.00 usec
TE 298.1 K
D1 1.00000000 sec
D11 0.03000000 sec
TD0 1
SFO1 150.9178993 MHz
NUC1 13C
P0 4.00 usec
P1 12.00 usec
PLW1 104.66000366 W
SFO2 600.1324005 MHz
NUC2 1H
CPDPRG[2 waltz16
PCPD2 70.00 usec
PLW2 6.34730005 W
PLW12 0.08290300 W
PLW13 0.04170000 W
F2 - Processing parameters
SI 131072
SF 150.9025963 MHz
WDW EM
SSB 0
LB 1.00 Hz
GB 0
PC 1.40
61951

Ethyl (3β)-*N*-[3-(acetyloxy)-28-oxoolean-18-en-28-yl]-L-methionyl-L-alanylglycinate (**21**)

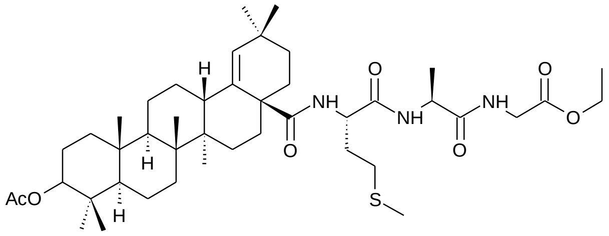

^{1}H NMR (CD_3OD): δ [ppm] 0.75 (3H, s, H27), 0.78 (1H, dd, J_1=1.9 Hz, J_2=11.4 Hz, H5), 0.82 (3H, s, H25), 0.83 (3H, s, H23), 0.88 (3H, s, H24), 0.92 (3H, s, H26), 0.98 (3H, s, H30), 1.01 (3H, s, H29), 1.17 (1H, dt, J_1=3.5 Hz, J_2=3.5 Hz, J_3=13.3 Hz, H15), 1.27 (3H, t, *J*=7.1 Hz, H14'), 1.40 (3H, *J*=7.1 Hz, H10'), 1.74 (2H, dt, J_1=3.6 Hz, J_2=3.6 Hz, J_3=13.2 Hz, H1), 1.83 (2H, ddd, J_1=3.5 Hz, J_2=5.1 Hz, J_3=14.1 Hz, H16), 1.93 (1H, m, J_1=7.0 Hz, J_2=7.0 Hz, J_3=7.0 Hz, J_4=14.2 Hz, H5'), 2.03-2.05 (1H, m, H5'), 2.04 (3H, s, **CH_3**CO), 2.36 (1H, dt, J_1=3.4 Hz, J_2=3.4 Hz, J_3=13.2 Hz, H7), 2.52-2.59 (2H, m, H6'), 3.98 (1H, dd, J_1=5.2 Hz, J_2=18.3 Hz, H11'), 4.02 (1H, dd, J_1=5.4 Hz, J_2=18.3 Hz, H11'), 4.20 (2H, q, *J*=7.1 Hz, H13'), 4.47 (1H, m, J_1=7.2 Hz, J_2=7.2 Hz, J_3=7.2 Hz, H8'), 4.48 (1H, dd, J_1=5.4 Hz, J_2=11.5 Hz, H3), 4.54 (1H, dt, J_1=6.8 Hz, J_2=6.8 Hz, J_3=7.7 Hz, H3'), 5.36 (1H, dd, J_1=0.6 Hz, J_2=1.7 Hz, H19), 6.45 (1H, d, *J*=7.7 Hz, 3'-NH), 6.72 (1H, d, *J*=5.3 Hz, 11'-NH), 6.80 (1H, t, *J*=7.4 Hz, 8'-NH). ^{13}C NMR (CD_3OD): δ [ppm] 14.9 (q, C27), 15.3 (q, C7'), 16.0 (q, C26), 16.5 (q, C25), 16.8 (q, C24), 17.8 (q, C10'), 18.0 (t, C6), 21.0 (t, C11), 21.3 (q, C2'), 23.2 (t, C2), 26.2 (t, C12), 27.9 (q, C23), 28.9 (q, C30), 29.3 (t, C15), 30.2 (d, C6'), 30.9 (t, C5'), 31.0 (q, C29), 32.3 (s, C20), 32.8 (t, C7), 33.4 (t, C21), 33.9 (t, C16), 34.4 (t, C22), 37.1 (s, C10), 37.8 (s, C4), 38.6 (t, C1), 40.7 (s, C8), 41.3 (t, C11'), 41.7 (d, C13), 42.6 (s, C14), 48.3 (s, C17), 48.9 (d, C8'), 51.1 (d, C9), 52.4 (d, C3'), 55.5 (d, C5), 80.9 (d, C3), 136.1 (d, C19), 137.8 (s, C18), 169.5 (s, C12'), 171.0 (s, C1'), 171.0 (s, C4'), 171.9 (s, C9'), 176.5 (s, C28). IR [cm^{-1}]: 3366, 2936, 1690, 1417, 1364, 1244, 1025, 979. MS: *m/z* = 786.5 $[M+H]^+$ (ESI^+, coin voltage 20 V), *m/z* = 784.4 $[M–H]^+$ ($ESI^–$, coin voltage 20 V). For $C_{44}H_{71}N_3O_7S$ (786.12) calcd. C 67.23, H 9.10, N 5.35, S 4.08, found C 67.21, H 9.12, N 5.34, S 4.06.

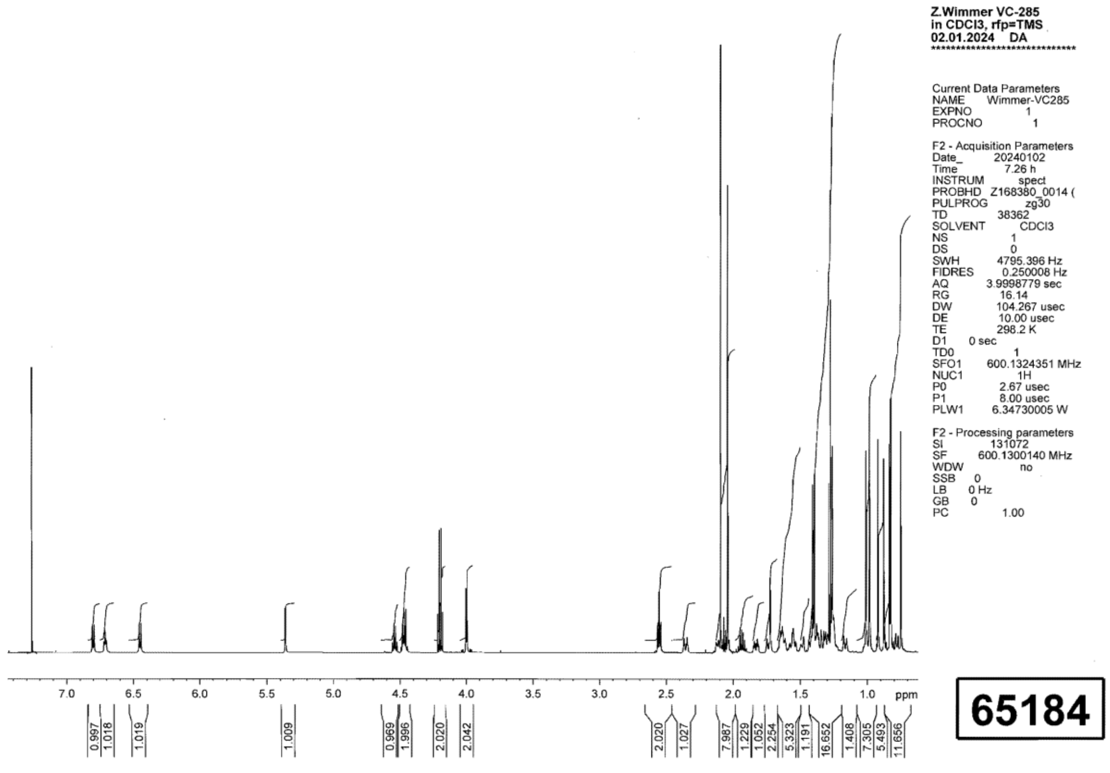
Z.Wimmer VC-285
in CDCl3, rfp=TMS
02.01.2024 DA
Current Data Parameters
NAME Wimmer-VC285
EXPNO 1
PROCNO 1
F2 - Acquisition Parameters
Date_ 20240102
Time 7.26 h
INSTRUM spect
PROBHD Z168380_0014 (
PULPROG zg30
TD 38362
SOLVENT CDCl3
NS 1
DS 0
SWH 4795.396 Hz
FIDRES 0.250008 Hz
AQ 3.9998779 sec
RG 16.14
DW 104.267 usec
DE 10.00 usec
TE 298.2 K
D1 0 sec
TD0 1
SFO1 600.1324351 MHz
NUC1 1H
P0 2.67 usec
P1 8.00 usec
PLW1 6.34730005 W
F2 - Processing parameters
SI 131072
SF 600.1300140 MHz
WDW no
SSB 0
LB 0 Hz
GB 0
PC 1.00
7.0 6.5 6.0 5.5 5.0 4.5 4.0 3.5 3.0 2.5 2.0 1.5 1.0 ppm
0.997 1.018 1.019 1.009 0.969 1.996 2.020 2.042 2.020 1.027 7.987 1.229 1.052 2.254 5.323 1.191 16.652 1.408 7.305 5.493 11.656
65184

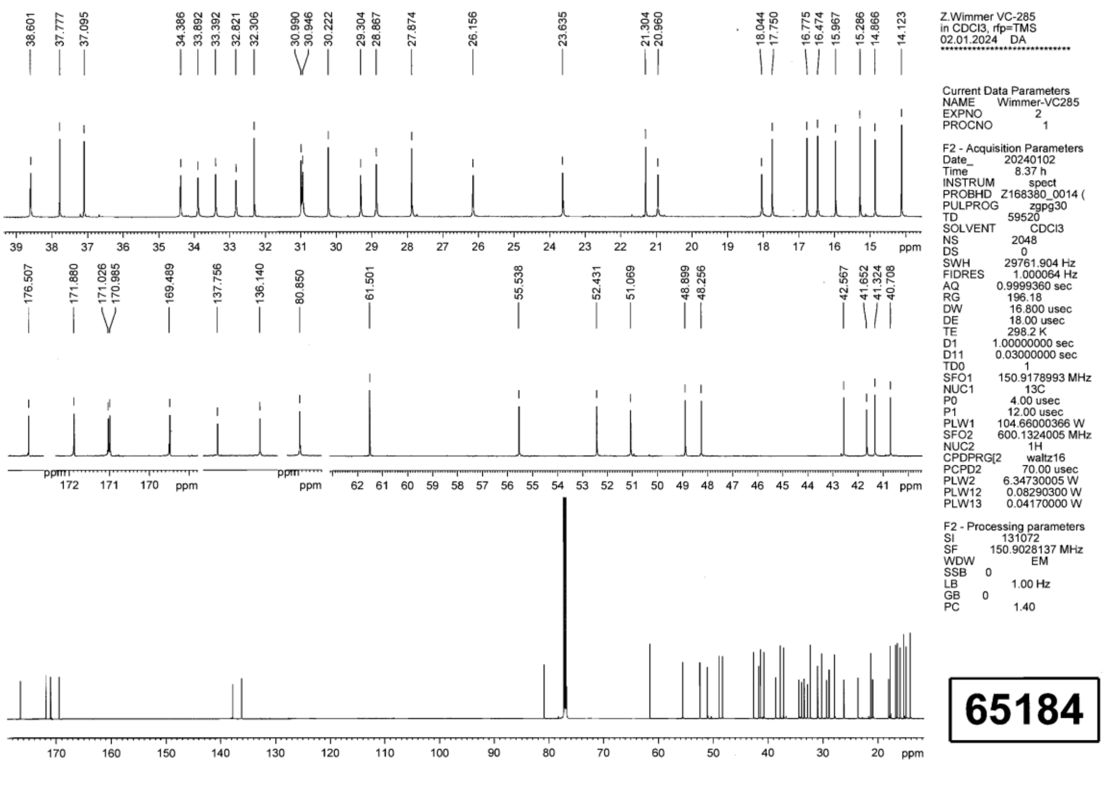
Z.Wimmer VC-285
in CDCl3, rfp=TMS
02.01.2024 DA
38.601 37.777 37.095 34.386 33.892 33.392 32.821 32.306 30.990 30.946 30.222 29.304 28.867 27.874 26.156 23.635 21.304 20.960 18.044 17.750 16.775 16.474 15.967 15.286 14.866 14.123
39 38 37 36 35 34 33 32 31 30 29 28 27 26 25 24 23 22 21 20 19 18 17 16 15 ppm
176.507 171.880 171.026 170.985 169.489 137.756 136.140 80.850 61.501 55.538 52.431 51.069 48.899 48.256 42.567 41.652 41.324 40.708
172 171 170 ppm
ppm
62 61 60 59 58 57 56 55 54 53 52 51 50 49 48 47 46 45 44 43 42 41 ppm
Current Data Parameters
NAME Wimmer-VC285
EXPNO 2
PROCNO 1
F2 - Acquisition Parameters
Date_ 20240102
Time 8.37 h
INSTRUM spect
PROBHD Z168380_0014 (
PULPROG zgpg30
TD 59520
SOLVENT CDCl3
NS 2048
DS 0
SWH 29761.904 Hz
FIDRES 1.000064 Hz
AQ 0.9999360 sec
RG 196.18
DW 16.800 usec
DE 18.00 usec
TE 298.2 K
D1 1.00000000 sec
D11 0.03000000 sec
TD0 1
SFO1 150.9178993 MHz
NUC1 13C
P0 4.00 usec
P1 12.00 usec
PLW1 104.66000366 W
SFO2 600.1324005 MHz
NUC2 1H
CPDPRG[2 waltz16
PCPD2 70.00 usec
PLW2 6.34730005 W
PLW12 0.08290300 W
PLW13 0.04170000 W
F2 - Processing parameters
SI 131072
SF 150.9028137 MHz
WDW EM
SSB 0
LB 1.00 Hz
GB 0
PC 1.40
170 160 150 140 130 120 110 100 90 80 70 60 50 40 30 20 ppm
65184

(3β)-*N*-(3-Hydroxy-28-oxoolean-18-en-28-yl)-L-methionyl-L-alanylglycine (**22**)

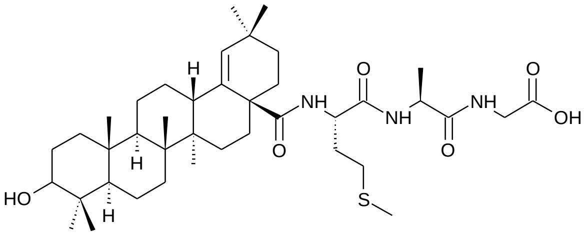

^{1}H NMR (CD_3OD): δ [ppm] 0.72 (1H, dd, J_1=1.8 Hz, J_2=11.5 Hz, H5), 0.76 (3H, s, H25), 0.81 (3H, s, H27), 0.90 (3H, s, H24), 0.95 (3H, s, H23), 0.97 (3H, s, H26), 1.00 (3H, s, H30), 1.05 (3H, s, H29), 1.39 (3H, d, *J*=7.1 Hz, H8'), 1.76 (2H, dt, J_1=4.0 Hz, J_2=4.0 Hz, J_3=13.0 Hz, H1), 1.82 (2H, dt, J_1=4.5 Hz, J_2=4.1 Hz, J_3=14.0 Hz, H2), 1.89-1.97 (2H, m, H3'), 2.08 (3H, s, H5'), 2.23 (1H, ddd, J_1=1.8 Hz, J_2=3.0 Hz, J_3=12.5 Hz, H13), 2.48-2.54 (2H, m, H4'), 3.14 (1H, dd, J_1=4.8 Hz, J_2=11.6 Hz, H3), 3.73 (1H, d, *J*=17.3 Hz, H9'), 3.78 (1H, d, *J*=17.3 Hz, H9'), 4.42 (1H, q, J=7.1 Hz, H6'), 5.39 (1H, bd, *J*=1.8 Hz, H19). ^{13}C NMR (CD_3OD): δ [ppm] 15.4 (q, C27), 15.4 (q, C5'), 16.1 (q, C25), 16.7 (q, C26), 17.3 (q, C24), 18.2 (q, C8'), 19.3 (t, C6), 22.2 (t, C11), 27.3 (t, C2), 28.1 (t, C12), 28.6 (q, C23), 29.2 (q, C30), 30.4 (t, C15), 31.2 (q, C29), 31.1 (t, C7), 33.3 (s, C20), 33.6 (t, C4'), 34.1 (t, C21), 34.5 (t, C16), 35.0 (t, C22), 35.8 (t, C3'), 38.4 (s, C10), 40.0 (t, C1), 40.3 (s, C4), 42.0 (s, C8), 42.8 (d, C13), 43.7 (s, C14), 44.4 (t, C9'), 49.6 (s, C17), 50.4 (d, C6'), 52.6 (d, C9), 53.6 (d, C1'), 57.0 (d, C5), 79.6 (d, C3), 136.9 (d, C19), 139.2 (d, C18), 173.0 (s, C10'), 174.3 (s, C7'), 176.8 (s, C2'), 178.1 (s, C28). IR [cm^{-1}]: 3387, 2940, 2870, 1679, 1528, 1477, 1419, 1366,1168. MS: *m/z* = 716.4 $[M+H]^+$ (ESI^+, coin voltage 20 V), *m/z* = 714.3 $[M–H]^+$ (ESI^-, coin voltage 20 V). For $C_{40}H_{65}N_3O_6S$ (716.03) calcd. C 67.10, H 9.15, N 5.87, S 4.48, found C 67.07, H 9.16, N 5.89, S 4.46.

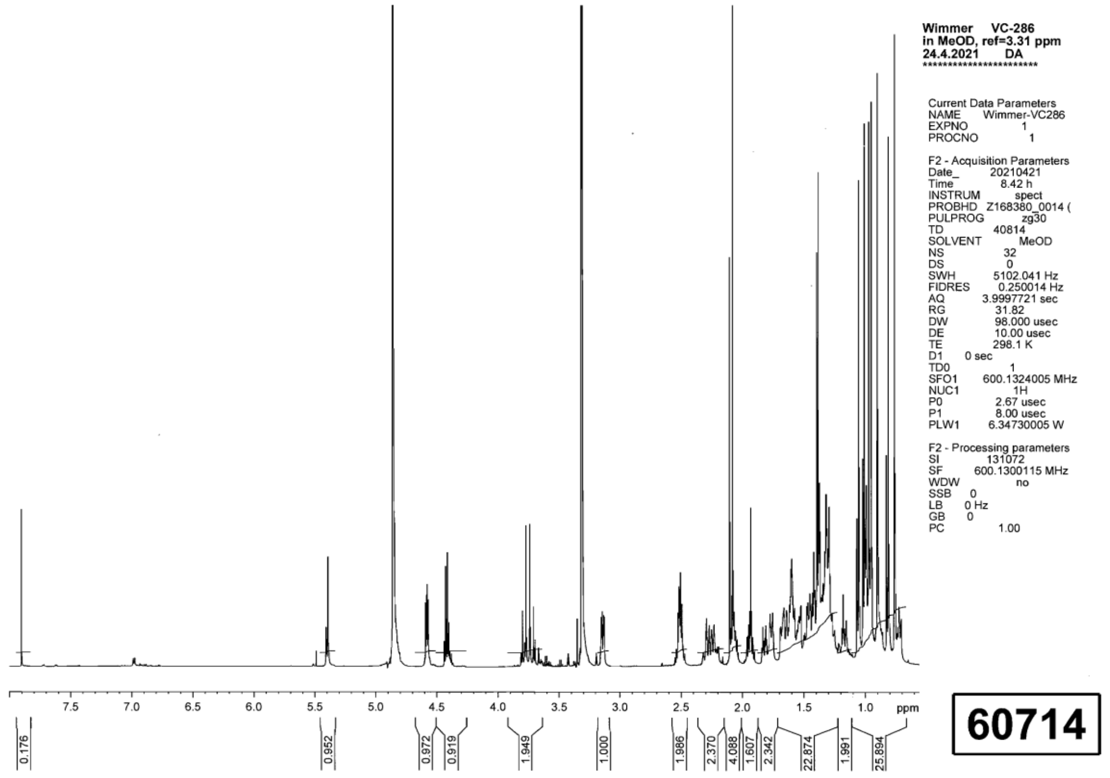
Wimmer VC-286
in MeOD, ref=3.31 ppm
24.4.2021 DA
Current Data Parameters
NAME Wimmer-VC286
EXPNO 1
PROCNO 1
F2 - Acquisition Parameters
Date_ 20210421
Time 8.42 h
INSTRUM spect
PROBHD Z168380_0014 (
PULPROG zg30
TD 40814
SOLVENT MeOD
NS 32
DS 0
SWH 5102.041 Hz
FIDRES 0.250014 Hz
AQ 3.9997721 sec
RG 31.82
DW 98.000 usec
DE 10.00 usec
TE 298.1 K
D1 0 sec
TD0 1
SFO1 600.1324005 MHz
NUC1 1H
P0 2.67 usec
P1 8.00 usec
PLW1 6.34730005 W
F2 - Processing parameters
SI 131072
SF 600.1300115 MHz
WDW no
SSB 0
LB 0 Hz
GB 0
PC 1.00
60714

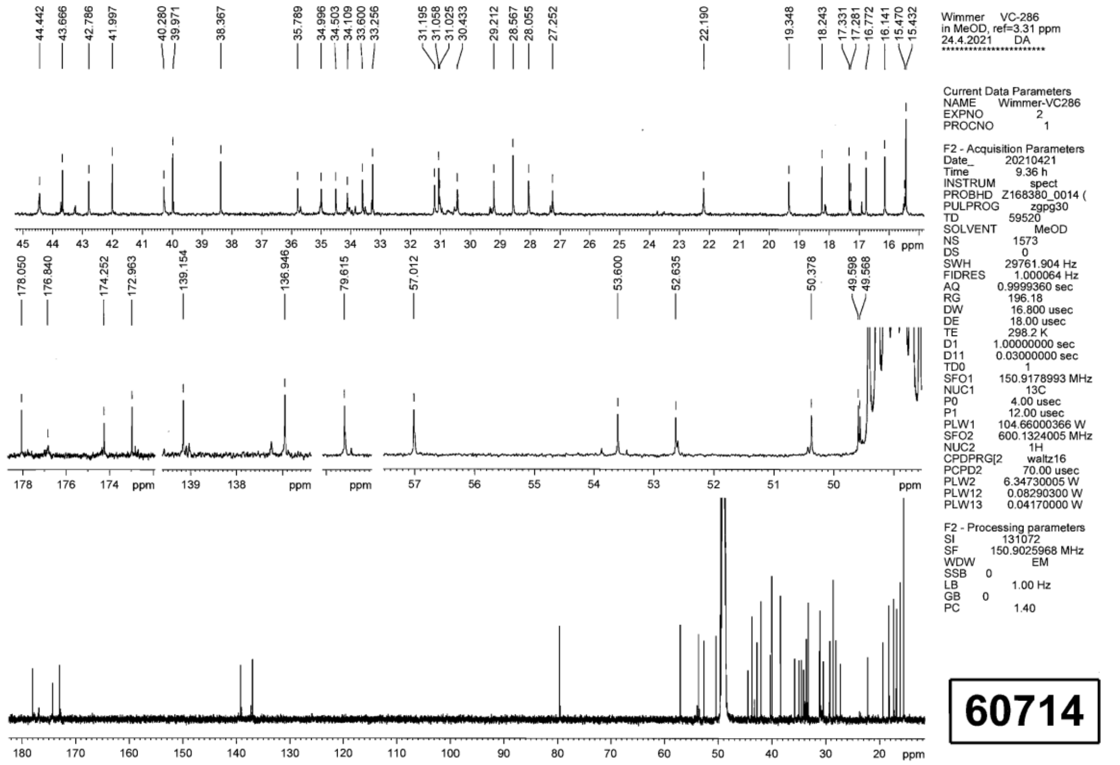
Wimmer VC-286
in MeOD, ref=3.31 ppm
24.4.2021 DA
Current Data Parameters
NAME Wimmer-VC286
EXPNO 2
PROCNO 1
F2 - Acquisition Parameters
Date_ 20210421
Time 9.36 h
INSTRUM spect
PROBHD Z168380_0014 (
PULPROG zgpg30
TD 59520
SOLVENT MeOD
NS 1573
DS 0
SWH 29761.904 Hz
FIDRES 1.000064 Hz
AQ 0.9999360 sec
RG 196.18
DW 16.800 usec
DE 18.00 usec
TE 298.2 K
D1 1.00000000 sec
D11 0.03000000 sec
TD0 1
SFO1 150.9178993 MHz
NUC1 13C
P0 4.00 usec
P1 12.00 usec
PLW1 104.66000366 W
SFO2 600.1324005 MHz
NUC2 1H
CPDPRG[2 waltz16
PCPD2 70.00 usec
PLW2 6.34730005 W
PLW12 0.08290300 W
PLW13 0.04170000 W
F2 - Processing parameters
SI 131072
SF 150.9025968 MHz
WDW EM
SSB 0
LB 1.00 Hz
GB 0
PC 1.40
60714

(3β)-*N*-[3-(Acetyloxy)-28-oxoolean-18-en-28-yl]-L-methionyl-L-alanylglycine (**23**)

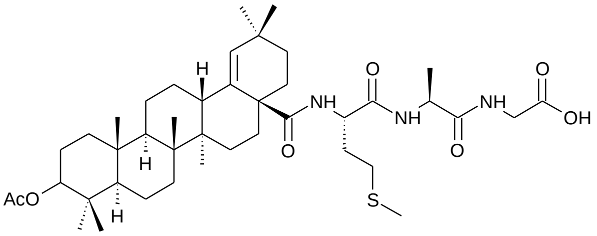

^{1}H NMR (CD_3OD): δ [ppm] 0.82 (3H, s, H27), 0.84 (1H, dd, J_1=5.1 Hz, J_2=11.3 Hz, H5), 0.86 (3H, s, H23), 0.87 (3H, s, H25), 0.93 (3H, s, H24), 0.98 (3H, s, H26), 1.01 (3H, s, H30), 1.05 (3H, s, H29), 1.17 (2H, dt, J_1=3.3 Hz, J_2=3.3 Hz, J_3=13.0 Hz, H15), 1.39 (2H, d, *J*=7.1 Hz, H4'), 1.79 (2H, dt, J_1=3.6 Hz, J_2=3.6 Hz, J_3=13.3 Hz, H1), 1.83 (2H, dt, J_1=4.3 Hz, J_2=4.3 Hz, J_3=14.0 Hz, H16), 1.94 (3H, dddd, J_1=6.0 Hz, J_2=8.2 Hz, J_1=8.2 Hz, J_1=10.5 Hz, H8'), 2.03 (3H, s, H12', **CH_3**CO), 2.04-2.10 (3H, m, H8'), 2.08 (3H, s, H10'), 2.23-2.26 (1H, m, H13), 2.47-2.55 (2H, m, H9'), 4.42 (3H, q, *J*=7.1 Hz, H3'), 4.46 (1H, dd, J_1=5.1 Hz, J_2=11.3 Hz, H3), 4.58 (1H, ddd, J_1=5.0 Hz, J_2=6.0 Hz, J_3=8.2 Hz, H6'), 5.40 (1H, bd, *J*=1.8 Hz, H19). ^{13}C NMR (CD_3OD): δ [ppm] 15.4 (q, C10'), 15.5 (q, C27), 16.8 (q, C26), 17.0 (q, C25), 17.4 (q, C24), 18.3 (q, C4'), 19.2 (t, C6), 21.1 (q, C12'), 22.2 (t, C11), 24.7 (t, C2), 27.2 (t, C12), 28.4 (q, C23), 29.2 (q, C30), 30.4 (t, C15), 31.1 (t, C9'), 31.2 (q, C29), 33.3 (s, C20), 33.6 (t, C8'), 34.1 (t, C7), 34.5 (t, C21), 35.0 (t, C16), 35.7 (t, C22), 38.3 (s, C10), 38.9 (s, C4), 39.8 (t, C1), 42.0 (s, C8), 42.8 (d, C13), 43.7 (s, C14), 43.7 (t, C1'), 49.6 (s, C17), 50.3 (d, C3'), 52.5 (d, C9), 53.6 (d, C6'), 57.0 (d, C5), 82.4 (d, C3), 137.0 (d, C19), 139.1 (s, C18), 172.9 (s, C11'), 174.5 (s, C7'), 173.0 (s, C2'), 176.6 (s, C5'), 178.1 (s, C28). IR [cm^{-1}]: 3379, 2945, 2875, 1678, 1525, 1476, 1419, 1365, 1247, 1168, 1027. MS: *m/z* = 758.6 $[M+H]^+$ (ESI^+, coin voltage 20 V), *m/z* = 756.6 $[M–H]^+$ (ESI^-, coin voltage 20 V). For $C_{42}H_{67}N_3O_7S$ (758.06) calcd. C 66.54, H 8.91, N 5.54, S 4.23, found C 66.56, H 8.89, N 5.56, S 4.20.

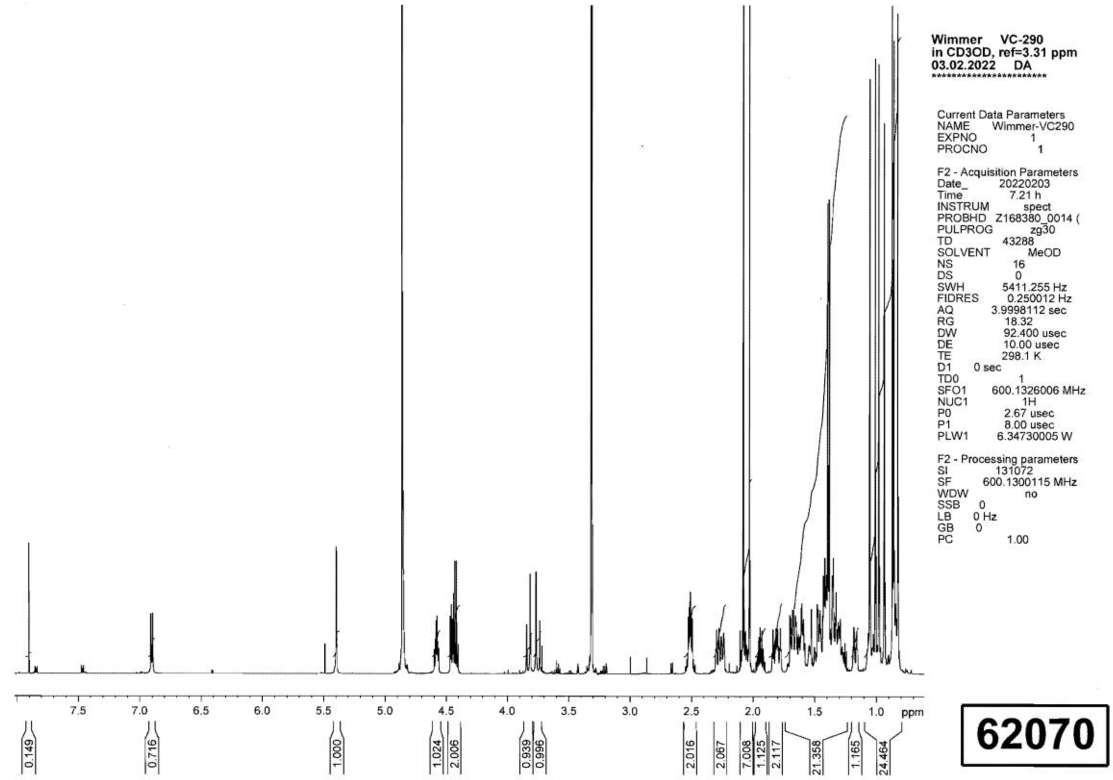
Wimmer VC-290
in CD3OD, ref=3.31 ppm
03.02.2022 DA
Current Data Parameters
NAME Wimmer-VC290
EXPNO 1
PROCNO 1
F2 - Acquisition Parameters
Date_ 20220203
Time 7.21 h
INSTRUM spect
PROBHD Z168380_0014 (
PULPROG zg30
TD 43288
SOLVENT MeOD
NS 16
DS 0
SWH 5411.255 Hz
FIDRES 0.250012 Hz
AQ 3.9998112 sec
RG 18.32
DW 92.400 usec
DE 10.00 usec
TE 298.1 K
D1 0 sec
TD0 1
SFO1 600.1326006 MHz
NUC1 1H
P0 2.67 usec
P1 8.00 usec
PLW1 6.34730005 W
F2 - Processing parameters
SI 131072
SF 600.1300115 MHz
WDW no
SSB 0
LB 0 Hz
GB 0
PC 1.00
7.5 7.0 6.5 6.0 5.5 5.0 4.5 4.0 3.5 3.0 2.5 2.0 1.5 1.0 ppm
0.149 0.716 1.000 1.024 2.006 0.939 0.996 2.016 2.067 7.008 1.125 2.117 21.358 1.165 24.464
62070

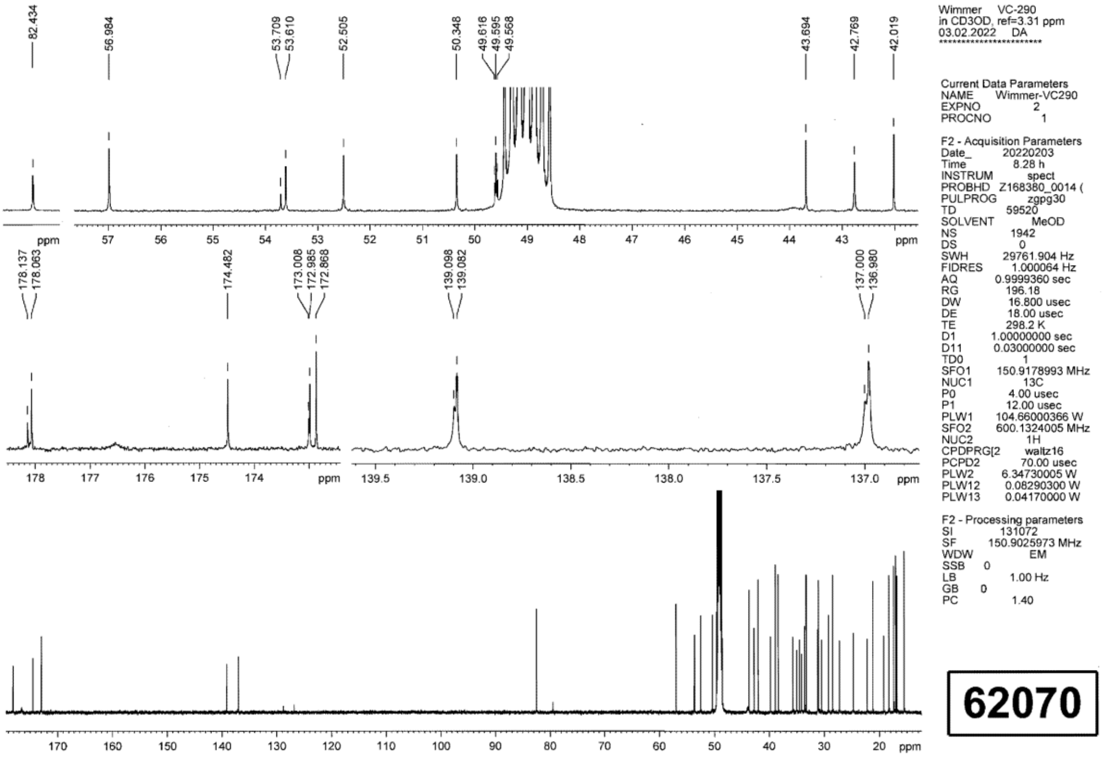
82.434 56.984 53.709 53.610 52.505 50.348 49.616 49.595 49.568 43.694 42.769 42.019
Wimmer VC-290
in CD3OD, ref=3.31 ppm
03.02.2022 DA
Current Data Parameters
NAME Wimmer-VC290
EXPNO 2
PROCNO 1
F2 - Acquisition Parameters
Date_ 20220203
Time 8.28 h
INSTRUM spect
PROBHD Z168380_0014 (
PULPROG zgpg30
TD 59520
SOLVENT MeOD
NS 1942
DS 0
SWH 29761.904 Hz
FIDRES 1.000064 Hz
AQ 0.9999360 sec
RG 196.18
DW 16.800 usec
DE 18.00 usec
TE 298.2 K
D1 1.00000000 sec
D11 0.03000000 sec
TD0 1
SFO1 150.9178993 MHz
NUC1 13C
P0 4.00 usec
P1 12.00 usec
PLW1 104.66000366 W
SFO2 600.1324005 MHz
NUC2 1H
CPDPRG[2 waltz16
PCPD2 70.00 usec
PLW2 6.34730005 W
PLW12 0.08290300 W
PLW13 0.04170000 W
F2 - Processing parameters
SI 131072
SF 150.9025973 MHz
WDW EM
SSB 0
LB 1.00 Hz
GB 0
PC 1.40
ppm 57 56 55 54 53 52 51 50 49 48 47 46 45 44 43 ppm
178.137 178.063 174.482 173.008 172.985 172.868 139.098 139.082 137.000 136.980
178 177 176 175 174 ppm 139.5 139.0 138.5 138.0 137.5 137.0 ppm
170 160 150 140 130 120 110 100 90 80 70 60 50 40 30 20 ppm
62070

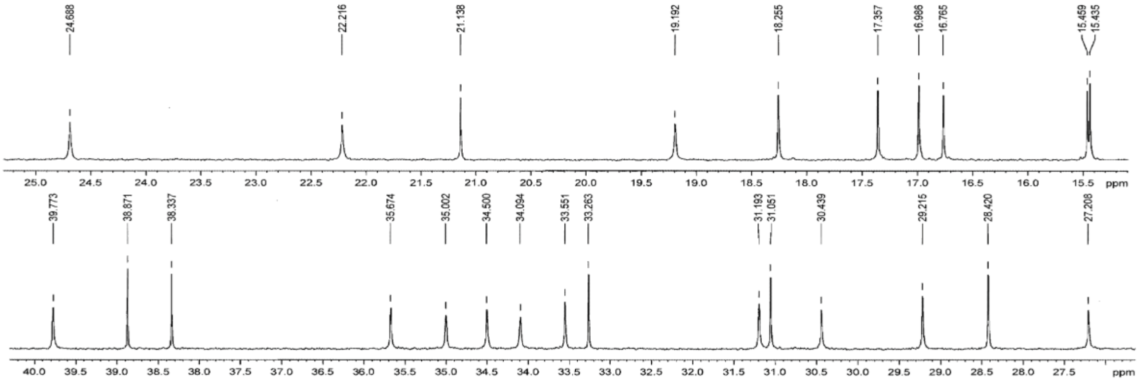
24.688
22.216
21.138
19.192
18.255
17.357
16.986
16.765
15.459
15.435
ppm
39.773
38.871
38.337
35.674
35.002
34.500
34.094
33.551
33.263
31.193
31.051
30.439
29.215
28.420
27.208
ppm

2. Figure S1. Inhibition effect of **16** on *S. aureus* (**A**) and *E. faecalis* (**B**).

A.

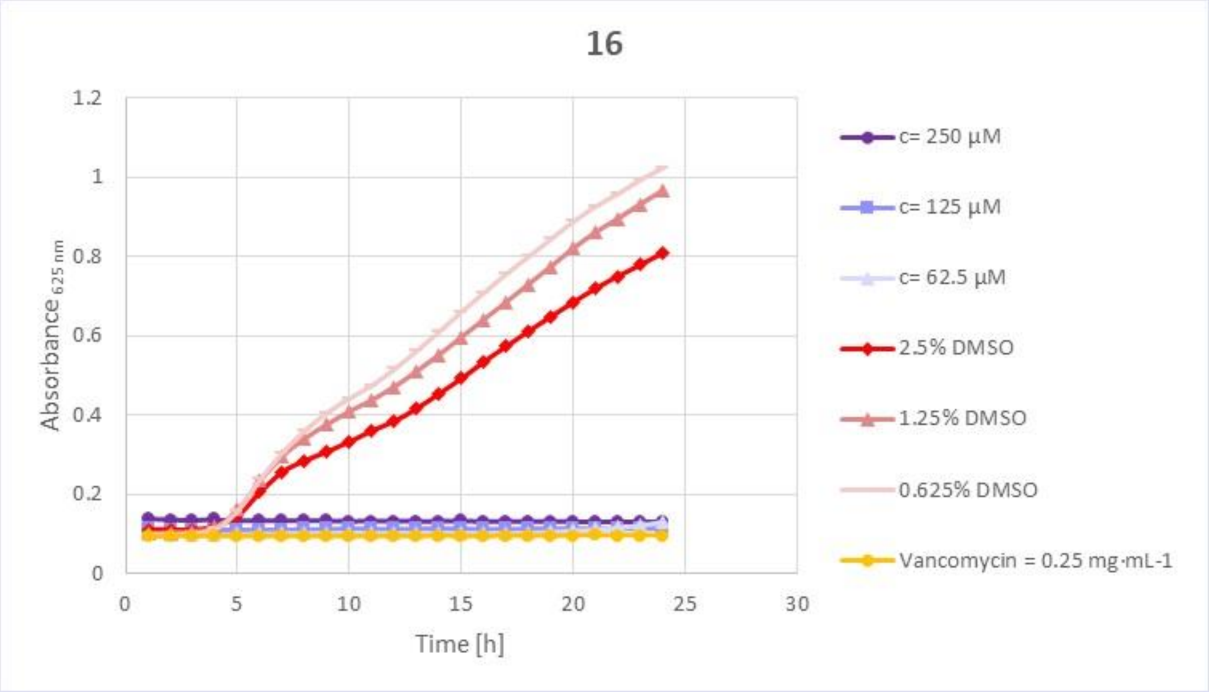

B.

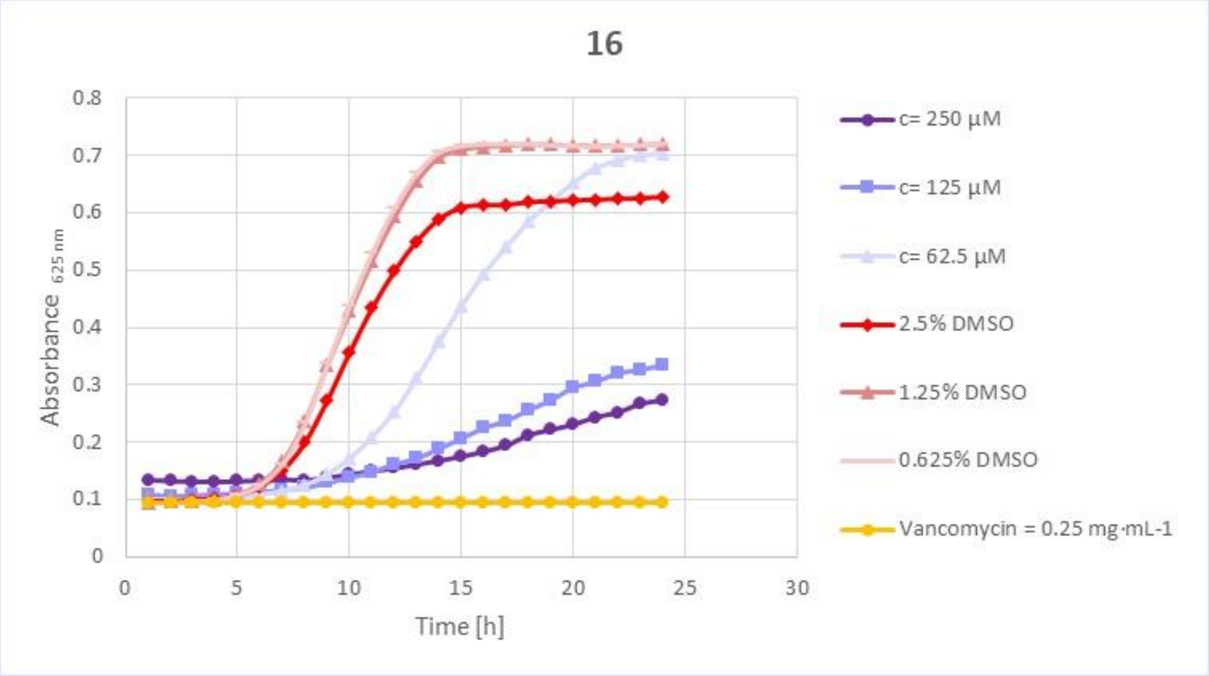

3. Table S1. Inhibition of *P. aeruginosa* and *E. coli* in the dilution test [%], calculated to DMSO.

Compound	Inhibition of *P. aeruginosa* [%]			Inhibition of *E. coli* [%]		
	Concentration of the compound [μM]			Concentration of the compound [μM]		
	250 μM	125 μM	62.5 μM	250 μM	125 μM	62.5 μM
**11**	inactive	13.43	7.27	1.43	inactive	inactive
**12**	7.23	20.23	16.75	34.57	14.92	inactive
**13**	inactive	inactive	inactive	inactive	inactive	inactive
**14**	inactive	1.00	inactive	27.76	2.83	inactive
**15**	inactive	inactive	inactive	inactive	inactive	1.87
**16**	inactive	inactive	inactive	20.70	3.10	2.91
**18**	inactive	inactive	inactive	2.78	3.54	inactive
**19**	inactive	inactive	inactive	6.74	0.67	6.59
**20**	8.33	16.57	7.51	14.87	inactive	inactive
**21**	inactive	inactive	inactive	7.30	inactive	inactive
**22**	inactive	inactive	inactive	inactive	inactive	inactive
**23**	inactive	inactive	inactive	0.51	1.79	3.55
vancomycin	100	100	100	-	-	-
kanamycin	-	-	-	100	100	100

4. Figures S2 and S3. Antiviral activity and cytotoxicity of the studied compounds.

To determine the anti-HIV-1 and anti-HSV-1 activity of prepared derivatives of moronic acid and morolic acid, the ability of derivatives to inhibit virus-induced cytopathic effect (CPE) in MT-4 and Vero cells, respectively, was measured. While the results of testing the compound **23** was presented in the main text of this paper (Table 2; Figure 1), the results obtained with the other compounds of the studied series, as well as those obtained with the parent triterpenoids (**11** and **12**), are presented in ESI, Figures S2 and S3.

Figure S2. Antiviral and cytotoxicity evaluation of the compounds of the studied series. Anti-HIV-1 activity (**A**) and cytotoxicity (**B**) in MT-4 cells.

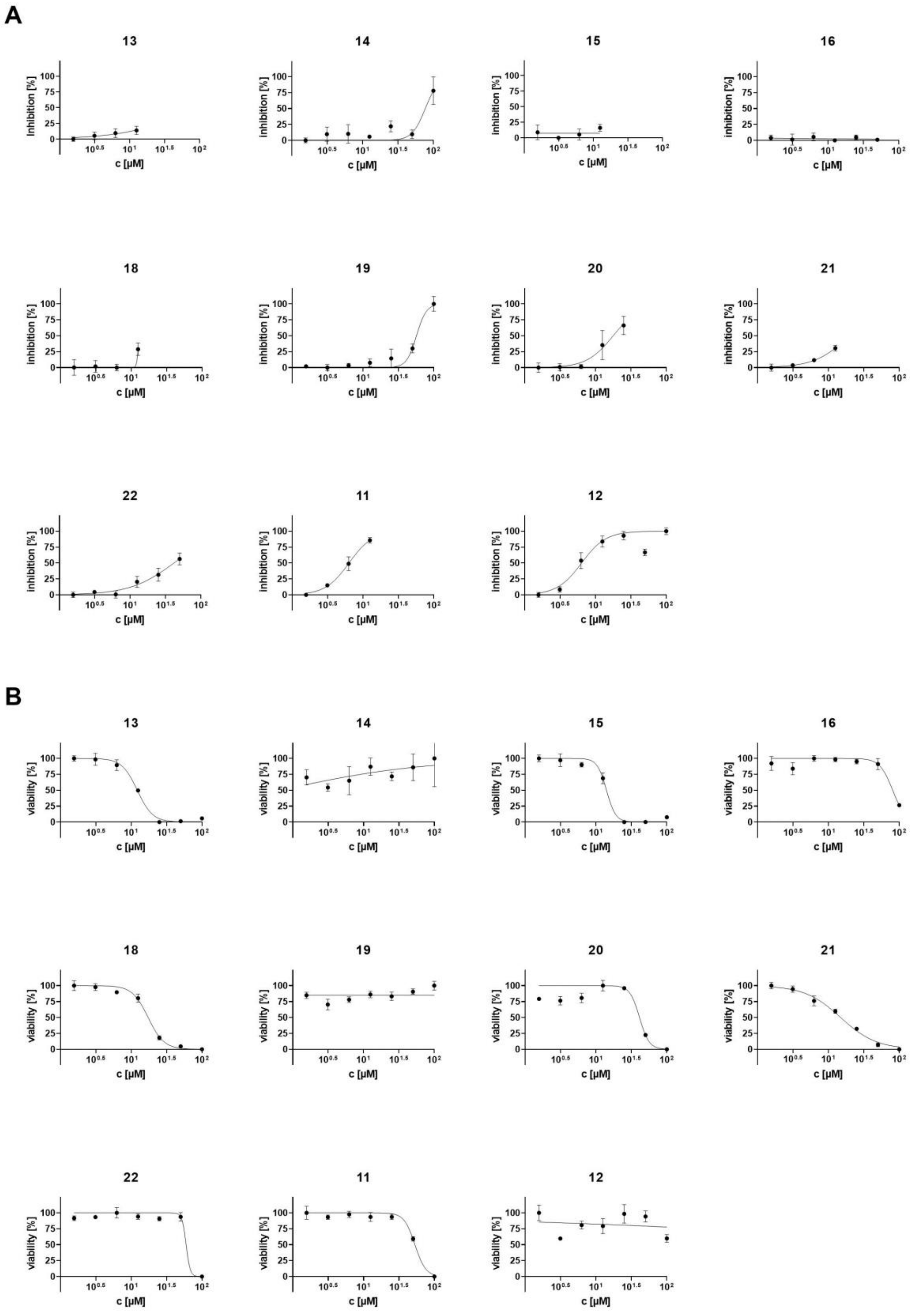

Figure S3. Antiviral and cytotoxicity evaluation of the compounds of the studied series. Anti-HSV-1 activity (**A**) and cytotoxicity (**B**) in Vero cells.

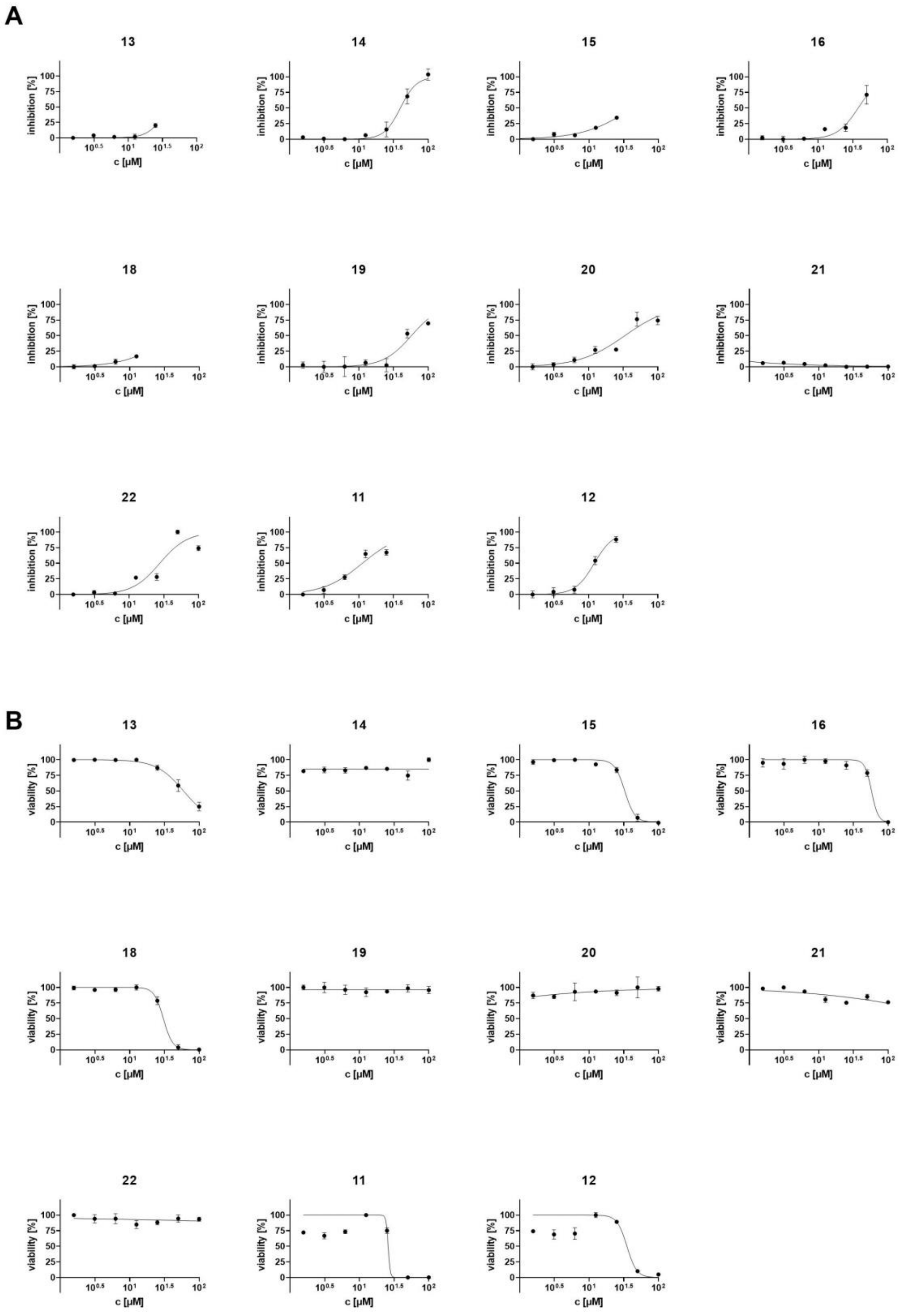

Supplement: MD-016-D4MD00742E-s001 [file MD-016-D4MD00742E-s001.pdf]
